# Structural mechanism of LINE-1 target-primed reverse transcription

**DOI:** 10.1126/science.ads8412

**Published:** 2025-04-25

**Authors:** George E. Ghanim, Hongmiao Hu, Jerome Boulanger, Thi Hoang Duong Nguyen

**Affiliations:** 1 MRC Laboratory of Molecular Biology; Cambridge CB2 0QH, UK

## Abstract

Long interspersed element-1 (LINE-1) retrotransposons are the only active autonomous transposable elements in humans. They propagate by reverse transcribing their mRNA into new genomic locations by a process called target-primed reverse transcription (TPRT). Here, we present four cryo-electoron microscopy structures of the human LINE-1 TPRT complex, revealing the conformational dynamics of ORF2p and its extensive remodeling of the target DNA for TPRT initiation. We observe nicking of the DNA second strand during reverse transcription of the first strand. Structure prediction identifies high-confidence binding sites for LINE-1-associated factors, namely PCNA and PABPC1, on ORF2p. Together with our structural data, this suggests a mechanism by which these factors regulate retrotransposition and proposes a model for TPRT that accounts for retrotransposition outcomes observed in cells.

Retrotransposons are genetic sequences that can move through a host genome by means of an RNA intermediate (1). Long interspersed element-1 (LINE-1) and *Alu* elements are the most abundant retrotransposable elements within the human genome, constituting nearly 30% of the genome by sequence (2). Although most are inactive, a small subset of LINE-1s and Alus can still mobilize (3–6). Consequently, this mobility can lead to significant genetic mutations. Retrotransposition results in numerous genetic diseases (7), is thought to drive oncogenic rearrangements in certain cancers (8), and is linked to age-related inflammation (9). Beyond their impact on health, there has been renewed interest in developing retrotransposons and other transposable elements for biotechnological applications (10–15).

LINE-1 retrotransposition is performed by a ribonucleoprotein (RNP) complex composed of the LINE-1 mRNA bound by two encoded proteins, ORF1p and ORF2p (Fig. 1, A and B) (16, 17). ORF1p acts as a cytoplasmic RNA chaperone (18), whereas ORF2p possesses both endonuclease (EN) (19) and reverse transcriptase (RT) activities (20). New genomic copies of LINE-1 are generated through a process called target-primed reverse transcription (TPRT) (21). During TPRT, ORF2p nicks the first ‘bottom’ strand of a 5'-TT|AAAA-3' target DNA sequence, exposing a 3' end that primes reverse transcription of the LINE-1 mRNA (Fig. 1B) (19, 22). The subsequent nicking of the second ‘top’ DNA strand, followed by second-strand synthesis, results in a new genomic copy of the retrotransposon. The staggered nicking of the top strand relative to the bottom strand gives rise to characteristic 7–20 nucleotide (nt) target-site duplications (TSD) flanking the retrotransposon (23, 24).

Recent landmark structures of ORF2p assembled on short RNA-DNA duplexes provide insights into how the first strand of cDNA synthesis is extended (25, 26). However, these studies did not capture the TPRT complex because they had used only single-stranded DNA substrates rather than an authentic double-stranded target DNA. As a result, it remains unclear how ORF2p handles the LINE-1 mRNA and target DNA during TPRT. Additionally, insights into top strand nicking by ORF2p cannot be inferred from these structures (25, 26). Whether or when the top strand is nicked by the ORF2p endonuclease domain, and how TSDs of varying lengths arise, remain unknown.

## A target DNA intermediate stimulates TPRT

To understand the biochemical requirements for TPRT, we first purified ORF2p from baculovirus-infected insect cells (fig. S1A) and tested its TPRT activity *in vitro* using an RNA substrate and a fluorescently labeled pre-nicked target DNA (fig. S1B) (see below). Although purified ORF2p displayed TPRT activity, our initial preparations were ~99.9 % inactive and would not suffice for structural characterizations. Therefore, we used an activity-based purification approach to isolate fractions of ORF2p with high specific TPRT activity (see Methods) (fig. S1C).

**Fig. 1:**
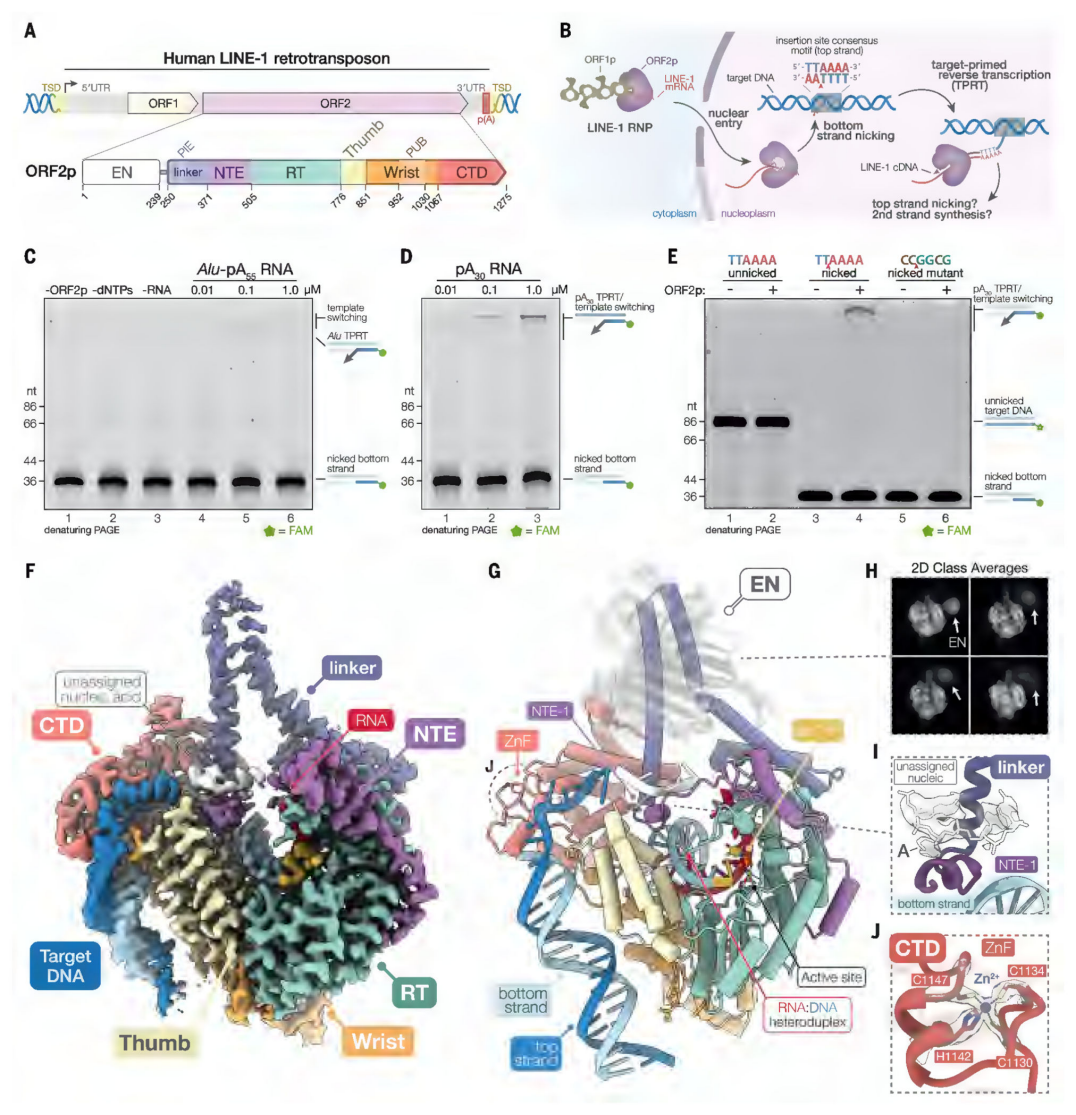
Cryo-EM structure of the LINE-1 TPRT complex. (**A**) Organization of the human LINE-1 retrotransposon and domains of ORF2p. TSD, target site duplication; EN, endonuclease; PIE, PABC interacting and essential element; linker, EN linker; NTE, N-terminal extension; RT, reverse transcriptase; PUB, PCNA unusual binding site; CTD, C-terminal segment domain. (**B**) Schematic of LINE-1 retrotransposition. (**C**) Denaturing gel showing target primed reverse transcription (TPRT) activity with an *Alu* RNA template. (**D**) Denaturing gel showing TPRT activity with a 30 nt poly(A) RNA (pA_30_). (**E**) Denaturing gel showing the effects of unnicked, pre-nicked, or mutated pre-nicked target DNA substrate on TPRT activity. (**F**) Composite 2.3 Å cryo-EM reconstruction of the LINE-1 TPRT complex. Linker and target DNA densities were blurred to highlight flexible features. (**G**) Atomic model of the LINE-1 TPRT complex. ZnF, zinc finger. (**H**) 2D class averages showing flexibility of the EN domain. (**I**) Unassigned nucleic acid contacts the NTE. Cryo-EM density is shown as a transparent surface and blurred to highlight flexible features. (**J**) CTD zinc finger. Cryo-EM density is shown as a transparent surface.

To identify an ideal RNA for structural studies, we performed TPRT assays with either an *Alu* RNA or a 30 nt poly(A) (pA_30_) RNA substrates (fig. S1B). TPRT activity and low levels of template jumping were only observed in the presence of dNTPs and an *Alu* RNA substrate (Fig. 1C). However, increasing concentrations of *Alu* RNA inhibited TPRT activity (Fig. 1C, lanes 4–6). Unlike the *Alu* RNA, the pA_30_ RNA resulted in higher overall activity, while increasing concentrations did not inhibit, but rather stimulated TPRT activity (Fig. 1D). The pA_30_ substrate was also highly efficient for template jumping, producing large TPRT products (Fig. 1D). Given the stimulation, the pA_30_ RNA was used in subsequent assays and in TPRT complex formation.

We next explored the influence of the target DNA architecture on TPRT activity. The target DNA for our assays was derived from the human factor VIII gene, at the locus where *de novo* LINE-1 insertions were first identified (27) and was idealized to introduce a stronger EN motif at the insertion site (fig. S1B). Additionally, the target DNA was pre-nicked on the bottom strand of the TTAAAA insertion consensus. This substrate mimics an intermediate of retrotransposition after bottom strand nicking by the EN domain of ORF2p (fig. S1B). Idealization of the target DNA subtly stimulated TPRT activity (fig. S1D, lanes 4 and 6). As suggested by previous studies, a pre-nicked target DNA greatly stimulated TPRT activity (Fig. 1E, lane 4) (28). In contrast, the levels of TPRT activity on unnicked DNA substrates were nearly imperceptible under the reaction conditions tested (Fig. 1E, lane 2). Mutating the TTAAAA consensus sequence to CCGGCG in the nicked substrate abolished the stimulation (Fig. 1E, lane 6). This demonstrates that a nicked DNA substrate alone is insufficient for TPRT and implies some sequence specificity in the target site of ORF2p. This is consistent with a previously proposed model where complementarity between target DNA primer and RNA template influences the efficiency of reverse transcription (29, 30).

We further evaluated the TPRT activity of our purified ORF2p on alternative target DNA designs, including substrates with a 3' overhang used in previous studies (26). While these substrates supported bottom strand nicking and TPRT activity (fig. S1E, lanes 2 and 4), this activity was abolished if the substrates were made fully double-stranded (fig. S1F, lanes 2 and 6). TPRT activity was restored when the double-stranded substrates were pre-nicked on the bottom strand (fig. S1F, lanes 4 and 8). It remains unclear whether substrates with a 3' overhang bypass the physiological requirements for retrotransposition or represent authentic physiological targets. Similar observations have been reported with other nucleoprotein reactions, where alternative DNA substrates (e.g. pre-nicked, short flanking DNA, or non-complementary flanking DNA) bypass physiological requirements (31, 32). Our observations highlight the importance of substrate design in understanding the mechanistic requirements of retrotransposition.

## Cryo-EM structure and architecture of ORF2p in the TPRT complex

To understand the molecular basis underlying TPRT, we implemented a strategy to assemble and purify active ORF2p with pA_30_ RNA, the pre-nicked target DNA and the chain terminator 2′,3′-dideoxythymidine triphosphate (ddTTP) for structure determination by cryo-EM (fig. S2). We obtained a 2.3 Å resolution reconstruction of the human ORF2p TPRT complex stalled after initiation (Fig. 1F, figs. S3–S5, and table S1).

The domains of ORF2p adopt a basket-like shape that accommodates the RNA-DNA duplex in the RT active site (Fig. 1, F and G), as observed in the previous structures (Supplementary text) (25, 26). ORF2p can be divided into six structural domains: an N-terminal EN domain; an EN linker domain (linker) (26); an N-terminal extension (NTE) domain (26); an RT domain and proceeding thumb subdomain; the wrist domain (25); and the C-terminal segment domain (CTD) (33), (Fig. 1, A, F and G).

The EN domain belongs to a larger class of apurinic/apyrimidinic endonucleases-like domains and connects to the rest of ORF2p by a short flexible linker - but was poorly resolved in our consensus reconstruction due to its apparent flexibility (Fig. 1H). Following the EN domain are the linker domain and NTE domain. The NTE domain has been implicated in template switching (34), while the linker domain features two long helices that form the “handle” of the basket-like architecture (Fig. 1, F and G). Together, the linker and NTE regions (residues 240–440) are also collectively referred to as the ‘tower’ domain (25).

A region of the NTE domain, termed NTE-1 (residues 362–381) (Fig. 1G, NTE-1), resembles that in the related *Bombyx mori* R2 encoded ORF (*Bm*R2) (35) and alpha helix-1 (residues 953–958) of the C-terminal extension domain of the human telomerase reverse transcriptase (TERT) (fig. S6, A and B) (36). Analogous to the *Bm*R2 NTE-1 contacts with its target DNA and template RNA, ORF2p NTE-1 contacts both the target DNA and another nucleic acid strand (Fig. 1I, unassigned nucleic acid, and fig. S6A). The identity of this nucleic acid strand remains unclear because it was disconnected from the surrounding nucleic acid densities. We noted that this ambiguous density resembled adenosine bases and adenosine base-specific interactions, suggesting that it may be part of the pA_30_ RNA.

The RT and thumb lie at the core of basket and together adopt the right-hand fold characteristic to DNA polymerases and reverse transcriptases (Fig. 1, F and G) (37). The mechanism for DNA synthesis is shared among reverse transcriptases and DNA polymerases and involves successive conformational changes to their active site at each round of nucleotide addition (37). After the correct base-pairing with the incoming dNTP is formed, the fingers subdomain and RT-specific motif D rotate towards the RT active site and close around the dNTP (37).

During cryo-EM image processing, we observed lower local resolution estimates near the RT active site, suggesting the presence of alternative conformations. Focused classification revealed two configurations of the RT active site, herein termed open fingers state and closed fingers state (figs. S3, S7 and S8, and movie S1). In the open fingers state, the fingers subdomain and motif D rotate outwards from the active site, with weak density observed at the fingertips and for the base of the incoming dNTP (fig. S8A). Conversely, in the closed state, the fingers and motif D are rotated inwards and close around the incoming dNTP (fig. S8B). This closed conformation allows numerous contacts to form between the dNTP and the RT (fig. S8B). Observing these states in our dataset highlights the plasticity of the RT active site, necessary to achieve the processivity observed during DNA synthesis.

The wrist and CTD proceed the RT domain and lie at the C-terminus of ORF2p. The CTD harbors the essential cysteine-rich motif (6, 33), which adopts a C_2_HC zinc-finger (ZnF) fold (Fig. 1, G and J). We find that the CTD melts and interacts extensively with the target DNA, rather than with the substrate RNA as previously suggested (Fig. 1, F and 1G) (26, 38).

## ORF2p extensively remodels the target DNA to initiate TPRT

**Fig. 2:**
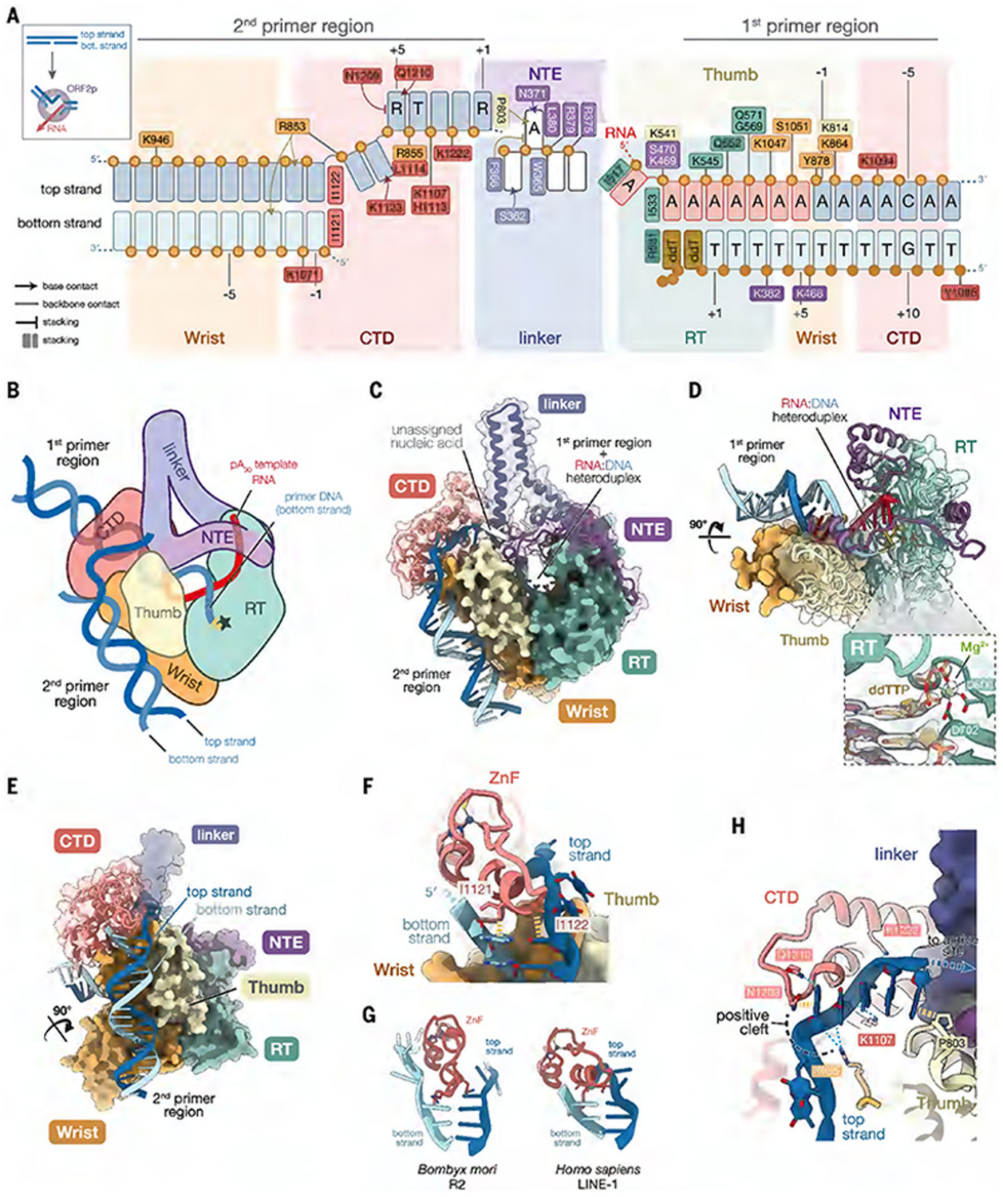
The target DNA is unzipped and broken across the domains of ORF2p. (**A**) Schematic of ORF2p interactions with the target DNA. Top and bottom strands are numbered relative to their respective cleavage sites. Inset shows a simplified schematic of target DNA remodeling accompanying TPRT complex formation. (**B**) Cartoon of the TPRT complex. Green star indicates RT active site. (**C**) Structure highlighting interactions with the two target DNA regions. (**D**) Structure surrounding the 1st primer region of the target DNA. Inset, view of ddTTP in the RT active site; cryo-EM density is shown as a transparent surface. (**E**) Structure surrounding the 2nd primer region of the target DNA. (**F**) CTD ZnF unzips the target DNA. Yellow dashed lines indicate stacking interactions. (**G**) Comparison of target DNA unzipping by *Bm*R2 ZnF (35). (**H**) Interactions with the melted top strand and ORF2p. Blue dashed lines indicate hydrogen bonding.

Our structure reveals the full engagement of ORF2p with the target DNA and the pA_30_ template RNA (Fig. 2A). ORF2p binding creates a sharp bend in the target DNA and roughly breaks it into two parts. We refer to these parts as the 1st primer region and the 2nd primer region henceforth (Fig. 2, A and B). The 1st primer region corresponds to the target DNA with the nicked bottom strand that primes first-strand cDNA synthesis of the template RNA (Fig. 2, B and C). Accordingly, this region harbors the template:primer heteroduplex formed by the pA_30_ template RNA and the nicked bottom strand primer (Fig 2, C and D). Similarly to previous structures (25, 26), the RT domain positions the heteroduplex within the RT active site, with varying contributions from the other domains of ORF2p (Fig. 2D).

The 2nd primer region, which has not been captured in previous structures, includes the region of the target DNA with the top strand anticipated to prime second-strand synthesis (Fig. 2, B and E). It is anchored at one end by a highly positive surface formed by the thumb, wrist and CTD domains (Fig. 2E).

We routinely observed varying lengths of the 2nd primer target DNA region in different 3D cryo-EM classes (fig. S9A and B and movie S2). Close inspection of the consensus cryo-EM map showed that the CTD unzipped the target DNA duplex by wedging the ZnF between the top and bottom strands. Two isoleucine residues extend from the ZnF helix to stack against the bases of the target DNA duplex (Fig. 2F). The melted top strand bends through a positively charged cleft formed by the CTD and wrist domain, then binds along the CTD towards the RT active site (Fig. 2H). In an almost ruler-like mechanism, the last 5 nt are sandwiched between Asn1209 and Pro803 from the CTD and thumb domain, respectively (Fig. 2H). These nucleotides adopt a nearly ideal B-form DNA geometry (fig. S9C). The phosphate backbone is buried into the surface of the CTD, while the base edges are exposed to the solvent.

A similar unzipping of the target DNA by a ZnF has been observed in the TPRT structure of *Bm*R2 (Fig. 2G) (35). While the role of the ZnF in unzipping the target DNA to initiate TPRT may be evolutionarily conserved, ORF2p may employ a mechanism distinct from that of *Bm*R2 for target DNA handling. Moreover, the extent of DNA remodeling differs from the target DNA bending observed in many DNA transposase and retroviral integrase systems (39) but is more akin to the nucleic acid rearrangements performed by CRISPR-Cas proteins (40).

## The top strand is nicked by the EN domain

The prevailing model for LINE-1 insertion consists of two steps: first-strand synthesis and second-strand synthesis (21). Under this model, ORF2p first nicks the bottom strand of the target DNA, then uses this nicked bottom strand to prime first-strand synthesis of the RNA template (28). It is thought that the top strand is nicked and primes second-strand synthesis after the first strand is synthesized. Our TPRT complex was stalled immediately after initiation and before completion of first-strand synthesis. We had, therefore, expected to see that only the bottom strand was nicked, and an unnicked top strand bridging the 1st and 2nd primer regions of the target DNA. However, our cryo-EM map showed that the top strand appeared to be nicked.

**Fig. 3:**
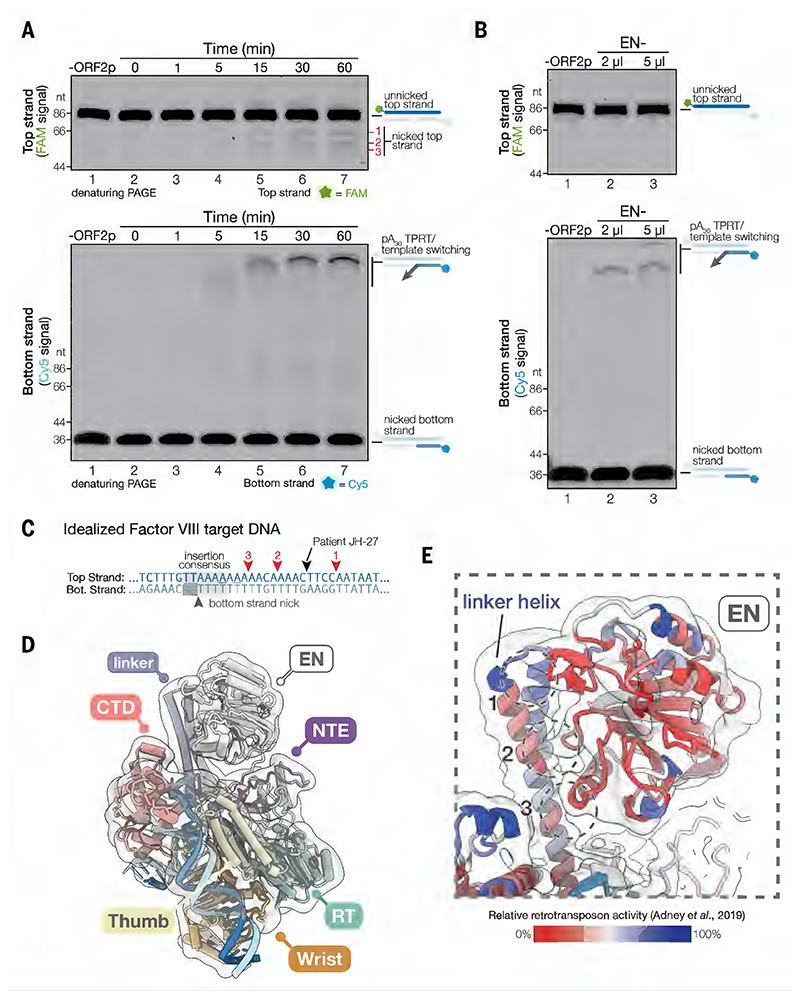
The top strand is nicked with reverse transcription. (**A**) Top strand nicking correlates with bottom strand TPRT. Denaturing gel of TPRT assay time course with doubly fluorescently labeled target DNA, visualized by FAM fluorescence to show top strand nicking (top) or by Cy5 fluorescence to show bottom strand TPRT products (bottom). (**B**) EN- (D145A) mutant blocks top strand nicking and reduces bottom strand TPRT. (**C**) Schematic of target DNA nicking. Red numbered triangles indicate the mapped cleavage sites of the top strand nicked products in (A). (**D**) Cryo-EM reconstruction with the EN domain resolved. Atomic model fit into the density is shown. Cryo-EM map was lowpass filtered to 8 Å. (**E**) Detailed view of EN-linker domain contacts. Retrotransposition efficiencies from trialanine scanning substitutions (50) are mapped onto the structure. Cryo-EM map was lowpass filtered to 5 Å.

To validate this observation, we performed TPRT assays using a target DNA substrate doubly labeled with FAM and Cy5 fluorophores on the top and bottom strands, respectively. This design allows us to simultaneously track first-strand synthesis, top strand nicking and second-strand synthesis. A time course of the TPRT assay showed that the top strand is indeed nicked, with cleavage primarily occurring at three positions (Fig. 3A, top). These nicked products accumulated over time (Fig. 3A, top) and tracked with the accumulation of the bottom strand TPRT product (Fig. 3A, bottom). Top strand nicking occurred in *cis* (fig. S10A), and the cleavage pattern was not affected by the sequence at the RNA 5' end (fig. S10B). An EN catalytic site mutant, D145A (19, 41) blocked top strand nicking and attenuated bottom strand TPRT products (Fig. 3B, EN-). Taken together, these results indicate that the EN domain nicks the top strand, and that top strand nicking does not license reverse transcription of the bottom strand.

We next sought to define the positions of the top strand cleavage sites by Sanger sequencing. Comparison to a DNA sequencing ladder showed that top strand nicking occurs upstream of the insertion consensus motif and is staggered +7, +11 and +19 nt relative to the bottom strand nick (Fig. 3C, arrows 3, 2, 1, respectively and fig. S10C). Staggered nicking of the top strand leads to the characteristic TSD flanking new retrotransposon insertions. The spacing observed in our assay is within the reported lengths of *in vivo* LINE-1 TSD events (23, 24). Furthermore, our TPRT structure would represent a mixture of states of the target DNA due to the heterogeneity in the cutting sites, together with the ability of ORF2p to unzip the target DNA. This would account for the ambiguity in the densities of the DNA bases in our consensus cryo-EM map (Fig. 2A). We did not observe nicking at the putative top strand cleavage site of patient JH-27, from whom this target DNA sequence was derived (Fig. 3C and fig. S10C) (27). This discrepancy was not caused by idealization of the target DNA because the same cutting sites were observed using a substrate with the native factor VIII sequence (Fig S10D). We also note that only one of the three top strand cleavage sites resembled the EN cleavage consensus motif (Fig. 3C, site 1), although previous studies have suggested that bottom and top strand cleavage events may have different sequence preferences (22). Alternatively, these differences may arise from the lack of host factors that could affect cleavage site choice. Overall, our TPRT structure suggests a new sequence of events for TPRT, in which top-strand nicking occurs with, or during, first-strand synthesis and may explain why most LINE-1 insertions are 5' truncated or 5' inverted (42–47).

## Conformational plasticity of the EN domain

We observed weak density near the long helices of the linker domain. We asked whether this corresponded to the EN domain in a more stable configuration, although it was too flexible to resolve in our consensus reconstruction. Through iterative rounds of focused classification and local refinement with Blush regularization (48), we resolved this domain to moderate resolutions (4.0–6.5 Å) (Fig. 3D and fig. S11). Docking a crystal structure of the EN domain (49) into the resulting cryo-EM map shows three main contacts with the long helix of the linker domain (Fig. 3E, circles 1, 2 and 3). Alanine scanning substitutions (50) at either the EN or linker contact sites decreases retrotransposition activity of LINE-1, whereas substitutions of the neighboring linker helix do not (Fig. 3E). This suggests that these interactions are important for LINE-1 retrotransposition.

While the flexibility of the EN domain and the melting of the target DNA were unexpected findings, they may explain the varying TSD lengths flanking new retrotransposon insertions. These TSDs arise from the staggered cleavage of the target DNA top strand relative to the bottom strand nick, and are variable in length, but usually < 20 nt (23, 24). Modeling a nicked target DNA into our EN-resolved map places the EN domain ~ 20 nt away from the bottom strand nick (fig. S11G) and may represent the ‘default’ configuration of the EN domain for top strand nicking. Target DNA unzipping and ORF2p sliding would draw the bottom strand nick towards the EN domain, resulting in TSDs shorter than 20 nts (see below Fig. 5, step 4). This mechanism, coupled with the flexibility of the EN domain, may allow ORF2p to sample the target DNA for an ideal top strand cleavage site, before committing to first-strand synthesis.

## Cellular factors facilitate nucleic acid binding

Many of the interactions between ORF2p, and the target DNA and template RNA are made through the phosphate backbone and are not sequence specific. This was unexpected because LINE-1 retrotransposition demonstrates at least two nucleic acid specificities: (i) insertion at EN consensus cleavage sites (19, 22, 51) and (ii) reverse transcription of its own mRNA which requires the poly(A) tract (52). The *in vitro* top strand cleavage sites did not match patient JH-27 (27), contrary to our expectations. Numerous cellular proteins are known to associate with the LINE-1 RNP and some are essential for retrotransposition (53–58), raising the possibility that these proteins facilitate the nucleic acid specificity of LINE-1 retrotransposition.

**Fig. 4:**
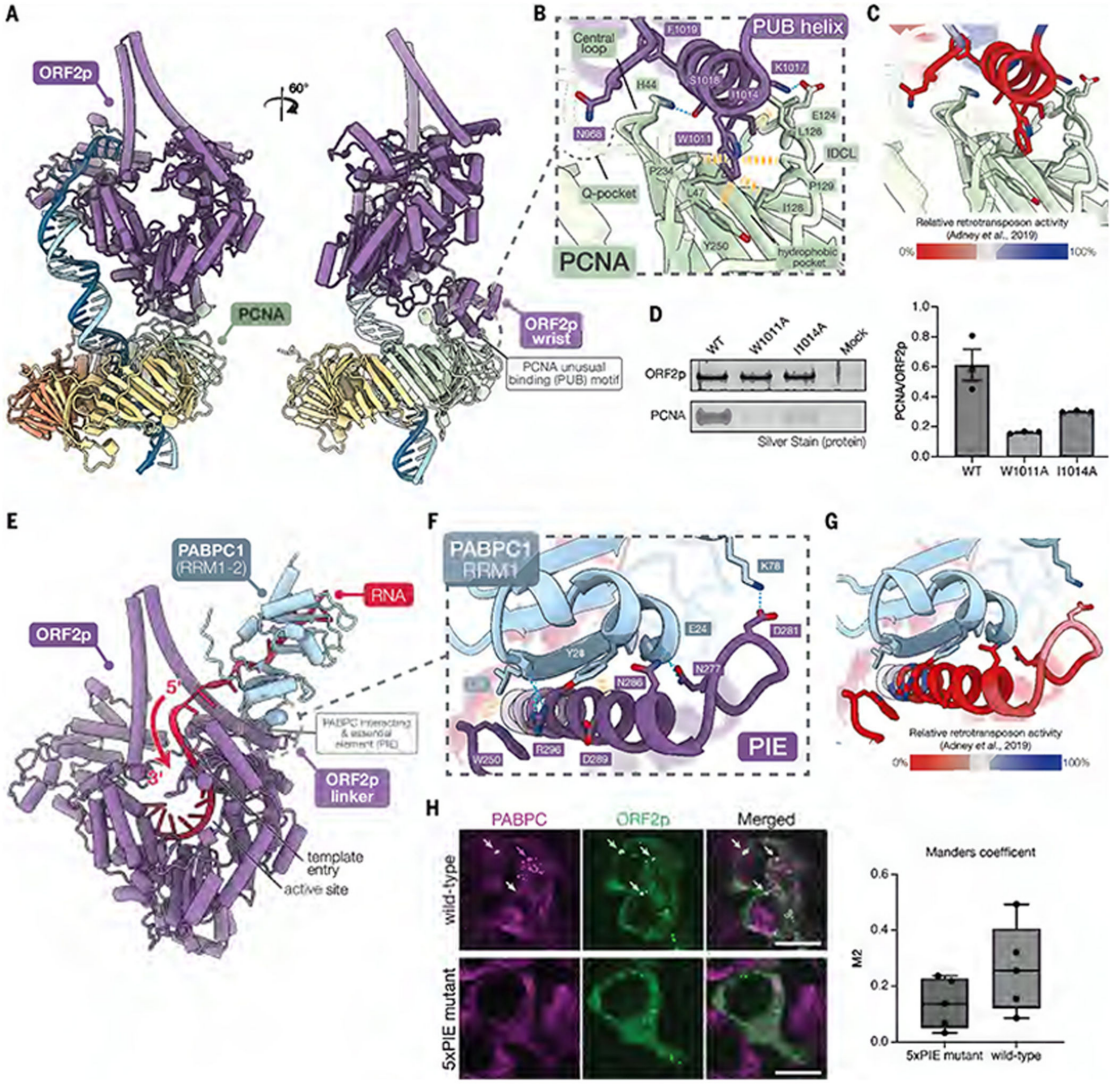
Cellular factors facilitate nucleic acid binding. (**A**) AlphaFold 3 prediction of the ORF2p-PCNA complex. (**B**) Detailed view of the predicted interaction between the PUB motif and PCNA. IDCL, interdomain connector loop. (**C**) Effect of PUB trialanine substitutions on retrotransposition efficiency. The same view as in (B) is shown, with retrotransposition efficiencies from trialanine scanning substitutions (50) mapped onto the structure. (**D**) Silver-stained SDS-PAGE from ORF2p pulldown experiments with PUB site mutants (left panel) and quantification of the pulldown experiments (right panel). The experiments were performed in triplicate (n = 3). Values represent PCNA band intensity normalized to ORF2p band intensity. Error bars represent standard error of the mean (SEM). (**E**) AlphaFold 3 prediction of the ORF2p-PABPC1 complex. RRM1-2, RNA recognition motif 1 and 2. (**F**) Detailed view of the interactions between the PIE region of ORF2p and PABPC1. RRM, RNA recognition motif. (**G**) Effect of PIE trialanine substitutions on retrotransposition efficiency. The same view as in (F) is shown, with retrotransposition efficiencies from trialanine scanning substitutions (50) mapped onto the structure. (**H**) Immunofluorescence staining of ORF2p (wild-type or 5xPIE mutant; green) and PABPC (magenta). Arrows indicate examples of ORF2p cytoplasmic puncta co-localized with PABPC, which were not observed in the ORF2p 5xPIE mutant. 5xPIE mutant, M272A, N277A, D281A, N286A and R296A. Scale bar: 10 µm. Quantification of Manders coefficients (n = 10, right).

To assess this, we used AlphaFold3 to predict the structures of ORF2p with a non-redundant list of known interactors (Data S1) (54, 55, 59). High-confidence interactions with proliferating cell nuclear antigen (PCNA) and cytoplasmic poly(A)-binding protein 1 (PABPC1) stood out among the predicted structures (Fig. 4 and fig. S12 and S13).

### PCNA binds a novel site on ORF2p

Human PCNA, also known as the sliding clamp, is a homotrimeric DNA-processivity factor and is essential to DNA replication and repair (60). PCNA co-purifies with the LINE-1 RNP and was proposed to interact with a canonical PCNA-interacting peptide (PIP) box motif in the NTE domain of ORF2p (residues 407–415) (25, 54).

However, AlphaFold3 predicted that PCNA interacts with the wrist domain of ORF2p, instead (Fig. 4A and fig. S12, A–C). The predicted ORF2p-PCNA interaction differs substantially from canonical PIP box-PCNA interactions (Fig. 4B, fig. S12D and Supplementary text). Here we find that a helix from the ORF2p wrist domain lies along the PCNA hydrophobic pocket, against the interdomain connector loop (IDCL) (Fig. 4B), to bury Trp1011 and Ile1014. Additionally, Asn968 from a neighboring loop of the wrist domain extends into the PCNA Q-pocket. This mimics the typical glutamine-Q-pocket interaction typically observed in canonical PIP box-PCNA interactions (Fig. 4B). We term these regions of the ORF2p wrist domain the PCNA unusual binding (PUB) motif.

Four pieces of evidence support the prediction: (i) the predicted PUB-PCNA interaction places PCNA directly in line with the target DNA in our cryo-EM structure (Fig. 4A), (ii) trialanine scanning substitutions (50) at residues in the PUB motif severely disrupt retrotransposition (Fig. 4C), (iii) ORF2p W1011A and I1014A PUB mutants are defective in binding to PCNA in ORF2p pulldown experiments (Fig. 4D) and (iv) PUB motif residues predicted to interact with PCNA are highly conserved across LINE-1 elements from divergent species (fig. S12E, starred residues).

### PABPC1 binds near the template RNA entry channel of ORF2p

PABPC1 belongs to a family of highly abundant cytoplasmic poly(A)-binding proteins, which regulate numerous facets of mRNA biology, including translation initiation, deadenylation and mRNA decay (61–68). PABPC1 binds RNA through four consecutive RNA-recognition motif (RRM) domains, where RRM1 and RRM2 mainly confer adenosine-binding specificity and affinity (69–72). Previous studies have shown that PABPC1 is a component of the LINE-1 RNP and is required for efficient retrotransposition by promoting cytoplasmic RNP formation (53–55). Yet, it is unclear if PABPC1 directly interacts with LINE-1 components or if its association is simply explained by binding to the LINE-1 mRNA poly(A) tail (55).

Our structure predictions show that the RRM1 of PABPC1 directly binds the linker domain of ORF2p (residues 272–297) via numerous sidechain-backbone and sidechain-sidechain interactions (Fig. 4, E and F). We name this region of the linker domain the PABPC interacting and essential element (PIE). The RRM1 and RRM2 domains of PABPC1 bind the poly(A) RNA directionally, in a 3'-to-5' polarity (73). The PIE-PABPC1 interaction positions RRM1 near the template RNA entry channel of ORF2p. This positioning would allow PABPC1-bound RNA to enter the RT active site in the proper orientation necessary to pair with a target DNA primer strand.

Alanine substitutions of PIE residues severely disrupt activity, suggesting that the ORF2p interaction with PABPC1 is critical for retrotransposition (Fig. 4G). PIE residues are highly conserved across divergent species, particularly towards the PIE C-terminal region where many of the interactions with RRM1 occur (fig. S13D, residues 285–297). When wild-type ORF2p was overexpressed in HEK293T cells, it colocalized with PABPC in cytoplasmic puncta (Fig. 4H, arrows and fig. S13E, arrows). However, overexpression of ORF2p carrying PIE site mutation disrupted this colocalization as PABPC did not localize to ORF2p puncta (Fig. 4H and fig. S13E). Taken together, these observations suggest that PABPC1 binding is a fundamental aspect of LINE-1 retrotransposition and may possibly help mediate LINE-1 *cis*-preference (see Discussion).

## Discussion

**Fig. 5:**
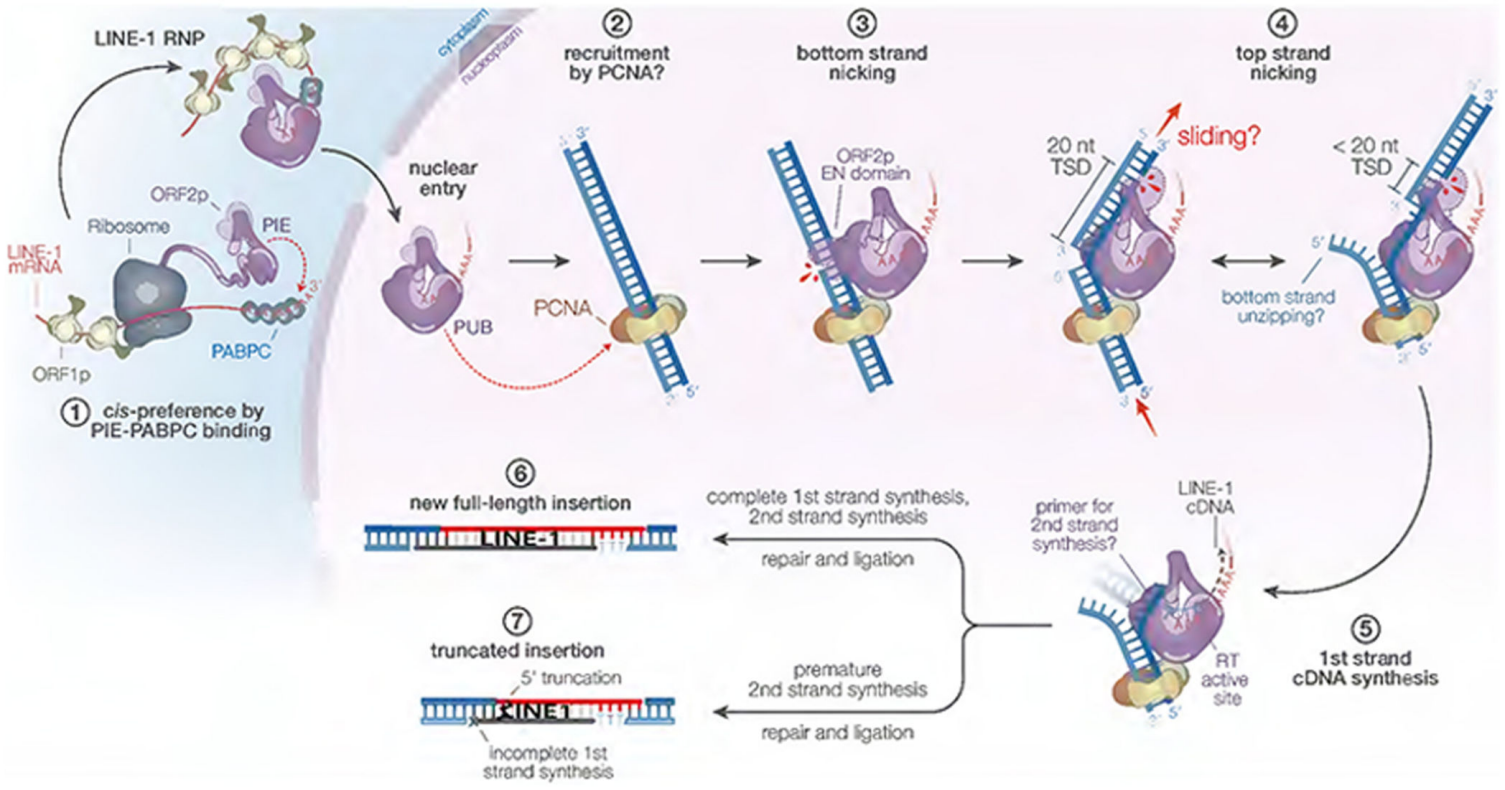
Model for TPRT and LINE-1 retrotransposition. **1**, Co-translational ORF2p PIE-PABPC binding establishes *cis*-preference. **2**, After RNP formation and nuclear entry, PCNA recruits the LINE-1 RNP to a target DNA with the appropriate architecture for retrotransposition. **3**, EN domain nicks the bottom strand at a site resembling the EN cleavage consensus motif. **4**, Sliding and unzipping of the target DNA allows the EN domain to nick the top strand at a suitable site and explains the observed distribution of target site duplication (TSD) lengths. The timing of top strand nicking is unclear. **5**, First-strand cDNA synthesis initiates after the bottom strand is passed to the RT active site and anneals with the poly(A) tail. **6**, Complete first-strand synthesis followed by template jumping to the exposed top strand initiates second-strand synthesis and results in a new full-length insertion. **7**, Premature second-strand synthesis before first-strand has completed would lead to a new 5' truncated insertion.

Here we present the structure a human LINE-1 RNP stalled at TPRT, giving a molecular view into the process that has written nearly 30% of our genomes. Our work not only provides key insights into the mechanism of TPRT, but also into other areas of LINE-1 retrotransposition. This allows us to propose a retrotransposon model that accounts for several previously unclear aspects and is summarized in Figure 5.

The LINE-1 machinery preferentially acts upon its own mRNA, a characteristic known as *cis*-preference (74). While *cis*-preference requires a poly(A) tail and is thought to occur co-translationally (52, 75), how ORF2p selects its own mRNA has been unclear. PABPC1 binding to the PIE motif at the N-terminus of nascent ORF2p (Fig. 1A) would establish *cis*-preference co-translationally and may facilitate RNP formation by positioning the RNA for co-folding with ORF2p (Fig. 5, step 1). This is consistent with previous studies showing that PABPC1 depletion causes a defect in LINE-1 RNP formation (53). Additionally, PABPCs multimerize across the poly(A) tail (76, 77), limiting access to all but the most distal RRM1 domain near the mRNA 3' end. ORF2p-RRM1 binding would then position ORF2p near the mRNA 3' end (Fig. 5, step 1 red arrow), and may protect the LINE-1 mRNA from deadenylation, similarly to the LARP1-PABPC complex (78). Protecting against deadenylation may ensure that the LINE-1 mRNA maintains a long poly(A) tract - a feature that coincides with retrotransposition potential (52, 79).

Although the physiological requirements for LINE-1 TPRT are still unclear, several lines of evidence indicate that the DNA architecture is critical to target DNA selection. First, bottom strand nicking and TPRT is nearly undetectable on double stranded target DNA substrates (Fig. 1E, lane 2). Second, retrotransposition appears linked to DNA replication (55, 80–82). Third, DNA substrates that mimic replication intermediates strongly stimulate EN bottom strand nicking (26). It is important to note that our TPRT complex assembly approach bypasses bottom strand nicking of TPRT. Therefore, we lack structural insights into the early stages of ORF2p engagement with the target DNA and any potential DNA architectural requirements critical for this process.

It is possible that ORF2p exploits PCNA to find a target DNA with a suitable architecture (Fig. 5, step 2). The predicted interaction between ORF2p and PCNA is far more extensive than the typical PIP box-PCNA interaction with canonical PCNA binding partners. This may allow ORF2p to outcompete or displace these factors for PCNA binding. It is not so surprising that ORF2p uses host factors for retrotransposition by binding to conserved binding sites. This would prevent the host from escaping retrotransposition by mutating these sites (83).

The nucleic acid architecture observed in our TPRT structure provides broader insights into the pathway of LINE-1 retrotransposition. While it is assumed that ORF2p nicks the top strand to initiate second-strand synthesis, the exact mechanism has been unclear. Our findings show that ORF2p not only nicks the top strand but also rearranges the target DNA into a state that appears primed for second-strand synthesis—all before or during first-strand synthesis (Fig. 5, step 5). This contrasts a recently proposed model in which ORF2p remodels a template RNA duplex and does not nick the top strand, instead relying on replication intermediates to generate a primer for second-strand synthesis (26).

While these models are not mutually exclusive, our structure suggests a pathway that explains the variability in TSD length (see above) and may account for the structural sequence variations observed at LINE-1 insertions events, particularly 5' truncations (42, 43, 45, 47). Nicking of the top strand provides the primer needed for second-strand synthesis before the completion of first-strand synthesis (Fig. 5, step 5). Initiating second-strand synthesis after completing first-strand synthesis would result in a new full-length LINE-1 insertion (Fig. 5, step 6). Alternatively, a premature transition to second-strand synthesis would result in 5' truncated insertions (Fig. 5, step 7).

## Materials and Methods

### ORF2p purification

A codon optimized human ORF2p sequence (a gift from D. Rio) was cloned into the pACEBac1 transfer vector containing an N-terminal 8xHis-TwinStrep-MBP-SUMO* tag. Baculoviruses were generated using the Bac-to-Bac Baculovirus expression system (Invitrogen) and EmBacY cells (Geneva Biotech) (83). For expression, 1 L of *Trichoplusia ni* High Five at a density of 1.0 x10^6^ cells/ml was infected with 10 ml of high titer baculovirus stock. Infected cells were grown for 72 h at 27 °C, harvested by centrifugation, snap frozen in liquid nitrogen and stored at -70 °C until lysis.

For lysis, cell pellets were thawed and resuspended in hypotonic lysis buffer (20 mM HEPES-NaOH pH 8.0, 2 mM MgCl_2_, 10 µM ZnCl_2_, 0.2 mM EGTA, 10 % glycerol, 0.1 % IGEPAL CA-630, 1 mM DTT, 1 mM PMSF, 1 cOmplete Protease Inhibitor Cocktail tablet/50 ml (Roche)). Extracts were prepared by three freeze-thaw cycles and clarified by centrifugation after adjusting the salt concentration to 300 mM with 5 M NaCl. Clarified extracts was adjusted to 150 mM NaCl by dilution followed by snap freezing in liquid nitrogen and storage at -70 °C until purification.

ORF2p was purified from extracts by a two-step procedure. First, extracts were thawed, supplemented with 15 µl/ml of BioLock (IBA LifeSciences) and 1/10 volume 4 M (NH_4_)_2_SO_4_, and then passed through a 0.22 µm syringe filter. Filtrates were applied on to 5 ml of pre-equilibrated Strep-Tactin XT Sepharose (Cytiva) by gravity flow. The resin was washed three times with 10 column volumes (CVs) of O2 buffer (25 mM HEPES-NaOH pH 8.0, 400 mM (NH_4_)_2_SO_4_, 1 mM MgCl_2_, 10 µM ZnCl_2_, 1 mM DTT, 1 mM PMSF), once with 5 CVs of A2 buffer (O2 buffer supplemented to 500 mM with L-arginine HCl), and finally eluted 3 times in batch with 1 CV of E2 buffer (O2 buffer supplemented to 500 mM L-arginine HCl and 50 mM biotin).

Next, the salt concentration was adjusted to 100 mM by dilution before application onto a 1 ml HiTrap SP HP cation exchange column (Cytiva) pre-equilibrated in IEX A buffer (25 mM HEPES-NaOH pH 8.0, 100 mM (NH_4_)_2_SO_4_, 1 mM MgCl_2_, 10 µM ZnCl_2_, 1 mM DTT, 1 mM PMSF) and then eluted with a linear gradient to 1.2 M (NH_4_)_2_SO_4_. Fractions with the highest specific activity (typically a single 200 µl fraction) were supplemented to 10 % with glycerol, aliquoted, snap frozen in liquid nitrogen and stored at -70 °C until use in biochemical assays. For structural determination, fractions were immediately used for complex formation and cryo-EM sample preparation. Protein concentration was determined was by densitometry.

### PCNA purification

Human PCNA with an N-terminal polyhistidine SUMO tag expression plasmid was transformed into BL21(DE3) *E. coli* strain. Two liters of cells were cultured at 37 °C in 2xTY medium to an OD of 0.6, then induced with 0.8 mM IPTG followed by a 3 h incubation at 37 °C. Cells were harvested, resuspended in IMAC A buffer (50 mM Tris-HCl pH 8.0, 300 mM NaCl, 0.01 % IGEPAL CA-630, 30 mM imidazole, 0.5 mM DTT, 1 mM PMSF), sonicated and clarified by centrifugation for 30 min at 4 °C and 25,000 g. The lysate was filtered and applied to a 5 ml HisTrap HP column (Cytiva). The column was washed with IMAC A before elution with a linear gradient to IMAC A supplemented with imidazole to 500 mM. The tag was cleaved overnight with SUMO protease (LifeSensors).

The salt concentration was adjusted to 150 mM by dilution before application onto a 1 ml HiTrap Q HP anion exchange column (Cytiva) pre-equilibrated in IEX A buffer (25 mM Tris-HCl pH 7.4, 150 mM NaCl, 10 % glycerol, 0.5 mM DTT, 1 mM PMSF) and eluted with a linear gradient to 1.0 M NaCl. The eluate was supplemented with imidazole to 35 mM and applied to 3.0 ml of Ni Sepharose HP resin (Cytiva) to remove the tag and uncleaved PCNA.

PCNA was further purified using a HiLoad Superdex 200 size exclusion column (Cytiva) in SEC buffer (25 mM Tris-HCl pH 7.4, 300 mM NaCl, 10 % glycerol, 0.5 mM DTT, 1 mM PMSF). Peak fractions were pooled, concentrated to 70 µM, aliquoted, flash-frozen in liquid nitrogen and stored at -80 °C.

### ORF2p pulldown with PCNA

Insect cells were infected with ORF2p mutants and lysed as described above. Extracts were thawed, supplemented with 15 µl/ml of BioLock (IBA LifeSciences), 1/10 volume 4 M (NH_4_)_2_SO_4_, and then passed through a 0.22 µm syringe filter. 1.5 ml of filtrates were incubated in batch with 200 µl of MagStrep Strep-Tactin beads (5 % suspension, IBA LifeSciences) for 30 min at 4 °C. The resin was washed three times with 1.0 ml of O2 buffer, then washed twice with binding buffer (25 mM HEPES-NaOH pH 8.0, 150 mM KOAc, 1.5 mM Mg(OAc)_2_, 10 µM ZnCl_2_, 1 mM DTT, 0.5 mM PMSF). The resin was incubated in batch with 0.25 µM PCNA in 100 µl binding buffer for 30 min at 4 °C. The resin was then washed three times with 0.8 ml binding buffer and eluted in batch at 22 °C with 30 µL binding buffer supplemented with 50 mM biotin. Fractions were analyzed by silver stained SDS-PAGE and quantified using Image J (version 2.14.0). The experiments were performed in three independent technical replicates.

### Preparation of RNA substrates

For the *Alu*Ya5 RNA substrate, a 5 ml *in vitro* transcription (IVT) reaction was prepared from a *NheI*-linearized plasmid template of the *Alu* RNA including a 55 nt poly(A) sequence. IVT reactions comprised of 4 mM of each ATP, CTP, GTP and UTP, 40 mM Tris HCL pH 8.0, 30 mM MgCl_2_, 2 mM spermidine, 10 mM DTT, 200 µg/ml DNA template, and 15 µg/ml of homemade T7 RNA polymerase. The reaction was incubated overnight at 37 °C. The magnesium pyrophosphate precipitate was pelleted and removed by centrifugation, followed by the addition of 50 U of RQ1 RNase-Free DNase (Promega), and incubation at 37 °C for 1 hr. The RNA was extracted twice with acidic phenol:chloroform:isoamyl alcohol, supplemented with 1/10 volume of 3 M NaOAc pH 5.2, then precipitated by the addition of 2.5 volumes of 100 % ethanol followed by overnight incubation at -20 °C. The RNA pellet was washed twice with 70 % ethanol, dried, resuspended in TE (10 mM Tris-HCl pH 8.0, 10 mM EDTA), aliquoted and stored at -70 °C until use.

30 nt poly(A) RNA oligonucleotides were synthesized by IDT. The sequences of the RNA substrates used in this study are listed in table S2.

### Preparation of DNA substrates

Target DNA sequences were derived from *de novo* LINE-1 insertions into exon 14 of the human factor VIII gene (27). This target DNA sequence was idealized to extend the region of complementarity with the poly(A) tail. DNA oligonucleotides were synthesized by IDT and resuspended in 1X Annealing buffer (10 mM HEPES-NaOH pH 8.0, 60 mM KCl). Where necessary, DNA oligonucleotides were purified by preparative denaturing PAGE.

Equimolar ratios of target DNA strands were mixed at 25 µM final concentration, then annealed by heating to 98 °C for 5 min in a heat block and allowed to cool overnight to ambient temperature. Sequences of DNA oligonucleotides used in this study are listed in table S2.

### *In vitro* target-primed reverse transcription (TPRT) assays

TPRT reactions were carried out in 20 µl volumes and typically comprised of 50 nM labeled target DNA, 1 µM RNA in 25 mM HEPES-NaOH pH 8.0, 500 mM KOAc, 1.5 mM Mg(OAc)_2_, 10 µM Zn(OAc)_2_, 1 mM DTT and 25 µM of each dNTP. Reactions were initiated by the addition of 1–5 µl of ORF2p protein (to a final concentration of 7. 5–37.5 nM) and incubated at 37 °C for 1 h. Reactions were stopped by adding 1 µl of 20x STOP mix I (1 mg/ml RNase A, 120 mM EDTA). For experiments in Fig. 3, reactions were stopped by adding 1 µl of 20x STOP mix II (2 % SDS, 200 mM EDTA) and 1 µg RNase A per reaction. RNA was digested for 20 min at room temperature and incubated at 37 °C for 30 min after adding 10 µg proteinase K per reaction.

One volume of 2x loading buffer (95 % deionized formamide, 0.02 % SDS, 1 mM EDTA) was added to each sample, followed by the addition of 1/20 volume of 100 mM NaOH, before boiling for 5 min. Reactions were resolved by 12 % denaturing PAGE run at 10 W for 10–25 min. Gels were visualized on a Typhoon Imager (Cytiva).

Target DNA substrates used in TPRT assays typically had a 5' fluorescein modification (6-FAM) on the bottom strand. For experiments in Fig. 3, target DNA substrates were modified with 5' fluorescein on the top strand and 5' Cy5 on the bottom strand.

### Mapping top strand cleavage sites

To map the top strand cleavage sites in Fig. 3A, TPRT products were compared to a top strand sequencing ladder generated with Therminator (NEB) and dideoxynucleoside triphosphates (ddNTPs, Roche). Briefly, sequencing ladder reactions comprised of 20 nM 5' 6-FAM labeled top strand primer, 10 nM bottom strand template, 1x ThermoPol buffer (NEB), 0.02 U/µl Therminator DNA polymerase (NEB), 0.02 U/µl thermostable inorganic pyrophosphatase (NEB), 100 µM dNTPs and one ddNTP per reaction (5 µM ddATP; 5 µM ddTTP; 2.5 µM ddCTP; or 2.5 µM ddGTP) in a volume of 20 µl. Reactions were incubated in a thermocycler at 94 °C for 10 min, followed by 25 cycles of 94 °C for 30 s, 55 °C for 30 s and 72 °C for 1 min, followed by one cycle at 72 °C for 1 min.

Reactions were brought up to 200 µl with standard TE buffer, extracted once with phenol:chloroform:isoamyl alcohol and supplemented with 20 µl of 3M NaOAc and 20 µl of 20 mg/ml glycogen before precipitation with 2.5 volumes of cold 100 % ethanol at -20 °C overnight. Samples were pelleted by centrifugation. Pellets were washed once with 70 % cold ethanol, pelleted again, dried and resuspended in 15 µl of 1:1:8 water to 100 mM NaOH to 2x loading buffer. After boiling for 5 min, 5 µl of each sample were resolved on a 12 % denaturing PAGE 40 cm x 0.4 mm sequencing gel run at 25 W for 2–3 h. Gels were visualized on a Typhoon Imager (Cytiva).

### Immunofluorescence staining and quantification

HEK293T cells (ATCC, Cat# CRL-3216, RRID:CVCL_0063) were cultured in DMEM with GlutaMax (ThermoFisher), supplemented with 10 % FBS, 100 μg/ml streptomycin, and 100 unit/ml penicillin. Cells were seeded in 6-well plates on coverslips coated with 0.1% poly-L-lysine (Sigma-Aldrich, Cat. P1399) in PBS to 70-80 % confluency and then transfected with 4 µg of plasmid DNA of either wild-type ORF2p or 5xPIE ORF2p mutant using Lipofectamine™ 2000 (ThermoFisher Cat. 11668027). Wild-type and mutant ORF2p expression constructs carried a C-terminal Strep tag. After 24 hours, the transfected cells were washed with PBS twice, fixed using cold 4% paraformaldehyde in PBS for 20 min and washed with PBS again twice. Cells were permeabilized by incubation with 0.5 % v/v Triton X-100 in PBS for 2 mins at room temperature and washed three times with PBS. Following incubation with blocking buffer (5 % BSA in PBS) for 30 mins at room temperature, the cells were incubated with anti-Strep (Abcam ab252885, 1:1000) and anti-PABP (Abcam ab312314, 1:250) in blocking buffer overnight at 4 ºC. The cells were washed three times with PBS and incubated with goat anti-rat Alexa-Fluor 488 (Abcam, ab150165, 1:1000) and goat anti-rabbit Alexa-Fluor 647 (Life Technologies A21245, lot 1752070, 1:1000) in PBST for 1 h in the dark at room temperature. After washing three times with PBS, the coverslips were mounted on a glass slide using Vectashield Plus antifade mounting medium with DAPI (Vector Laboratories, H-2000), sealed and stored at 4 ºC until imaging.

The fixed cells were imaged on a Zeiss 780 confocal microscope operated at room temperature. The fluorescence signals were obtained using the 488 and 633 nm lasers. Image stacks were taken with a 63x/0.4NA oil-immersion objective (pixel size = 0.2636 μm) using a GaAsP detector every 0.3977 μm. The Manders' overlap coefficient were computed using a custom-made ImageJ macro that, for each channel, segmented the blob like regions using a difference of Gaussian filters with respective sigma 1 and 4 pixels and a threshold set to the mean plus twice the standard deviation of the filtered image. This provided 3D binary masks for each channel from which the coefficient was computed as the sum of the intensity of a channel in the intersection of the masks normalized by the sum of intensity in the mask associated to this channel. Boxplots for the coefficients were plotted using Prism 10 (GraphPad).

### ORF2p TPRT complex formation for cryo-EM

Our initial attempts to prepare high-quality TPRT complexes for cryo-EM were unsuccessful. ORF2p readily precipitated over the course of complex formation and a substantial fraction of our protein preparations were still inactive, despite the high-specific activity. To overcome these challenges, we purified active TPRT complexes away from inactive ORF2p using a biotinylated pre-nicked target DNA (fig. S2A). This purification strategy has been successfully used for other challenging nucleoprotein complexes (35, 84).

For assembly of the ORF2p TPRT complex, 50 µl (~45 nM) of freshly purified high-specific activity ORF2p was supplemented with 90 nM of a pre-nicked target DNA sequence and 1 µM 30 nt poly(A) RNA, and dialyzed against dialysis buffer (25 mM HEPES-NaOH pH 8.0, 500 mM KOAc, 1.5 mM Mg(OAc)_2_, 10 µM Zn(OAc)_2_, 1 mM DTT, 0.5 mM PMSF) at 4 °C overnight. The target DNA contained a 5' desthiobiotin-TEG modification on the top strand, and 5' fluorescein on the bottom strand. The sample was supplemented with 100 µM of 2',3'-dideoxythymidine triphosphate (ddTTP) and incubated at 37 °C for 30 min before binding to streptavidin mag sepharose (Cytiva) for 1 h at 4 °C. The resin was washed twice with dialysis buffer supplemented with 25 µM ddTTP and eluted for 30 min at 37 °C with dialysis buffer supplemented with 25 µM ddTTP and 50 mM biotin. Eluted complexes were immediately used for vitrification.

### Cryo-EM grid preparation and data collection

Vitrification was performed using a Vitrobot Mark IV (ThermoFisher Scientific) maintained at 4 °C and 100 % humidity. 3 µl of ORF2p TPRT complex was applied onto freshly glow-discharged (1 s at 40 mA) Quantifoil R 1.2/1.3 Au 300 grid, pre-coated with a layer of graphene oxide by following a published procedure (85, 86). After a 30 s incubation, the grid was blotted for 3 s with a blot force of -10 and subsequently plunged into liquid ethane.

Data collection was performed on a Titan Krios G4 cryo-transmission electron microscope operated at 300 kV with fringe-free imaging and equipped with a C-FEG, Selectris X energy-filter, and a Falcon 4i direct electron detector (ThermoFisher Scientific). 25,374 movies were automatically collected using EPU (ThermoFisher Scientific) in counting mode with a pixel size of 0.955 Å over a defocus range of -0.8 µm to -2.2 µm. We used a flux of 9.45 e^-^/px/s and an exposure time of 5.85 s, yielding a total fluence of 59.22 e^-^/Å^2^. Each movie was fractionated into 50 movie frames. An energy filter slit width of 10 eV was used.

### Cryo-EM data processing

#### Processing strategy for the consensus reconstruction

Data were processed using RELION-5.0 unless otherwise indicated (fig. S3). 25,374 movies were gain-corrected, dose-weighted and motion-corrected using the RELION implementation of MotionCor2. CTF parameters were estimated using CTFFIND-4.1 (87). After manual curation, 25,018 micrographs were split by estimated defocus parameters, resulting in three groups with 9,252 (defocus ≤ -2.2 µm), 8,473 (-2.2 µm < defocus < -1.5 µm), and 7,293 (defocus ≥ -1.5 µm) micrographs, respectively. Particle picking was carried out using Topaz with the general model (88). Particles were extracted from each defocus group using varying figure of merit values (-0.5, -1.0, and -2.0, respectively) at a pixel size of 4.46 Å/px and box size of 60^2^ pixels, yielding 4,568,277 particles.

The particles were first filtered by 3D classification, then filtered by 2D classification without alignment, yielding a subset of 1,371,912 particles. Particles were reextracted at an unbinned pixel size of 0.955 Å/px and box size of 280^2^ pixels, and then refined to 2.72 Å resolution. We noticed that this subset contained a substantial number of particles with high defocus values; therefore, we reextracted the particles with an increased box size of 380^2^ pixels to capture more signal delocalized by the CTF.

The reextracted particles were classified into six 3D classes without alignment and with a regularization parameter *T* of 24. We combined five classes with well-defined, high-resolution features, yielding a subset of 680,273 particles, which we then refined to 2.62 Å resolution. Iterative rounds of CTF refinement (beam tilt, trefoil, and 4th order aberrations; anisotropic magnification; per-particle defocus, and per-micrograph astigmatism) (89), 3D refinement and Bayesian polishing (90) culminated in a consensus reconstruction at 2.27 Å resolution.

#### Processing strategy for the open fingers and closed fingers reconstructions

We noticed characteristics of varying occupancies in the density around the active site of the consensus reconstruction. Therefore, the particles were imported to CryoSPARC v4.5.3 for non-uniform refinement and 3D classification with mask surrounding the active site (fig. S3) (91, 92). To resolve the open fingers state, particles from the non-uniform refinement were subject to focused 3D classification into four classes, initialized by PCA, at a target resolution of 3 Å. A subset of 183,579 particles showed well defined density for the fingers in the open configuration.

To resolve the closed fingers state, particles from the non-uniform refinement were subject to focused 3D classification as above, except with a class similarity parameter of 0.1, and an input initialization mode. This produced one class with well-defined density for the fingers in the closed configuration and strong occupancy of the ddTTP in the active site, corresponding to a subset of 185,228 particles.

Each subset was prepared for downstream processing in RELION using an in-house Python script which implements PyEM (93) and Starparser (94). This yields a subset from the original RELION .star file, corresponding to the particles classified in CryoSPARC. The resulting .star files were imported into RELION and refined to 2.45 Å resolution and 2.50 Å resolution for the open fingers state and closed fingers state, respectively.

#### Processing strategy for EN-resolved reconstruction

During the above analysis, we noticed weak density in some of the classes that could correspond to the EN domain. Therefore, we imported the original 1,371,912 particle subset from RELION, into CryoSPARC for non-uniform refinement and focused 3D classification with a generous mask near the linker domain (fig. S11A). The 3D classification was initialized using 4 volumes and a filter resolution of 16 Å. One class, consisting of 443,253 particles, displayed strong EN density and was reimported into RELION using the strategy described above. Particles were reextracted at bin 2 to speed up calculations, then subject to focused 3D classification without alignment and with a regularization parameter *T* of 250. A class with 121,941 particles with the best resolved EN density was subsequently refined to 3.18 Å. CTF refinement (beam tilt, trefoil, and 4th order aberrations; anisotropic magnification; per-particle defocus, and per-micrograph astigmatism) followed by Bayesian polishing, and 3D refinement with Blush regularization (48) yielded a final 3.1 Å reconstruction. Multi-body refinement was used to characterize the flexibility of the EN domain (fig. S11F) (95). The position of the EN domain was “fixed” during multi-body refinement by setting the widths of the rotational and translational priors set to zero (96).

For all maps, resolutions are reported using the gold-standard Fourier shell correlation (FSC) = 0.143 criterion (figs. S4A, S7, A and B, and S11B). B-factors were determined by RELION or from a user-defined value (table S1). Local resolutions were calculated in RELION (figs. S4D, S7, G and H, and S11E). Directional FSC plots and sphericity values, calculated using a 3D-FSC webserver (figs. S4B, S7, C and D, and S11C) (https://3dfsc.salk.edu/) (97). Particle orientation plots (Euler angles) were calculated using a Python script (figs. S4C, S7, E and F, and S11D) (https://github.com/Guillawme/angdist).

### Model building, refinement and AlphaFold3 prediction

An AlphaFold2 (98) prediction was used as an initial model for model building into the consensus, open and closed fingers maps. The model was first adjusted into the map in ISOLDE 1.8 (99), then adjusted in COOT 0.9.8.92 (100). Nucleic acid models were generated in COOT, then adjusted into the map using ISOLDE. The model for ddTTP was imported from the REFMAC monomer library in COOT. To allow map blurring and sharpening in COOT, maps were converted from MRC format into MTZ format using REFMAC5.8 (101). ISOLDE and COOT were iteratively used to diagnose and fix errors, and to improve model geometry. For the EN-resolved model, model building was carried out as described above but included adaptive distance and torsion restraints in ISOLDE for the EN domain using a crystal structure (PDB 1VYB) (49) as a reference.

Models were first refined using PHENIX 1.21.1-5286 (102) then Servalcat 0.4.72 (103). All PHENIX refinements were limited to one macro-cycle of global minimization and ADP refinement, using a parameter file generated in ISOLDE. Servalcat refinements included protein secondary structure restraints and nucleic acid restraints calculated using PROSMART and LIBG, respectively (104, 105). Model-vs-map FSCs and EMRinger scores were calculated using PHENIX. Q-scores were calculated in UCSF ChimeraX (106). Model geometries were assessed using the MolProbity server (http://molprobity.biochem.duke.edu/). A summary of the refined models is provided in table S2. The identity of bases in the second primer region and in the “unassigned nucleic acid” could not be unambiguously assigned. Therefore, we have modeled these bases as either A or T.

AlphaFold3 (59) predictions were performed using the webserver interface (https://alphafoldserver.com/) with a randomly generated seed. PAE plots were generated using a modified Python script (https://github.com/nayimgr/af3analysis). Sequences used for AlphaFold3 predictions in Fig. 4 are included in Table S2.

### Map and model visualization

Maps and models were visualized with UCSF ChimeraX. Illustrations were prepared using Adobe Illustrator, ChimeraX and PyMOL (https://www.pymol.org/).

### Sequence alignments

Protein sequences for sequence alignment were downloaded from UniProt (107) or from Boissinot *et al*. (108) and aligned using Clustal Omega (109).

## Supplementary Material

Data S1

MDAR Checklist

Movie S1

Movie S2

Movie S3

Supp Movie Captions

Supplementary Materials

## Data Availability

The cryo-EM maps have been deposited in the Electron Microscopy Data Bank under the following accession codes: EMD-52070 for the consensus TPRT complex, EMD-52072 for the open fingers state, EMD-52071 for the closed fingers state, and EMD-52073 for the EN domain-resolved TPRT complex. The coordinates for the atomic models have been deposited in the Protein Data Bank under the following accession codes: 9HDO for the consensus TPRT complex, 9HDQ for the open fingers state, 9HDP for the closed fingers state, and 9HDR for the EN-resolved TPRT complex. The raw cryo-EM micrographs have been deposited in EMPIAR with accession code EMPIAR-12450. AlphaFold3 predictions have been deposited in ModelArchive with accession codes ma-w0esd (ORF2p-PCNA-target DNA) and ma-lhunr (ORF2p-PABPC1-RRM1-2-RNA). The scripts used in this manuscript are available at Zenodo (110). Materials are available from T.H.D.N. under a material transfer agreement with the MRC-Laboratory of Molecular Biology.
