# Supplementary material for: Structural mechanism of LINE-1 target-primed reverse transcription
Source: Science. Author manuscript; Available in PMC 2025 Jul 4. (PMC7617806; doi:10.1126/science.ads8412)
Supplement: Data S1 [file EMS206034-supplement-Data_S1.pdf]

ORF2p Interactor Summary

Number	Interactor_A	Interactor_B	Uniprot_interactor_B	reported_ipTM	reported_pTM
1	LORF2_HUMAN_1_1276	TIM13_HUMAN_1_96	Q9Y5L4	0.68	0.75
2	LORF2_HUMAN_1_1276	TOM40_HUMAN_1_362	O96008	0.22	0.59
3	LORF2_HUMAN_1_1276	PCNA_HUMAN_1_262	P12004	0.81	0.76
4	LORF2_HUMAN_1_1276	MOV10_HUMAN_1_1004	Q9HCE1	0.31	0.54
5	LORF2_HUMAN_1_1276	HSP7C_HUMAN_1_647	P11142	0.19	0.57
6	LORF2_HUMAN_1_1276	FKBP4_HUMAN_1_460	Q02790	0.17	0.58
7	LORF2_HUMAN_1_1276	HMCES_HUMAN_1_355	Q96FZ2	0.17	0.61
8	LORF2_HUMAN_1_1276	PABP4_HUMAN_1_645	Q13310	0.67	0.65
9	LORF2_HUMAN_1_1276	HS90B_HUMAN_1_725	P08238	0.22	0.57
10	LORF2_HUMAN_1_1276	PURA_HUMAN_1_323	Q00577	0.18	0.65
11	LORF2_HUMAN_1_1276	YMEL1_HUMAN_1_774	Q96TA2	0.20	0.55
12	LORF2_HUMAN_1_1276	TBB4B_HUMAN_1_446	P68371	0.34	0.66
13	LORF2_HUMAN_1_1276	NP1L1_HUMAN_1_392	P55209	0.40	0.65
14	LORF2_HUMAN_1_1276	PARP1_HUMAN_1_1015	P09874	0.21	0.51
15	LORF2_HUMAN_1_1276	NP1L4_HUMAN_1_376	Q99733	0.19	0.61
16	LORF2_HUMAN_1_1276	ZCHC3_HUMAN_1_404	Q9NUD5	0.22	0.60
17	LORF2_HUMAN_1_1276	IPO7_HUMAN_1_1039	O95373	0.41	0.51
18	LORF2_HUMAN_1_1276	RS27A_HUMAN_1_157	P62979	0.38	0.74
19	LORF2_HUMAN_1_1276	PURB_HUMAN_1_313	Q96QR8	0.19	0.65
20	LORF2_HUMAN_1_1276	TBB5_HUMAN_1_445	P07437	0.22	0.62
21	LORF2_HUMAN_1_1276	HS71A_HUMAN_1_642	P0DMV8	0.27	0.60
22	LORF2_HUMAN_1_1276	RENT1_HUMAN_1_1130	Q92900	0.49	0.59
23	LORF2_HUMAN_1_1276	HS90A_HUMAN_1_733	P07900	0.20	0.56
24	LORF2_HUMAN_1_1276	HAX1_HUMAN_1_280	O00165	0.44	0.70
25	LORF2_HUMAN_1_1276	PABP1_HUMAN_1_637	P11940	0.27	0.60
26	LORF2_HUMAN_1_1276	TOP1_HUMAN_1_766	P11387	0.26	0.56
