# Supplementary material for: Structural mechanism of LINE-1 target-primed reverse transcription
Source: Science. Author manuscript; Available in PMC 2025 Jul 4. (PMC7617806; doi:10.1126/science.ads8412)
Supplement: Supp Movie Captions [file EMS206034-supplement-Supp_Movie_Captions.pdf]

Movie S1.

Morph of volumes along the first component from 3D variability analysis in CryoSPARC. Volumes are colored according to domains and regions, as in Fig. 1.

Movie S2.

Morph of volumes along the second component from 3D variability analysis in CryoSPARC. Volumes are colored according to domains and regions, as in Fig. 1.

Movie S3.

Morph of volumes along the third component from 3D variability analysis in CryoSPARC. Volumes are colored according to domains and regions, as in Fig. 1.
