# Supplementary material for: Structural mechanism of LINE-1 target-primed reverse transcription
Source: Science. Author manuscript; Available in PMC 2025 Jul 4. (PMC7617806; doi:10.1126/science.ads8412)
Supplement: Supplementary Materials [file EMS206034-supplement-Supplementary_Materials.pdf]

Science AAAS

Supplementary Materials for

Structural mechanism of LINE-1 target-primed reverse transcription

George E. Ghanim, Hongmiao Hu, Jerome Boulanger, Thi Hoang Duong Nguyen

Corresponding authors: George E. Ghanim, gghanim@princeton.edu; Thi Hoang Duong Nguyen, knguyen@mrc-lmb.cam.ac.uk

Science **388**, eads8412 (2025)
DOI: 10.1126/science.ads8412

The PDF file includes:

Supplementary Text
Figs. S1 to S13
Tables S1 and S2
References

Other Supplementary Material for this manuscript includes the following:

MDAR Reproducibility Checklist
Data S1
Movies S1 to S3

Supplementary Text

The ORF2p TPRT complex adopts a similar architecture as observed in previously published structures (*25*, *26*). However, we note several differences (fig. S6C). Firstly, the EN domain is better resolved in our reconstruction than in previous reconstructions (fig. S6C).

Secondly, we observe target DNA near the CTD and thumb subdomain rather than an RNA hairpin (*26*). Upon close inspections of the published model (PDB 8UW3) and map (EMD-42637) (*26*), we observe that the corresponding density has clear B-form characteristic, highly indicative of DNA, rather than RNA which would be A-form. We do not know how this discrepancy has arisen, given the published experimental details.

Finally, while we observe a similar flexing of the so-called tower and EN domain as previously proposed (*25*), the EN domain appears much more flexible than proposed (3D variability analysis, Movie S1 – S3). Additionally, we observe flexing of the other domains of ORF2p, reminiscent of the open ring and closed ring conformations previously proposed (*25*), but to a much lesser extent. The nucleic acid substrate we used likely stabilizes the complex and limits the extent of flexibility.

Typical PIP box-PCNA interactions are bidentate: where 3-4 hydrophobic residues are buried across a hydrophobic pocket in PCNA, near the PCNA interdomain connector loop (IDCL), while a PIP box glutamine sticks into a region of PCNA called the Q-pocket (*60*, *111*).

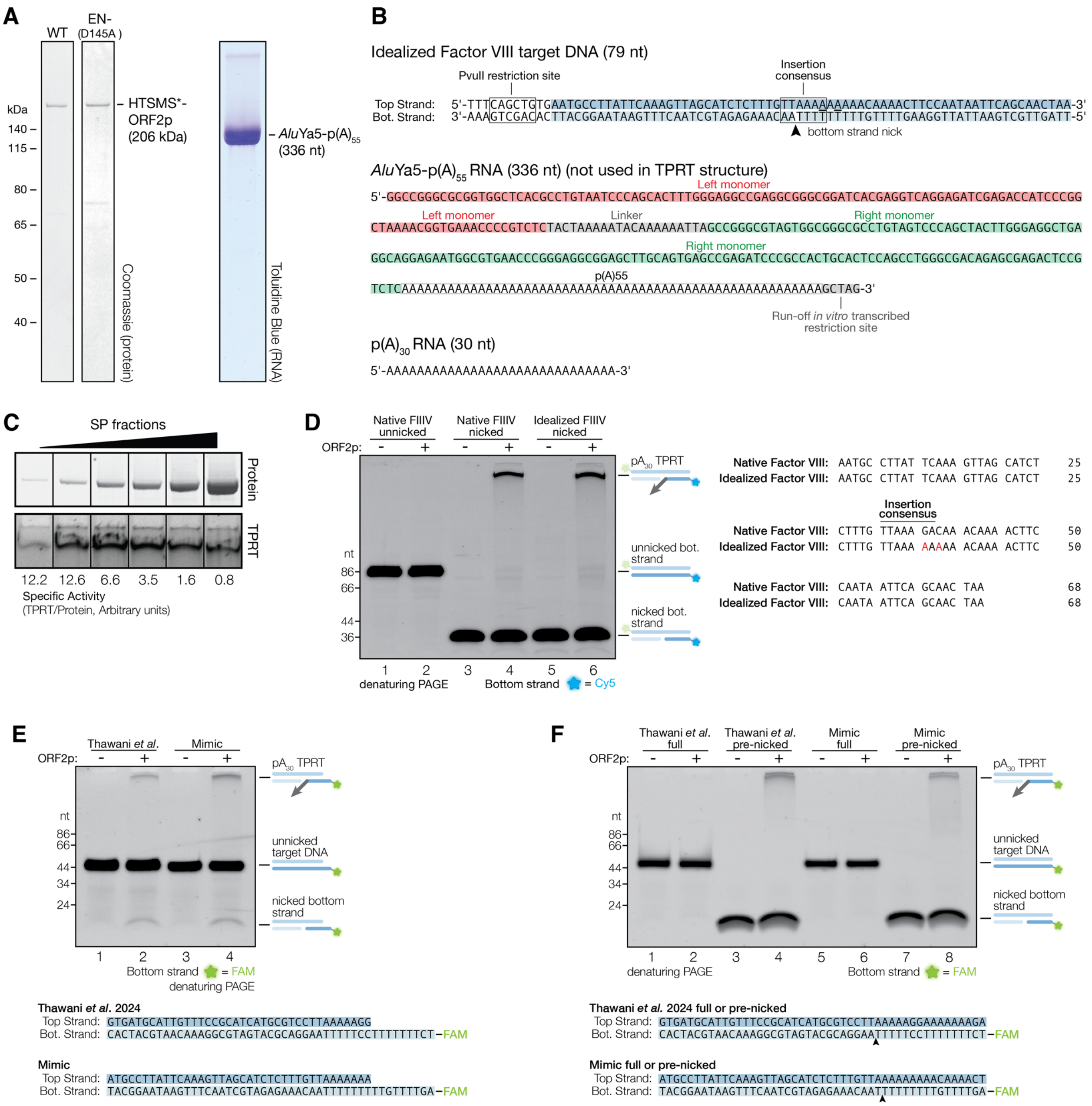

Fig. S1. Purification of ORF2p and TPRT assays on different DNA substrates. (**A**) Coomassie-stained SDS-PAGE gel of purified ORF2p proteins (left). Toluidine blue stained denaturing PAGE of purified *in vitro* transcribed *Alu* RNA. (**B**) Schematic of the nucleic acids used (see table S2 for the full list). The target DNA was idealized from a *de novo* LINE-1 insertion into exon 14 of the human factor VIII gene (*27*). Underlined adenosines indicate a G-to-A, and a C-to-A substitution designed to extend the complementarity with the poly(A) RNA. (**C**) Specific activity of SP cation exchange chromatography fractions. Coomassie stained gel shows ORF2p abundance (Protein, top). Denaturing PAGE of TPRT activity from each fraction (bottom, TPRT). Specific activity was calculated from the ratios of band intensities and is in arbitrary units. Diminished specific activity of later fractions was not caused by increased ORF2p or salt concentrations (data not shown). (**D**) Denaturing PAGE comparing TPRT activity on the native FIIIV DNA or idealized DNA substrate. Sequences of the DNA substrates are shown to the right with modified bases indicated in red. (**E**) Denaturing PAGE comparing TPRT activity on a

substrate with a 3' overhang used in previous studies (*26*). Sequences of the DNA substrates are shown to the bottom. The mimic substrate is the target DNA used in this study but modified to resemble the one used in previous studies. (**F**) Denaturing PAGE comparing TPRT activity on substrates used in previous studies but modified to either be fully double stranded (lanes 1, 2, 5 and 6) or pre-nicked on the bottom strand (lanes 3, 4, 7 and 8). Sequences of the DNA substrates are shown at the bottom.

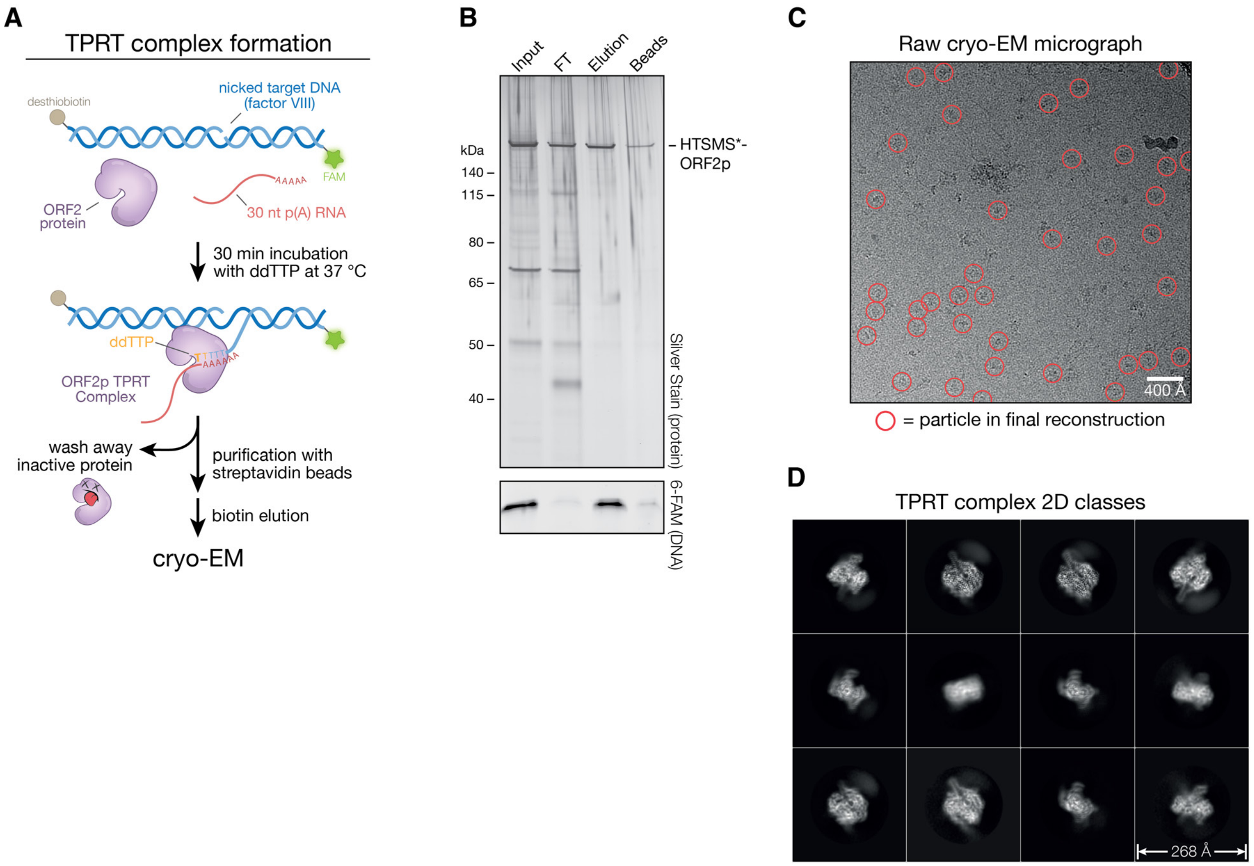

Fig. S2. Purification of ORF2p TPRT complex for cryo-EM. (**A**) Strategy for assembling and purifying of the ORF2p TPRT complex. (**B**) Top panel shows protein fractions from the TPRT purification, visualized by silver-stained SDS-PAGE. Bottom panel shows DNA fractions, visualized by FAM fluorescence. FT, streptavidin resin flowthrough. (**C**) Representative cryo-EM micrograph. Red circles indicate particles used in the final consensus reconstruction. (**D**) Representative cryo-EM 2D class averages of the TPRT complex.

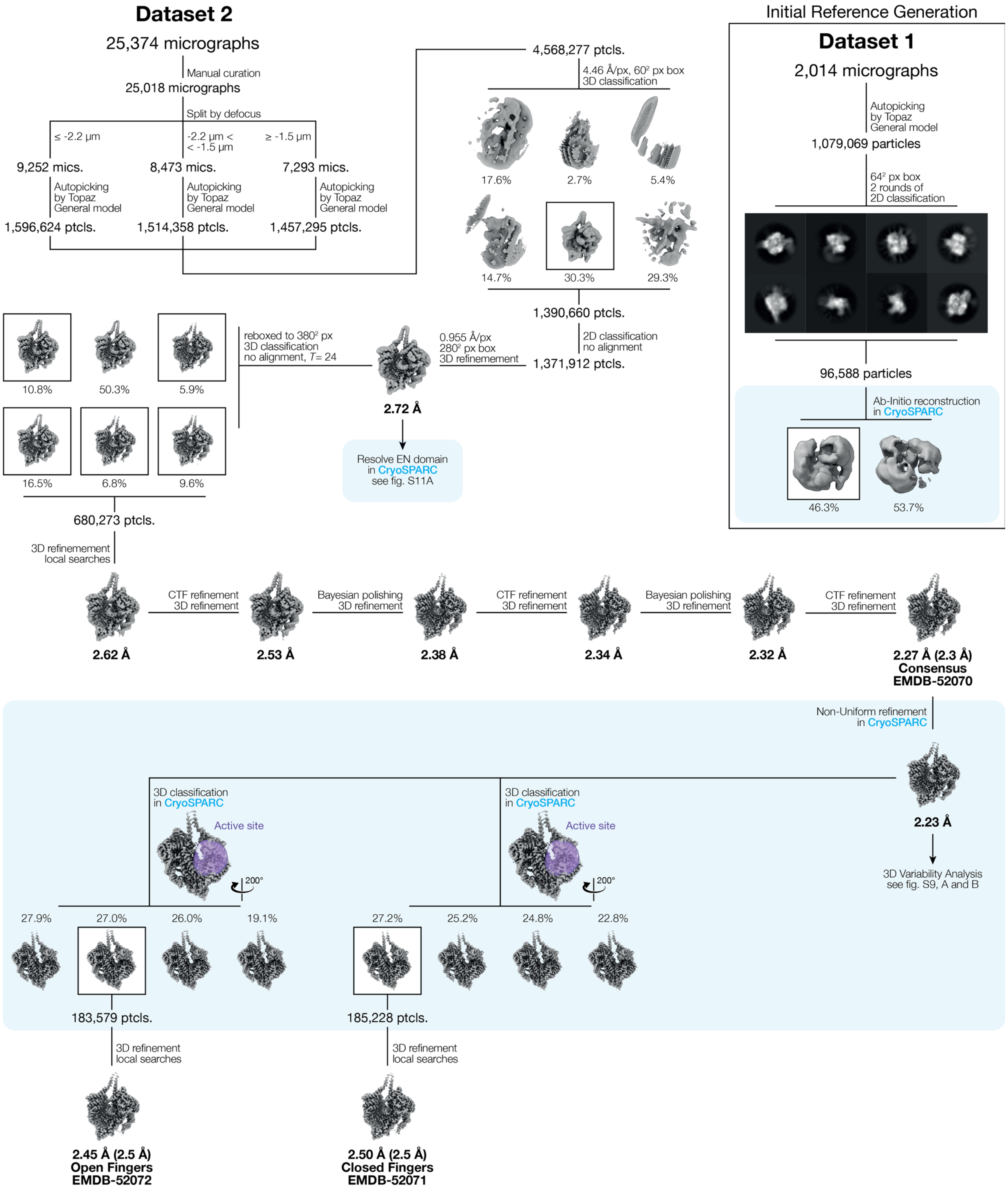

Fig. S3. Cryo-EM data processing pipeline. Blue shading indicates data processing steps performed in CryoSPARC. Final reconstructions deposited to the EMDB are indicated.

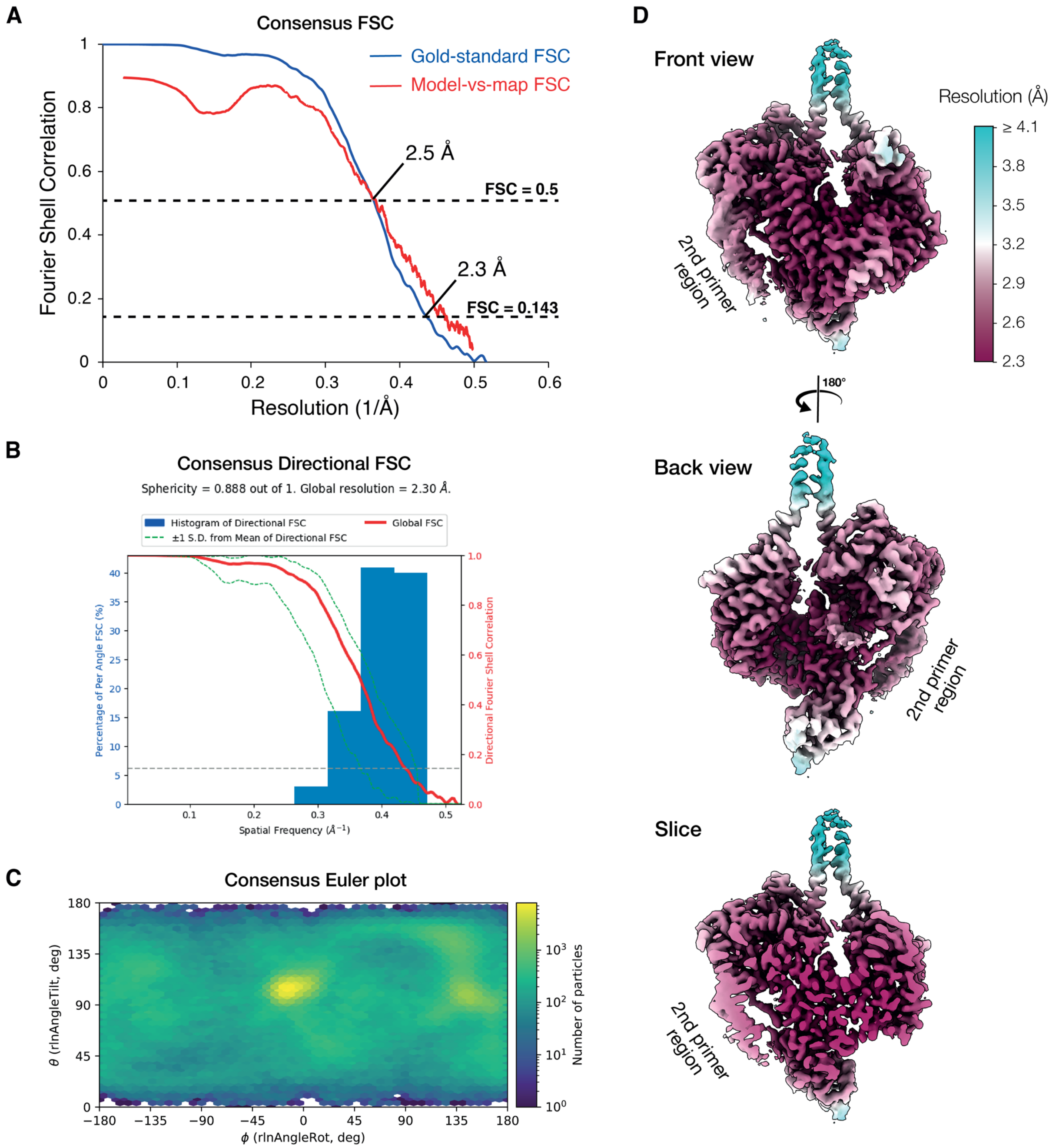

Fig. S4. Overall and local resolutions of the consensus cryo-EM map. (**A**) Gold-standard (blue) and model-vs-map (red) Fourier Shell Correlation (FSC) plots. Resolution was estimated at FSC = 0.143 (gold-standard) and at FSC = 0.5 (model-vs-map). (**B**) Directional FSC plots and sphericity values, calculated using a 3D-FSC webserver (https://3dfsc.salk.edu/) (*97*). (**C**) 2D histograms of the orientation distribution (Euler angles), calculated using a Python script (https://github.com/Guillawme/angdist). (**D**) Unsharpened cryo-EM maps colored by local resolution. RELION was used for local resolution estimation (*112*).

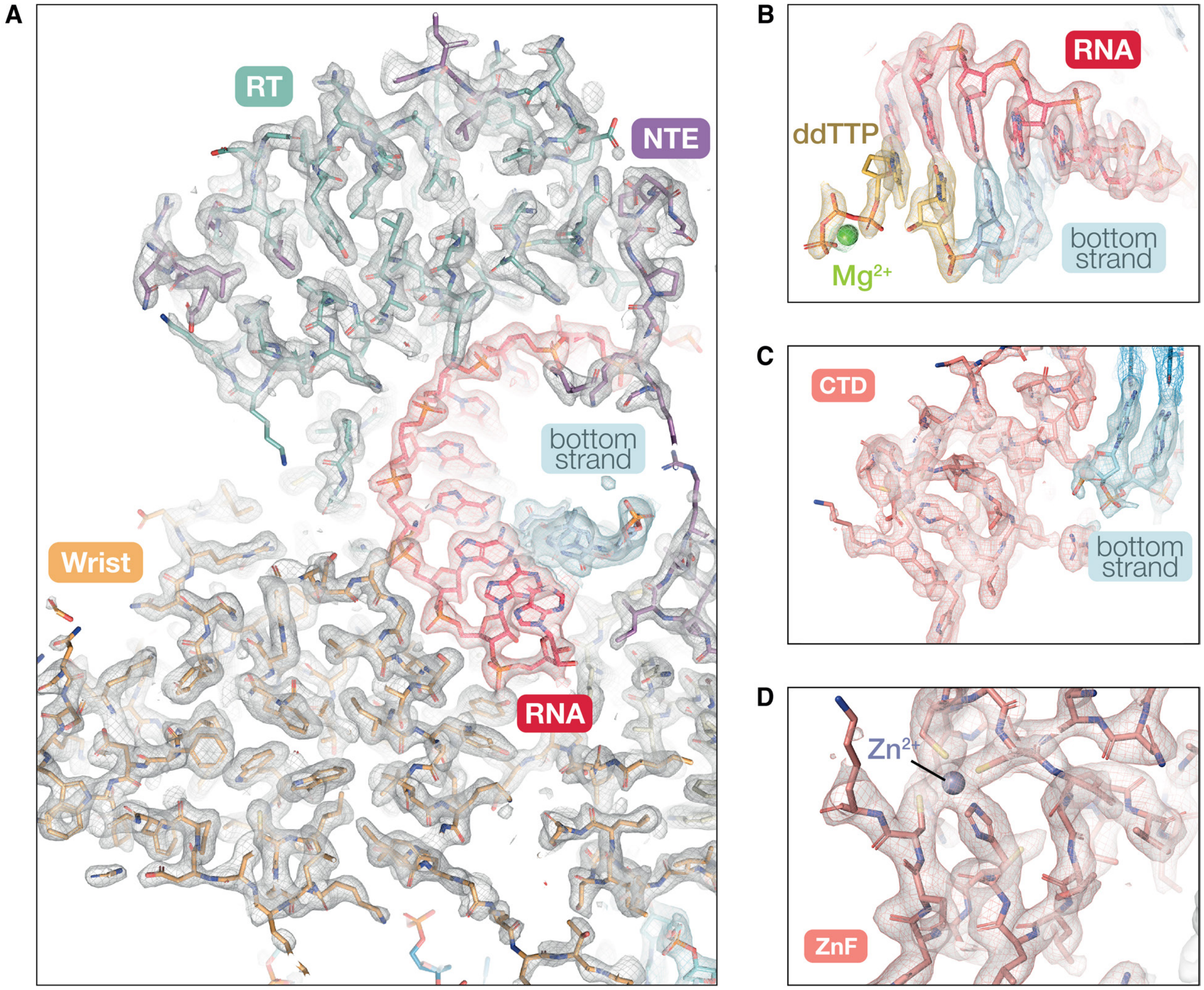

Fig. S5. Quality of the consensus cryo-EM map and corresponding model. (**A–D**) Representative cryo-EM densities for different regions of the TPRT complex consensus structure.

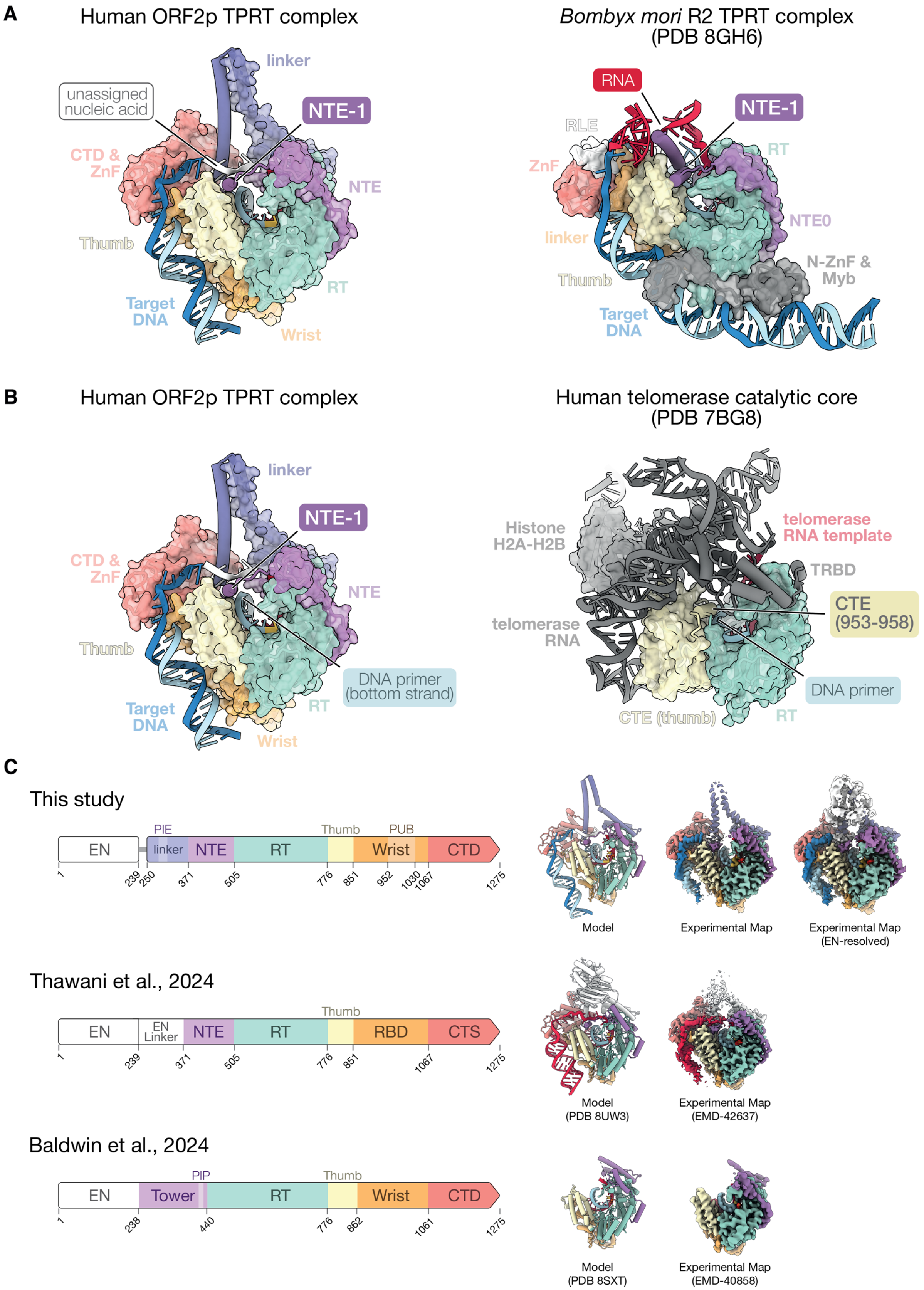

Fig. S6. Comparison between ORF2p TPRT complex and the structures of other RTs. (**A**) Comparison to NTE-1 of the *Bombyx mori* R2 (*Bm*R2) retrotransposon TPRT complex (*35*). The

domains of *Bm*R2 are colored by their similarity to ORF2p. RLE, restriction-like endonuclease; N-ZnF, N-terminal C2H2 zinc finger DNA-binding domain; Myb, Myb DNA-binding domain. (**B**) Comparison to CTE (residues 952–958) of the human telomerase catalytic core (*36*). TRBD, telomerase RNA-binding domain; CTE, C-terminal extension. (**C**) Comparison to other ORF2p structures. RBD, RNA binding domain; CTS, C-terminal segment; PIP, PCNA-interacting peptide; CTD, carboxy-terminal domain.

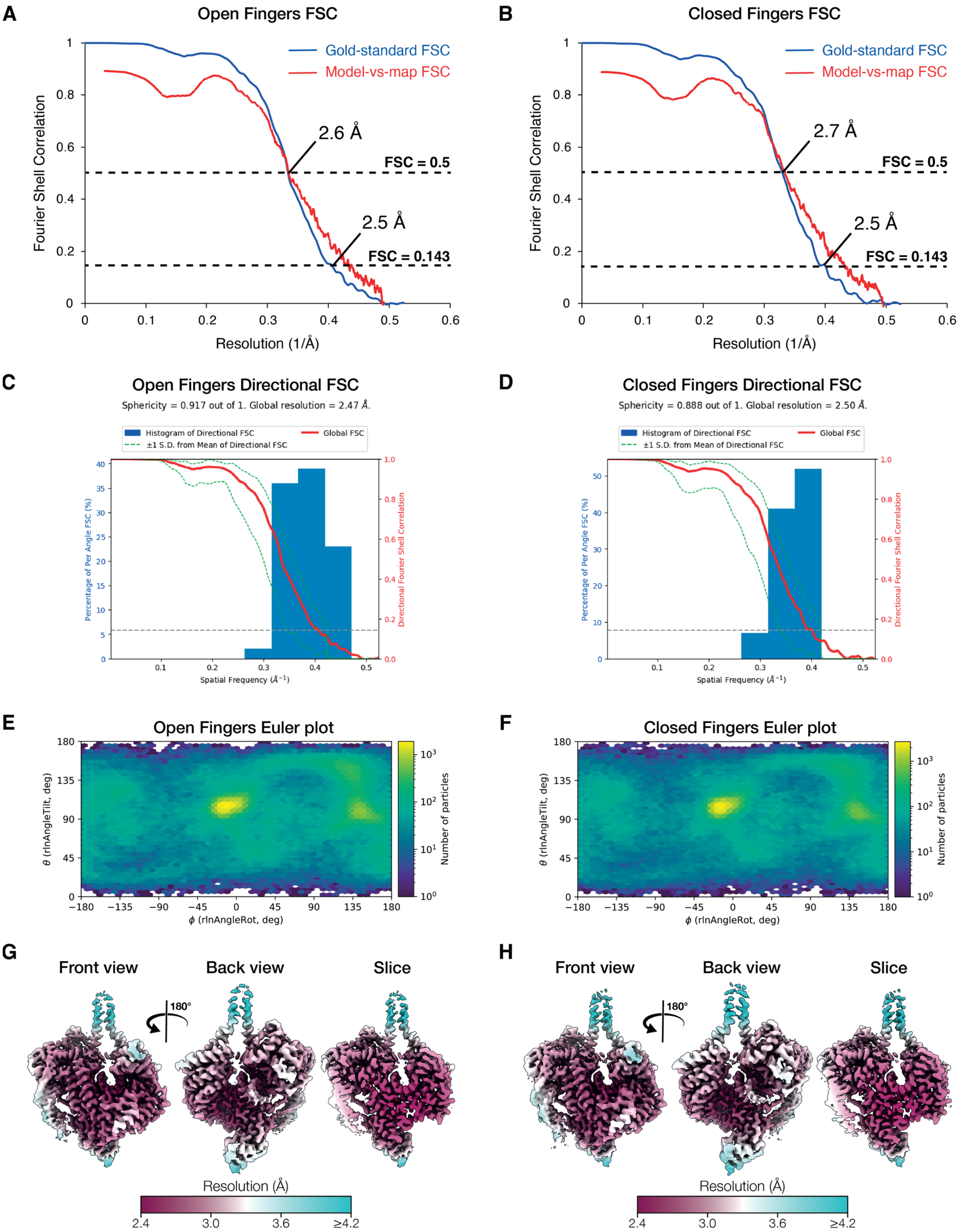

Fig. S7. Overall and local resolutions of the open fingers and closed fingers cryo-EM maps. (**A** and **B**) Gold-standard (blue) and model-vs-map (red) Fourier Shell Correlation (FSC) plots for the TPRT complex in the open fingers and closed fingers state, respectively. Resolution was estimated at FSC = 0.143 (gold-standard) and at FSC = 0.5 (model-vs-map). (**C** and **D**) Directional

FSC plots and sphericity values for the TPRT complex in the open fingers and closed fingers state, respectively. Plots were calculated using a 3D-FSC webserver (https://3dfsc.salk.edu/) (*97*). (**E** and **F**) 2D histograms of the orientation distribution (Euler angles) for the TPRT complex in the open fingers and closed fingers state, respectively. Plots were calculated using a Python script (https://github.com/Guillawme/angdist). (**G** and **H**) Unsharpened cryo-EM maps of the TPRT complex open fingers and closed fingers state, respectively, colored by local resolution. RELION was used for local resolution estimation (*112*).

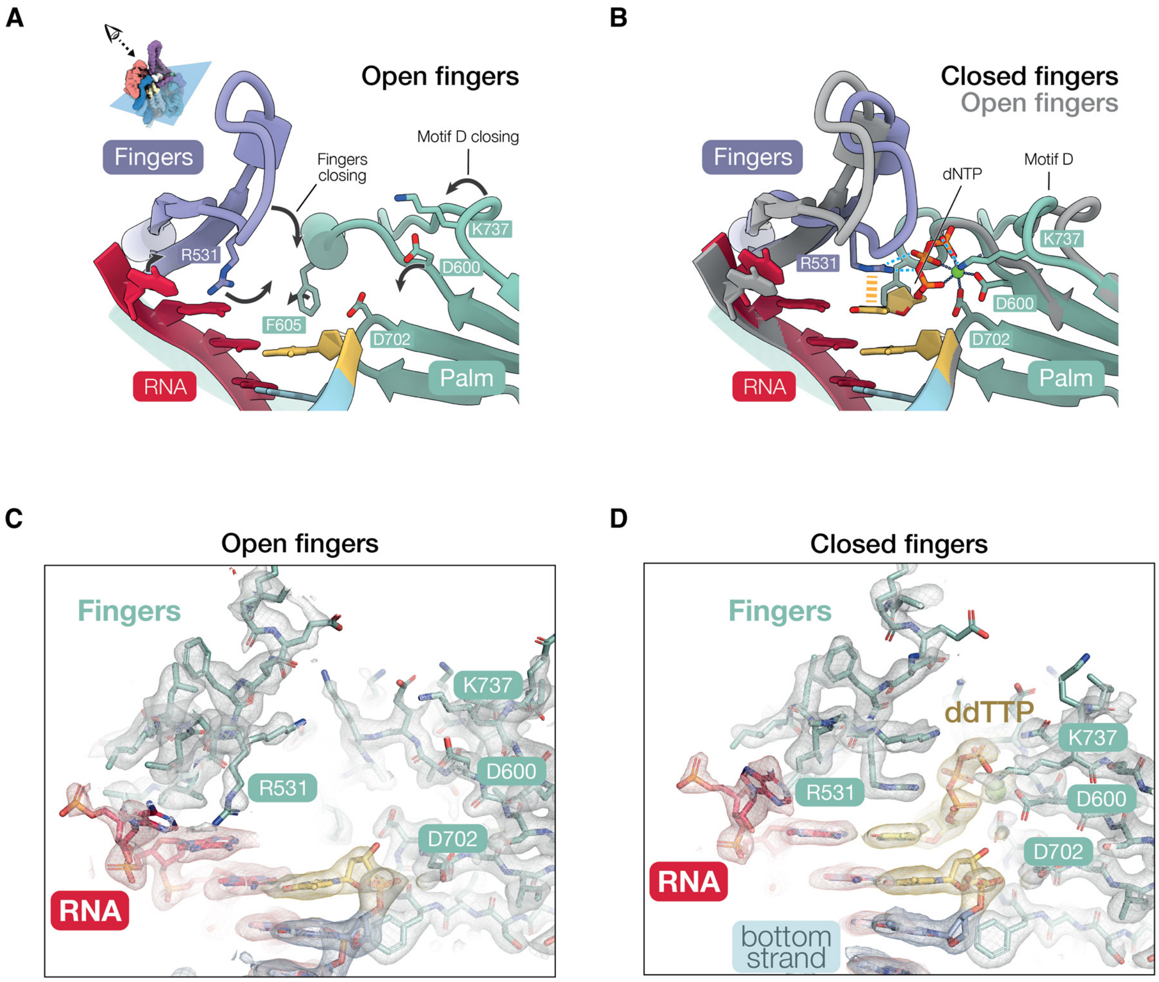

Fig. S8. Quality of the open fingers and closed fingers cryo-EM maps and models. (**A**) Detailed view of the RT active site in the open fingers configuration. Arrows indicate change from the open fingers configuration to the closed fingers configuration. (**B**) Detailed view of the RT active site in the closed fingers configuration. The open configuration is shown in grey. (**C**) Representative cryo-EM densities for the open fingers map, focusing on the RT active site. (**D**) Representative cryo-EM densities for the closed fingers map, focusing on the RT active site.

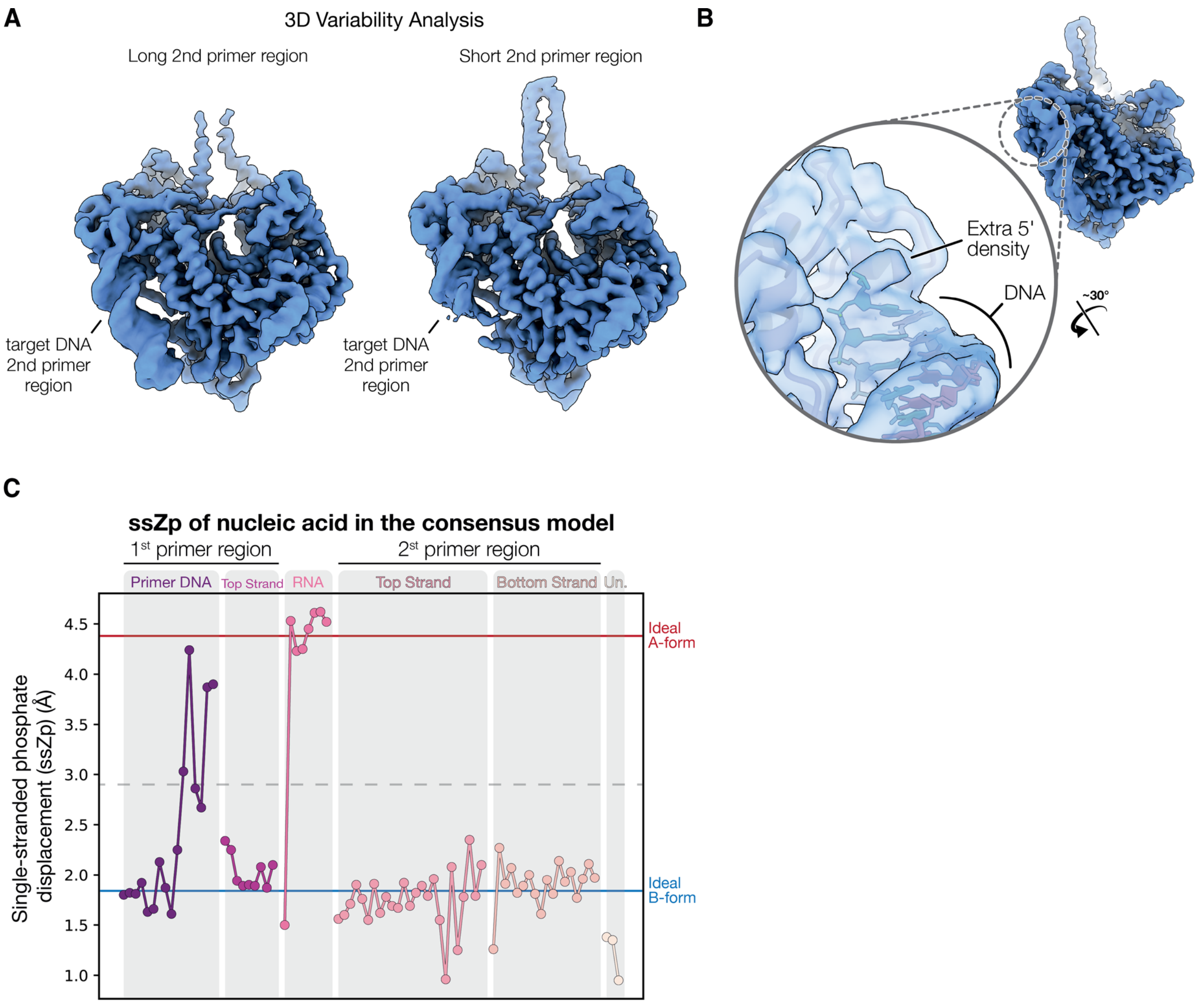

Fig. S9. Melted top strand is B-form. (**A**) Maps showing heterogeneity in the length of the target DNA 2nd primer region. Heterogeneity was explored using 3D variability analysis (3DVA) in CryoSPARC (*113*). (**B**) Extra 5' density at the target DNA 2nd primer region observed during 3DVA. This density is likely nucleotides from a melted bottom strand; however, they could not be confidently modeled due to their apparent flexibility. (**C**) Single-stranded phosphate displacement (ssZp) (*114*) plots to assess the sugar pucker of the nucleic acids within the TPRT complex. ssZp is small and positive for a C2'-endo sugar, as in B-form DNA; ssZp is large and positive for a C3'-endo sugar, as in A-form DNA and RNA. The values for ideal A-form (red, ssZp = 4.38 Å), and ideal B-form (blue, ssZp = 1.84 Å) are indicated. ssZp values were calculated using the x3DNA webserver (http://web.x3dna.org/).

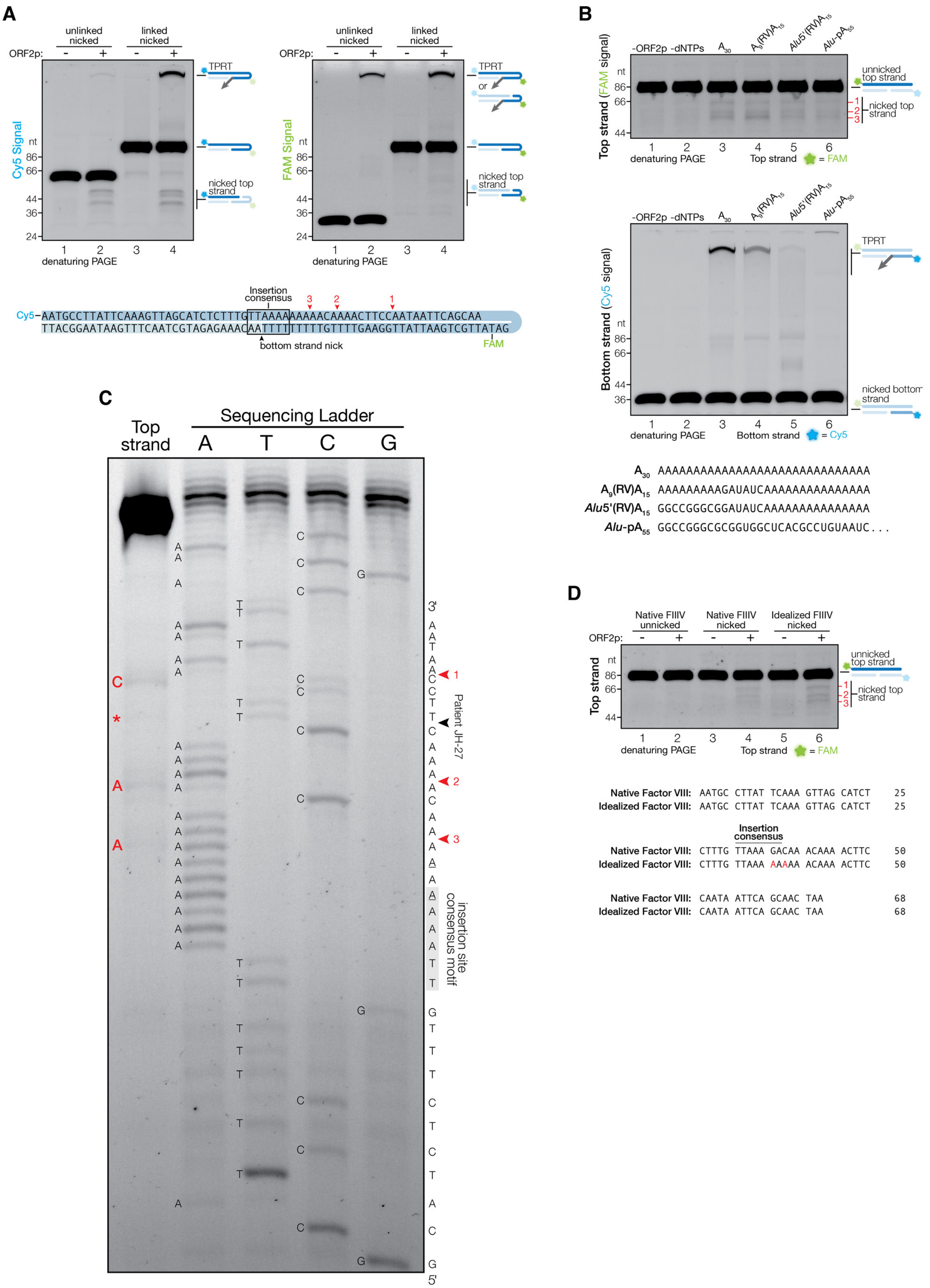
A
unlinked nicked
linked nicked
ORF2p: - + - +
TPRT
nicked top strand
Cy5 Signal
FAM Signal
or
nt
86
66
44
36
24
1 2 3 4
denaturing PAGE
Insertion consensus
3 2 1
Cy5–AATGCCTTATTCAAAGTTAGCATCTCTTTGTTAAAAAAAAACAAAACTTCCAATAATTCAGCAA
TTACGGAATAAGTTTCAATCGTAGAGAAACAATTTTTTTTTGTTTTGAAGGTTATTAAGTCGTTATAG
bottom strand nick
FAM
B
-ORF2p -dNTPs A30 A9(RV)A15 Alu5'(RV)A15 Alu-pA55
Top strand (FAM signal)
unnicked top strand
nicked top strand
1 2 3 4 5 6
denaturing PAGE
Top strand = FAM
Bottom strand (Cy5 signal)
TPRT
nicked bottom strand
Bottom strand = Cy5
A30 AAAAAAAAAAAAAAAAAAAAAAAAAAAAAA
A9(RV)A15 AAAAAAAAAGAUAUCAAAAAAAAAAAAAAA
Alu5'(RV)A15 GGCCGGGCGGAUAUCAAAAAAAAAAAAAAA
Alu-pA55 GGCCGGGCGCGGUGGCUCACGCCUGUAAUC. . .
C
Top strand
Sequencing Ladder
A T C G
3'
Patient JH-27
insertion site consensus motif
5'
D
Native FIIIV unnicked
Native FIIIV nicked
Idealized FIIIV nicked
ORF2p: - + - + - +
Top strand
unnicked top strand
nicked top strand
1 2 3 4 5 6
denaturing PAGE
Top strand = FAM
Native Factor VIII: AATGC CTTAT TCAAA GTTAG CATCT 25
Idealized Factor VIII: AATGC CTTAT TCAAA GTTAG CATCT 25
Insertion consensus
Native Factor VIII: CTTTG TTAAA GACAA ACAAA ACTTC 50
Idealized Factor VIII: CTTTG TTAAA AAAAA ACAAA ACTTC 50
Native Factor VIII: CAATA ATTCA GCAAC TAA 68
Idealized Factor VIII: CAATA ATTCA GCAAC TAA 68

Fig. S10. Characterization of top strand nicking. (**A**) Top strand nicking occurs in *cis*. Denaturing gel of TPRT assays with unlinked (lanes 1 and 2) or linked (lanes 3 and 4) doubly fluorescently labeled target DNA, visualized by Cy5 fluorescence to show top strand nicking (left) or by FAM fluorescence to show bottom strand TPRT products (right). The linked DNA substrate used in this assay is depicted (bottom). The absence of strong signal for products between 36 and 66 nt (FAM signal, lane 4) indicates that top strand nicking occurs in *cis* and that substrates nicked on the top strand (Cy5 signal, lane 4) are efficiently extended on the bottom strand (FAM signal, lane 4, TPRT products). However, the TPRT product signal (Cy5 signal, lane 4) may indicate that not all TPRT products are nicked on the top strand. (**B**) Denaturing gel of TPRT assays with different RNA substrates showing that the RNA 5' sequence does not affect the top strand cleavage pattern. RNA sequences used in this assay are shown (bottom). (**C**) Denaturing sequencing gel of top strand cleavage products visualized by FAM fluorescence. Mapped top strand cleavage sites are indicated by red arrows. Sequencing reactions (ATCG) are shown as markers. Relevant target DNA sequence is shown to the right and aligned with the sequencing markers. The putative top strand cleavage site for the *de novo* insertion in patient JH-27 (*27*) is indicated. A weak cleavage product adjacent to the putative JH-27 cleavage site is indicated with a star (*). (**D**) Denaturing gel of TPRT assays showing that the top strand cleavage pattern is not affected by idealization of the target DNA substrate. Native and idealized FIIIV substrate sequences are shown (bottom).

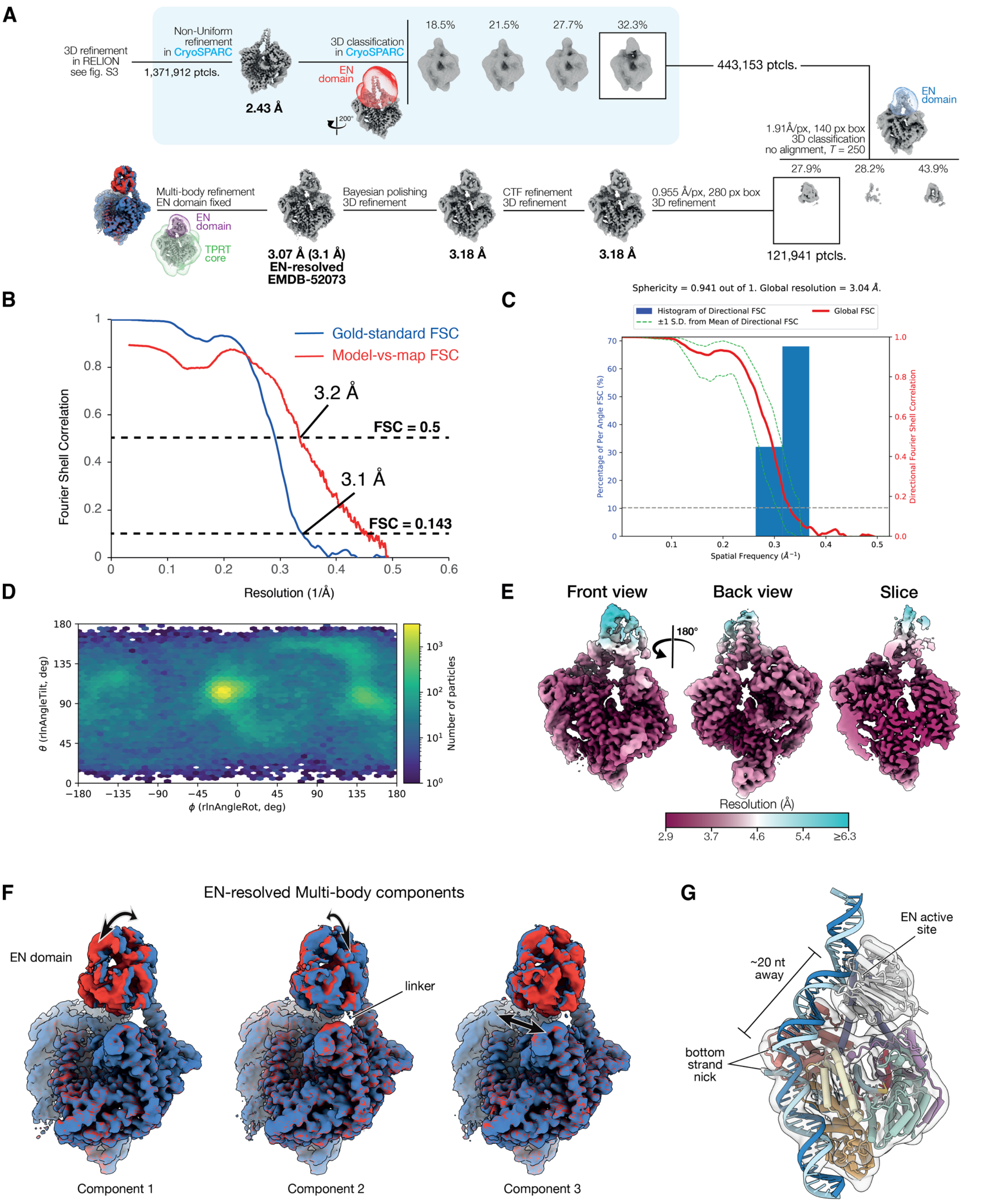

Fig. S11. Processing pipeline, resolution estimates and flexibility of the EN-resolved cryo-EM map. (**A**) Cryo-EM data processing pipeline used to resolve the EN domain. Blue shading indicates data processing steps performed in CryoSPARC. Final reconstructions deposited to the EMDB are indicated. (**B**) Gold-standard (blue) and model-vs-map (red) Fourier Shell Correlation (FSC) plots. (**C**) Directional FSC plots and sphericity values. Plots were calculated using a 3D-

FSC webserver (https://3dfsc.salk.edu/) (*97*). (**D**) 2D histograms of the orientation distribution (Euler angles), calculated using a Python script (https://github.com/Guillawme /angdist). (**E**) Unsharpened cryo-EM maps colored by local resolution. RELION was used for local resolution estimation (*112*). (**F**) Motion along the principal components from multi-body refinement in RELION. Arrows indicate the direction of motion. (**G**) Modeling of nicked target DNA into the EN-resolved map positions the EN domain ~ 20 nt away from bottom strand nick.

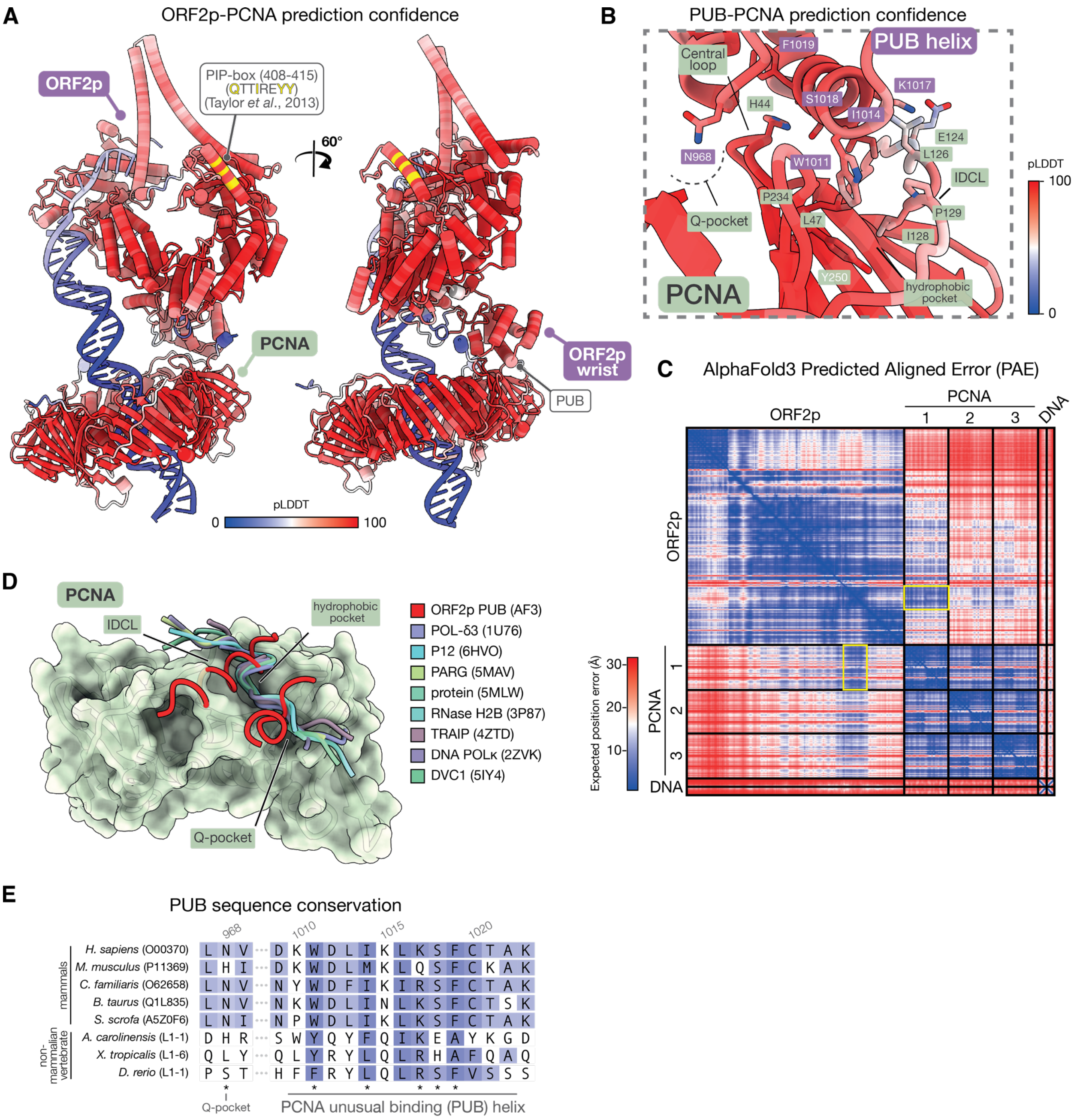

Fig. S12. ORF2p and PCNA AlphaFold3 prediction. (**A**) AlphaFold 3 prediction of ORF2p, PCNA, and target DNA, as in Fig. 4A, except colored by local confidence (predicted local distance difference test, pLDDT). Higher pLDDT values indicate higher accuracy (red), whereas lower values indicate lower accuracy (blue). Alternate PIP-box identified in a previous study is indicated in yellow. (**B**) Detailed view of the PUB-PCNA interaction as in Fig. 4B, except colored by local confidence. (**C**) Predicted aligned error (PAE) plot for the ORF2p-PCNA prediction. Lower PAE values indicate higher accuracy (blue), whereas higher values indicate lower accuracy (red). The PUB-PCNA interaction is boxed in yellow. (**D**) Superimposition of ORF2p PUB-PCNA interaction against the structures of various PIP peptide-PCNA interactions. PDB ID from where each structure was derived are indicated. (**E**) PUB helix sequence conservation across diverse species. Amino acid numbering is relative to human ORF2p. PCNA interacting residues are labeled with a star (*).

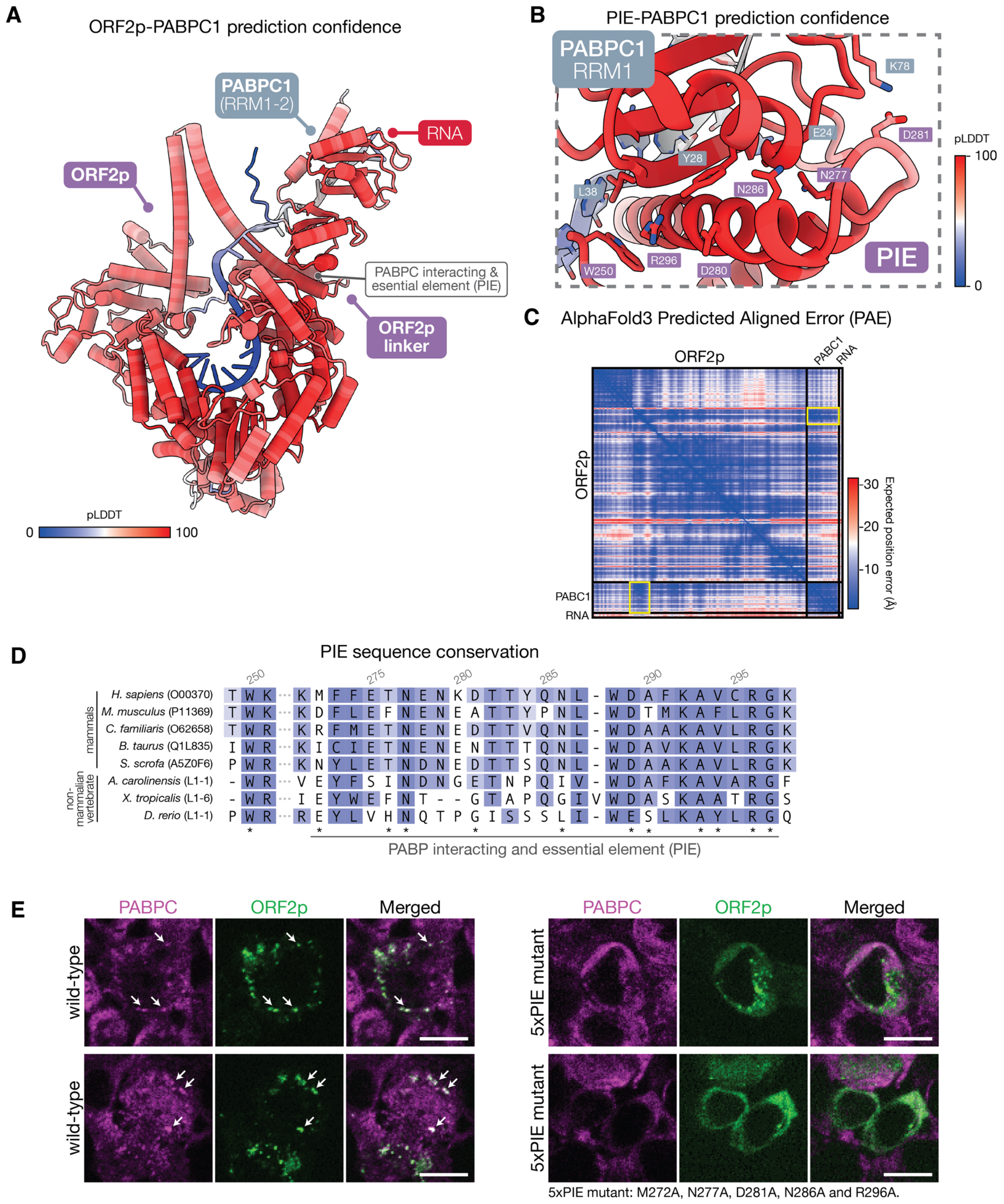

Fig. S13. ORF2p and PABPC1 AlphaFold3 prediction. (**A**) AlphaFold3 prediction of ORF2p, PABPC1 and RNA, as in Fig. 4E, except colored by local confidence (predicted local distance difference test, pLDDT). Higher pLDDT values indicate higher accuracy (red), whereas lower values indicate lower accuracy (blue). (**B**) Detailed view of the PIE-PABPC1 interaction as in Fig. 4F, except colored by local confidence. (**C**) Predicted aligned error (PAE) plot for the ORF2p-PABPC1 prediction. Lower PAE values indicate higher accuracy (blue), whereas higher values indicate lower accuracy (red). The PIE-PABPC1 interaction is boxed in yellow. (**D**) PIE sequence conservation across diverse species. Amino acid numbering is relative to human ORF2p. PABPC

interacting residues are labeled with a star (*). (**E**) Additional examples of ORF2p (wild-type or 5xPIE mutant; green) and PABPC (magenta) immunofluorescence staining. Arrows indicate examples of ORF2p cytoplasmic puncta co-localized with PABPC, which were not observed in the ORF2p 5xPIE mutant. 5xPIE mutant, M272A, N277A, D281A, N286A and R296A. Scale bar: 10 µm.

Table S1. Cryo-EM data collection, refinement and validation statistics.

	Consensus<br>EMD-52070<br>PDB 9HDO	Open Fingers<br>EMD-52072<br>PDB 9HDQ	Closed Fingers<br>EMD-52071<br>PDB 9HDP	EN-resolved<br>EMD-52073<br>PDB 9HDR
**Data collection and Processing**				
Microscope	Krios G4			
Voltage (keV)	300			
Camera	Falcon 4i			
Magnification	130,000			
Pixel size at detector (Å/pixel)	0.955			
Total electron exposure (e–/Å^2)	59.22			
Exposure rate (e-/ Å^2/sec)	10.12			
Number of frames	50			
Defocus range (μm)	1.8-2.2			
Automation software	EPU			
Energy filter slit width (eV)	10 eV			
Micrographs collected (no.)	25,374			
Total extracted particles (no.)	4,568,277			
**For each reconstruction**				
Final particles (no.)	680,273	183,579	185,228	121,941
Point-group	C1	C1	C1	C1
Estimated error (rotations/translations)	0.906/0.356215	0.854/0.339980	0.853/0.339025	1.50/0.482275
Resolution (global, Å)	2.27	2.45	2.5	3.07
FSC 0.5 (unmasked/masked)	2.71/2.49	2.99/2.63	3.01/2.69	3.30/3.17
FSC 0.143 (unmasked/masked)	2.17/2.01	2.30/2.15	2.32/2.16	2.82/2.64
Resolution range (local, Å)	2.26-7.36	2.36-8.75	2.39-8.19	2.90-6.84
3DFSC Sphericity	0.888	0.917	0.888	0.941
Map sharpening B factor (Å^2)	-37.7162	-31.2032	-40.8577	-30
Map sharpening methods	RELION5.0	RELION5.0	RELION5.0	RELION5.0
**Model composition**				
Protein (residues)	1025	1025	1025	1263
RNA/DNA (nucleotides)	86	85	86	86
**Model Refinement**				
Refinement package	Phenix1.21/ Servalcat0.4.72	Phenix1.21/ Servalcat0.4.72	Phenix1.21/ Servalcat0.4.72	Phenix1.21/ Servalcat0.4.72
Real or reciprocal space	real/reciprocal	real/reciprocal	real/reciprocal	real/reciprocal
Resolution cutoff	0.5	0.5	0.5	0.5
Model-Map scores				
CCvolume/mask	0.77/0.78	0.79/0.81	0.75/0.77	0.84/0.86
B factors (Å2)				
Protein residues (min/max/mean)	18.69/228.63/72.88	16.26/250.58/72.24	15.24/223.53/71.34	18.32/394.13/142.51
RNA/DNA (min/max/mean)	32.13/226.82/107.02	23.87/235.32/85.00	25.01/177.88/82.54	30.98/333.55/156.70
Ligand (min/max/mean)	56.65/60.50/58.57	58.28/58.28/58.28	34.42/62.54/48.48	76.08/99.33/87.70
R.m.s. deviations from ideal values				
Bond lengths (Å) (#>4σ)	0.008 (6)	0.007 (0)	0.008 (6)	0.008 (5)

Bond angles (°) (#>4σ)	1.335 (3)	1.312 (2)	1.329 (2)	1.241 (1)
**Validation**				
MolProbity score	0.69	0.82	0.75	0.71
CaBLAM outliers (%)	0.69	0.39	0.69	0.56
Clashscore	0.55	1.1	0.8	0.63
Poor rotamers (%)	0.11	0	0	0
C-beta deviations (%)	0	0	0	0
Q-score	0.72	0.71	0.69	0.58
EMRinger score	4.83	4.58	4.01	3.74
Ramachandran plot				
Favored (%)	99.61	99.61	99.12	99.6
Outliers (%)	0	0	0	0

Table S2. Protein and nucleic acid sequences used in this manuscript.

ID	Name	Sequence	Figure
414	HTSMS*-ORF2p	MVHHHHHHHHGGSAWSHPQFEKGGGSGGGSGGSAWSHPQFEKGGSEEGKLVIWINGDKGYNGLAEVGKKFEKDTGIKVTVEHPDKLEEKFPQVAATGDGPDIIFWAHDRFGGYAQSGLLAEITPDKAFQDKLYPFTWDAVRYNGKLIAYPIAVEALSLIYNKDLLPNPPKTWEEIPALDKELKAKGKSALMFNLQEPYFTWPLIAADGGYAFKYENGKYDIKDVGVDNAGAKAGLTFLVDLIKNKHMNADTDYSIAEAAFNKGETAMTINGPWAWSNIDTSKVNYGVTVLPTFKGQPSKPFVGVLSAGINAASPNKELAKEFLENYLLTDEGLEAVNKDKPLGAVALKSYEEELVKDPRIAATMENAQKGEIMPNIPQMSAFWYAVRTAVINAASGRQTVDEALKDAQTNSSSNNNNNNNNNNLGSDSEVNQEAKPEVKPEVKPETHINLKVSDGSSEIFFKIKKTTPLRRLMEAFAKRQGKEMDSLTFLYDGIEIQADQTPEDLDMEDNDIIEAHREQIGGMTGSTSHITILTLNINGLNSAIKRHRLASWIKSQDPSVCCIQETHLTCRDTHRLKIKGWRKIYQANGKQKKAGVAILVSDKTDFKPTKIKRDKEGHYIMVKGSIQQEELTILNIYAPNTGAPRFIKQVLSDLQRDLDSHTLIMGDFNTPLSTLDRSTRQKVNKDTQELNSALHQADLIDIYRTLHPKSTEYTFFSAPHHTYSKIDHIVGSKALLSKCKRTEIITNYLSDHSAIKLELRIKNLTQSRSTTWKLNNLLLNDYWVHNEMKAEIKMFFETNENKDTTYQNLWDAFKAVCRGKFIALNAYKRKQERSKIDTLTSQLKELEKQEQTHSKASRRQEITKIRAELKEIETQKTLQKINESRSWFFERINKIDRPLARLIKKKREKNQIDTIKNDKGDITTDPTEIQTTIREYYKHLYANKLENLEEMDTFLDTYTLPRLNQEEVESLNRPITGSEIVAIINSLPTKKSPGPDGFTAEFYQRYKEELVPFLLKLFQSIEKEGILPNSFYEASIILIPKPGRDTTKKENFRPISLMNIDAKILNKILANRIQQHIKKLIHHDQVGFIPGMQGWFNIRKSINVIQHINRAKDKNHMIISIDAEKAFDKIQQPFMLKTLNKLGIDGTYFKIIRAIYDKPTANIILNGQKLEAFPLKTGTRQGCPLSPLLFNIVLEVLARAIRQEKEIKGIQLGKEEVKLSLFADDMIVYLENPIVSAQNLLKLISNFSKVSGYKINVQKSQAFLYTNNRQTESQIMGELPFTIASKRIKYLGIQLTRDVKDLFKENYKPLLKEIKEETNKWKNIPCSWVGRINIVKMAILPKVIYRFNAIPIKLPMTFFTELEKTTLKFIWNQKRARIAKSILSQKNKAGGITLPDFKLYYKATVTKTAWYWYQNRDIDQWNRTEPSEIMPHIYNYLIFDKPEKNKQWGKDSLFNKWCWENWLAICRKLKLDPFLTPYTKINSRWIKDLNVKPKTIKTLEENLGITIQDIGVGKDFMSKTPKAMATKDKIDKWDLIKLKSFCTAKETTIRVNRQPTTWEKIFATYSSDKGLISRIYNELKQIYKKKTNNPIKKWAKDMNRHFSKEDIYAAKKHMKKCSSSLAIREMQIKTTMRYHLTPVRMAIIKKSGNNRCWRGCGEIGTLLHCWWDCKLVQPLWKSVWRFLRDLELEIPFDPAIPLLGIYPNEYKSCCYKDTCTRMFIAALFTIAKTWNQPKCPTMIDWIKKMWHIYTMEYYAAIKNDEFISFVGTWMKLETIILSKLSQEQKTKHRIFSLIGGN*	Throughout
401	AluYa5-p(A)55-NheI RNA	GGCCGGGCGCGGTGGCTCACGCCTGTAATCCCAGCACTTTGGGAGGCCGAGGCGGGCGGATCACGAGGTCAGGAGATCGAGACCATCCCGGCTAAAACGGTGAAACCCCGTCTCTACTAAAAATACAAAAAATTAGCCGGGCGTAGTGGCGGGCGCCTGTAGTCCCAGCTACTTGGGAGGCTGAGGCAGGAGAATGGCGTGAACCCGGGAGGCGGAGCTTGCAGTGAGCCGAGATCCCGCCACTGCACTCCAGCCTGGGCGACAGAGCGAGACTCCGTCTCAAAAAAAAAAAAAAAAAAAAAAAAAAAAAAAAAAAAAAAAAAAAAAAAAAAAAAAGCTAG	1C
	pA30 RNA	rArArArArArArArArArArArArArArArArArArArArArArArArArArArArArArArA	Throughout
29	FVIII-cons-ideal#2 TOP	tgctagcgcagctgtgAATGCCTTATTCAAAGTTAGCATCTCTTTGTTAAAAAAAAACAAAACTTCCAATAATTCAGCAACTAA/3Phos/	1C - E, S1C
30	FVIII- cons - ideal#2 nick1 BOT	/56-FAM/TTAGTTGCTGAATTATTGGAAGTTTTGTTTTTTTTT	1C - E, S1C
31	FVIII- cons - ideal#2 nick2 BOT	/5Phos/AACAAAGAGATGCTAACTTTGAATAAGGCATTcacagctgcgctagca/3Phos/	1C - E, S1C
32	FVIII- cons - ideal#2 full BOT	/56-FAM/TTAGTTGCTGAATTATTGGAAGTTTTGTTTTTTTTTAACAAAGAGATGCTAACTTTGAATAAGGCATTcacagctgcgctagca/3Phos/	1E
33	FVIII- cons - ideal#2 TOP MUT	tgctagcgcagctgtgAATGCCTTATTCAAAGTTAGCATCTCTTTGCCGGCGCGAAACAAAACTTCCAATAATTCAGCAACTAA/3Phos/	1E
34	FVIII- cons - ideal#2 nick1 BOT MUT	/56-FAM/TTAGTTGCTGAATTATTGGAAGTTTTGTTTCGCGCC	1E
35	FVIII- cons - ideal#2 nick2 BOT MUT	/5Phos/GGCAAAGAGATGCTAACTTTGAATAAGGCATTcacagctgcgctagca/3Phos/	1E
6	FVIII- cons - ideal#1 dbio-TOP	/5deSBioTEG/tttcagctgtgAATGCCTTATTCAAAGTTAGCATCTCTTTGTTAAAAAAAAACAAAACTTCCAATAATTCAGCAACTAA	1F, S2B
9	FVIII- cons - ideal#1 nick1 BOT	/56-FAM/TTAGTTGCTGAATTATTGGAAGTTTTGTTTTTTTTT	1F, S2B
10	FVIII- cons - ideal#1 nick2 BOT	/5Phos/AACAAAGAGATGCTAACTTTGAATAAGGCATTcacagctgcgctagca	1F, S2B
72	FVIII-NAT#2TOP-FAM	/56-FAM/TGCTAGCGCAGCTGTGAATGCCTTATTCAAAGTTAGCATCTCTTTGTTAAAGACAAACAAAACTTCCAATAATTCAGCAACTAA/3Phos/	S1D, S10D
73	FVIII-NAT#2BOT-TYE665	/5TYE665/TTAGTTGCTGAATTATTGGAAGTTTTGTTTGTCTTTAACAAAGAGATGCTAACTTTGAATAAGGCATTCACAGCTGCGCTAGCA/3Phos/	S1D, S10D
74	FVIII-NAT#2BOTN1-TYE665	/5TYE665/TTAGTTGCTGAATTATTGGAAGTTTTGTTTGTCTTT	S1D, S10D
75	FVIII-NAT#2BOTN2	/5Phos/AACAAAGAGATGCTAACTTTGAATAAGGCATTCACAGCTGCGCTAGCA/3Phos/	S1D, S10D
71	AT TOP	GTGATGCATTGTTTCCGCATCATGCGTCCTTAAAAAGG/3Phos/	S1E
77	AT BOT-full	/56-FAM/TCTTTTTTTCCTTTTTAAGGACGCATGATGCGGAAACAATGCATCAC/3Phos/	S1E, S1F
81	AT_Mimic TOP	ATGCCTTATTCAAAGTTAGCATCTCTTTGTTAAAAAAA/3Phos/	S1E
82	AT_Mimic BOT	/56-FAM/AGTTTTGTTTTTTTTTAACAAAGAGATGCTAACTTTGAATAAGGCAT/3Phos/	S1E, S1F
76	AT TOP-full	GTGATGCATTGTTTCCGCATCATGCGTCCTTAAAAAGGAAAAAAAGA/3Phos/	S1F

ID	Name	Sequence	Figure
78	AT BOT-N1	/56-FAM/tCTTTTTTTCCTTTTT	S1F
79	AT BOT-N2	/5Phos/AAGGACGCATGATGCGGAAACAATGCATCAC/3Phos/	S1F
80	AT_Mimic TOP-full	ATGCCTTATTCAAAGTTAGCATCTCTTTGTTAAAAAAAAACAAAACT/3Phos/	S1F
83	AT_Mimic BOT-N1	/56-FAM/AGTTTTGTTTTTTTTT	S1F
84	AT_Mimic BOT-N2	/5Phos/AACAAAGAGATGCTAACTTTGAATAAGGCAT/3Phos/	S1F
39	FVIII- cons - ideal#2 FAM-TOP	/56-FAM/tgctagcgcagctgtgAATGCCTTATTCAAAGTTAGCATCTCTTTGTTAAAAAAAAACAAAACTTCCAATAATTCAGCAACTAA/3Phos/	3A, 3B
40	FVIII- cons - ideal#2 nick1 Cy5-BOT	/5Cy5/TTAGTTGCTGAATTATTGGAAGTTTTGTTTTTTTT	3A, 3B
41	FVIII- cons - ideal#2 nick2 BOT	/5Phos/AACAAAGAGATGCTAACTTTGAATAAGGCATTcacagctgcgctagca/3Phos/	3A, 3B
56	Dideoxy. Seq. Primer	/56-FAM/tgctagcgcagctgtgAATGC	S10C
57	FVIII- cons - ideal#2 full BOT	TTAGTTGCTGAATTATTGGAAGTTTTGTTTTTTTTAACAAAGAGATGCTAACTTTGAATAAGGCATTcacagctgcgctagca/3Phos/	S10C
85	F8-iv3TOP-link-BOT	/5TYE665/AATGCCTTATTCAAAGTTAGCATCTCTTTGTTAAAAAAAAACAAAACTTCCAATAATTCAGCAAGA/iFluorT/ATTGCTGAATTATTGGAAGTTTTGTTTTTTTTT	S10A
86	F8-iv3BOTN2	/5Phos/AACAAAGAGATGCTAACTTTGAATAAGGCATT/3Phos/	S10A
87	F8-iv3TOP-cy5	/5TYE665/AATGCCTTATTCAAAGTTAGCATCTCTTTGTTAAAAAAAAACAAAACTTCCAATAATTCAGCAA/3Phos/	S10A
88	F8-iv3BOTN1-fam	/56-FAM/TTGCTGAATTATTGGAAGTTTTGTTTTTTTTT	S10A
	A30-RV	rArArArArArArArArArGrArUrArUrCrArArArArArArArArArArArArArArA	S10B
	pA30_Alu5-RV	rgrgrCrCrGrGrGrCrGrGrArUrArUrCrArArArArArArArArArArArArArArA	S10B
O00370	*H. sapiens* (O00370)	MTGSNSHITILTLNVNGLNSPIKRHRLASWIKSQDPSVCCIQETHLTCRDTHRLKIKGWRKIYQANGKQKKAGVAILVSDKTDFKPTKIKRDKEGHYIMVKGSIQQEELTILNIYAPNTGAPRFIKQVLSDLQRDLDSHTLIMGDFNTPLSILDRSTRQKVNKDTQELNSALHQTDLIDIYRTLHPKSTEYTFFSAPHHTYSKIDHIVGSKALLSKCKRTEIITNYLSDHSAIKLELRIKNLTQSRSTTWKLNNLLLNDYWVHNEMKAEIKMFFETNENKDTTYQNLWDAFKAVCRGKFIALNAYKRKQERSKIDTLTSQLKELEKQEQTHSKASRRQEITKIRAELKEIETQKTLQKINESRSWFFERINKIDRPLARLIKKKREKNQIDTIKNDKGDITTDPTEIQTTIREYYKHLYANKLENLEEMDTFLDTYTLPRLNQEEVESLNRPITGSEIVAIINSLPTKKSPGPDGFTAEFYQRYKEELVPFLLKLFQSIEKEGILPNSFYEASIILIPKPGRDTTKKENFRPISLMNIDAKILNKILANRIQQHIKKLIHHDQVGFIPGMQGWFNIRKSINVIQHINRAKDKNHVIISIDAEKAFDKIQQPFMLKTLNKLGIDGMYLKIIRAIYDKPTANIILNGQKLEAFPLKTGTRQGCPLSPLLFNIVLEVLARAIRQEKEIKGIQLGKEEVKLSLFADDMIVYLENPIVSAQNLLKLISNFSKVSGYKINVQKSQAFLYNNNRQTESQIMGELPFTIASKRIKYLGIQLTRDVKDLFKENYKPLLKEIKEDTNKWKNIPCSWVGRINIVKMAILPKVIYRFNAIPIKLPMTFFTELEKTTLKFIWNQKRARIAKSILSQKNKAGGITLPDFKLYYKATVTKTAWYWYQNRDIDQWNRTEPSEIMPHIYNYLIFDKPEKNKQWGKDSLLNKWCWENWLAICRKLKLDPFLTPYTKINSRWIKDLNVKPKTIKTLEENLGITIQDIGVGKDFMSKTPKAMATKDKIDKWDLIKLKSFCTAKETTIRVNRQPTTWEKIFATYSSDKGLISRIYNELKQIYKKKTNNPIKKWAKDMNRHFSKEDIYAAKKHMKKCSSSLAIREMQIKTTMRYHLTPVRMAIIKKSGNNRCWRGCGEIGTLVHCWWDCKLVQPLWKSVWRFLRDLELEIPFDPAIPLLGIYPKDYKSCCYKDTCTRMFIAALFTIAKTWNQPNCPTMIDWIKKMWHIYTMEYYAAIKNDEFISFVGTWMKLETIILSKLSQEQKTKHRIFSLIGGN	S12E, S13D
P11369	*M. musculus* (P11369)	MPTLTTKIKGSNNYFSLISLNINGLNSPIKRHRLTDWLHKQDPTFCCLQETHLREKDRHYLRVKGWKTIFQANGLKKQAGVAILISDKIDFQPKVIKKDKEGHFILIKGKILQEELSILNIYAPNARAATFIRDTLVKLKAYIAPHTIIVGDFNTPLSSKDRSWKQKLNRDTVKLTEVMKQMDLTDIYRTFYPKTKGYTFFSAPHGTFSKIDHIIGHKTGLNRYKNIEIVPCILSDHHGLRLIFNNNINNGKPTFTWKLNNTLLNDTLVKEGIKKEIKDFLEFNENEATTYPNLWDTMKAFLRGKLIALSASKKKRETAHTSSLTTHLKALEKKEANSPKRSRRQEIIKLRGEINQVETRRTIQRINQTRSWFFEKINKIDKPLARLTKGHRDKILINKIRNEKGDITTDPEEIQNTIRSFYKRLYSTKLENLDEMDKFLDRYQVPKLNQDQVDHLNSPISPKEIEAVINSLPTKKSPGPDGFSAEFYQTFKEDLIPILHKLFHKIEVEGTLPNSFYEATITLIPKPQKDPTKIENFRPISLMNIDAKILNKILANRIQEHIKAIIHPDQVGFIPGMQGWFNIRKSINVIHYINKLKDKNHMIISLDAEKAFDKIQHPFMIKVLERSGIQGPYLNMIKAIYSKPVANIKVNGEKLEAIPLKSGTRQGCPLSPYLFNIVLEVLARAIRQQKEIKGIQIGKEEVKISLLADDMIVYISDPKNSTRELLNLINSFGEVVGYKINSNKSMAFLYTKNKQAEKEIRETTPFSIVTNNIKYLGVTLTKEVKDLYDKNFKSLKKEIKEDLRRWKDLPCSWIGRINIVKMAILPKAIYRFNAIPIKIPTQFFNELEGAICKFVWNNKKPRIAKSLLKDKRTSGGITMPDLKLYYRAIVIKTAWYWYRDRQVDQWNRIEDPEMNPHTYGHLIFDKGAKTIQWKKDSIFNNWCWHNWLLSCRRMRIDPYLSPCTKVKSKWIKELHIKPETLKLIEEKVGKSLEDMGTGEKFLNRTAMACAVRSRIDKWDLMKLQSFCKAKDTVNKTKRPPTDWERIFTYPKSDRGLISNIYKELKKVDFRKSNNPIKKWGSELNKEFSPEEYRMAEKHLKKCSTSLIIREMQIKTTLRFHLTPVRMAKIKNSGDSRCWRGCGERGTLLHCWWECRLVQPLWKSVWRFLRKLDIVLPEDPAIPLLGIYPEDAPTGKKDTCSTMFIAALFIIARSWKEPRCPSTEEWIQKMWYIYTMEYYSAIKKNEFMKFLAKWMDLEGIILSEVTHSQRNSHNMYSLISGY	S12E, S13D
O62658	C. familiaris (O62658)	MMTLNSYLSIVTLNVNGLNDPIKRRRVSDWIKKQDPSICCLQETHFRQKDTYSLKIKGWRTIYHSNGPQKKAGVAILISDKLKFTPKTVVRDEEGHYIILKGSIQQEDLTILNIYAPNVGAAKYINQLLTKVKKYLDNNTLILGDFNLALSILDRSSKHNISKETRALNDTLDQMDFTDIYRTLHPNSTEYTFFSSAHGTFSRIDHILGHQSGLNRYQKIGIVPCIFSDHNALKLELNHNKKFGRTSNTWRLRTILLKDERVNQEIKEELKRFMETNENEDTTVQNLWDAAKAVLRGKYIAIQASIQKLERTQIQKLTLHIKELEKKQQMDPTPRRRRELIKIRAELNEIETRRTVEQINRTRSWFFERINKIDKPLASLLKKKREKTQINKIMNEKGEITTNTKEIQTILKTYYEQVYANKLGNLEEMDAFLESHKLPKLEQEEIENLHRPITREEIEAVIKNLPRHKSPGPDGFPGEFYQTFKEEIIPILLKLFGKIERDGVLPNSFYEASITLIPKPDKDPTKKENYRPISLMNMDAKILNKILANRIQQYIKKIIHHDQVGFIPGTQGWFNTRKTINVIHHISKRKTKNHMILSLDAEKAFDKIQHPFLIKTLQSVGIEGTFLDILKAIYEKPTANIILNGEALGAFPLRSGTRQGCPLSPLLFNIVVEVLASAIRQQKDFRGIQFGKEEVKLSLFADEMILYIENPKVSTPRLLQLMQHCGSVAGYIINAQKSVAFLYTNNETEEREIKESIPFTIAPKSIRYLGINLTREVKDLYPQNYRTLLKEIEEDTKRWKNIPCSWIGRINIVKMSMLPRAIYTFNAIPIKIPWTFFKELEQIILKFVWNQKKPRMAGGILGKKTMSGGISMPDFRLYYKAVVIKTVWYWHKNRHIDQWNRIENPEVDPELYGQLIFDKGGKTIHWKKDSLFNKWCWENWTSTCRRMELDPSLSPYTKMNSKWMKDLNVRPDSIQILEENTGHTLFEIGHRNFLQDTSTKAKETKAKMNYWDFIKIRSFCTAKDTVNKTKRQPTEWEKIFANDVSDKGLVSKIYKELIKLNTKETNNPIMKWAKDMNRNFTEGDIDMANMHMRKCFASIAIREIQIKTTMRYHLTPVRMGKINKAGNHKCWRGCGEKGTLLHCWWECELVQPLWKTVWRFLKQLKIYLPYDPAIALLGIYPKDTNAMKRRDTCTPMFIAAMATIAKLWKEPRCPTKDEWIKKMWFMYTMEYYSAISNDRYPPFASTWMELEGIMLSEVSQSEKDKHYMFSFIWGI	S12E, S13D
Q1L835	*B. taurus* (Q1L835)	MATGTYLSVITLNVNGLNAPTKRQRLAEWIQKQDPYICCLQETHLKTGDTYRLKVKGWKKIFHANRDQKKAGVAILISDKIDFKTKAVKRDKEGHYIMIKGSIQEEEITIINIYAPNTGAPQYVRQMLTSMKGEINNNTIIVGDFNTPLTPMDRSTKQKINKETQTLNDTIDQLDLIDIYRSFHPKTMNFTFFSSAHGTFSRIDHILGHKASLGKFKKIEIIPSIFSDHNAVRLDLNYRRKTIKNSNIWRLNNTLLNNQQITEEIKKEIKICIETNENENTTTQNLWDAVKAVLRGKFIAIQAHLKKQEKSQINNLTLHLKQLEKEEMKNPRVSRRKEILKIRAEINAKETKETIAKINKTKSWFFERINKIDKPLARLIKKQREKNQINKIRNENGEITTDNTEIQRIIRDYYQQLYANKMDNVEEMDKFLEKYNFPKLDQEEIENLNRPITTMEIETVIKNLSANKSPGPDGFTAEFYRKFREELTPILLKLFQKIAEEGKLPNSFYEATITLIPKPDKDPTKKENYRPISLMNIDAKILNKILAIRIQQHIKKIIHHDQVGFIPGMQGFFNIRKSINVIHHINKLKNKNHMIISIDAEKAFDKIQHPFMIKTLQKAGIEGTYLNIIKAIYDKPTANIILNGEKLKAFPLKSGTRQGCPLSPLLFNIVLEVLATAIRAEKEIKGIQIGKEEVKLSLFADDMILYIENPKDSTRKLLEIINDYSKVAGYKINTQKSLAFLYTNNEKTEREIKETIPFTIATERIKYLGIYLPKETKDLYLENYKTLVKEIKEDTNRWRNIPCSWIGRINIVKMSILPKAIYRFNAIPIKLPTVFFTELEQIISQFIWKYKKPRIAKAILRKKNGAGGINLPDFRLYYRATVIKTVWYWHKDRNMDQWNKIESPEINPRTYGHLIFDKGGKDIQWIKDNLFNKWCWEIWSTTCKRMKLDHFLTPYTKINSKWIKDLNVRPETIKLLEENIGKTLSDIYHSRILYDPPPRIMEIKAKINKWDLINLKSFCTSKETISKVKRQPSEWEKIIANEATDKQLISKIYKRLLQLNSRKINDPIKKWAKELNRHFSKKDIQMANKHMKRCSTSLIIREMQIKTTMRYHFTPVRMAAIQKSTNNKCWRGCGEKGTLLHCWWECKLVQPLWRTVWRFLKKLEIEPPYDPAIPLLGIHTEKTRRERDTCTPMFIAALFIIAKTWKQPRCPSADEWIRKLWYIYTMEYYSAIKKNTFESVLMRWMKLEPIIQSEVSQKEKHKYSILTHIYGI	S12E, S13D

ID	Name	Sequence	Figure
A5Z0F6	*S. scrofa* (A5Z0F6)	MAINNHLSIITLNVNGLNAPIKRHRVAEWIKRQKPSICCLQETHLRTKDTYRLKVKGWGKVFHANRHDRKAGVATLISDKIDFKTKDIKKDKEGHYLMIKGSIQGEDVTIINIYA<br>PNIGAPRYIQQILTDIKGDIDENTIIVGDLNTPLTSMDRSSRQKTNKATEILKETIEKLDLIDIFRTLHPKKSEYTFFSNAHGTFSRIDHILGHKANLNKFRSIEIISSIFSDHNAMKLEI<br>NHGKRKEKKPTPWRLNNMLLKNQWVNEEIKKEIKNYLETNDNEDTTSQNLWDAAKAVLRGKFIAIQAFLKKEERSQIDNLTLHLNELEKEEQRSPKVSRRKEIVKIKEEINKIE<br>TQKTIEKINKTKSWFFEKVNKIDKPLARLTKKRRERTQITKIINEKGEITTDTAEIQKTIREYYEQLYGNKFDNLEEMDNFLESYSLPKLNQAEADQLNRPITRNEIEEVIKSLPTNK<br>SPGPDGFTGEFYQTYKEELVPILLKLFQKVEEEGILPKTFYEATITLIPKPGRDTTKKENYRPISLMNIDAKLLNKILANRIQQHIKKIIHHDQVGFIPGSQGWFNIRKSINIIHHINK<br>KKVKNHMIISIDAEKAFDKVQHPFMIKTLAKVGIEGTFLNIIKAIYDKPTANIILNGEKLKAFSLKSGTRQGCPLSPLLFNIVLEVLATAIRQTKEIKGIHIGREEIKLSLYADDMILYL<br>ENPKDSTPKLLELINKFSKVAGYKINIQKSVAFLYTSNEALEKEYKNTIPFKIVPHKIKYLGIHLTKEVKDLYAENYKTLIKEIKEDVKKWKDIPCSWIGKINIVKMAILPKAIYRFNAI<br>PIKLPMTFFTELEQTIQTFIWNNKRPRIAKAILRNKNQAGGITLPDFKKYYKATVIKTVWYWYQNRQTDQWNRIENLEINPDTYGQLIFDKGGKNIKWEKETLFSKHCWETW<br>TAACKAMKLEHTLTPCTKINSKWLKDLNIRQDTIKLLEENIGKTLSDINIMNIFSGQSPKAIEIRAKINPWDLIKLKSFCTAKETQKKTKRQLTEWEKIVSNDATDKGLISRIYKQLI<br>QLNSKKTNQSMEKWAKDLNRHFSKEDIQMANKHMKKCSTSLIIREMQIKTTMRYHLTPVRMAIIHKSTNNKCWRGCGEKGTLLHCWWECKLVQPLWRTVWRYLRNLYI<br>ELPYDPAIPLLGIYPDKALLKRDTCTRMFIAALFTIARTWKQSKCPSTEDWIRKMWYIYTMEYYSAIKKDDIMPFAATWMELENLILSEMSQKDKDKYHMTSLITGI	S12E, S13D
Lizard L1-AC1	*A. carolinensis* (L1-1)	MREIQQKRREHKEMSKVCYQLKCFSNNLNGLNSPNKRNRLFNKLKKEKYNIIAIQETHIALKHISLLKKGYLGQSFIAADKVKKRGVVLYIDDKFPAKEVFKDNEGRVIAVTIDF<br>ENEKILICNIYAPNGPKTKFIKTLREKINNTEFDHMILLGDFNGVLDVKLDKTNSSKKVRETTSLPKNLIVLKEEYDLIDPWRELNPTSKDYTHFSNRHQAWSRIDMIWTSKSLM<br>TTISDIQIRARDISDHCPLTMVINKKHCLKKWRLNENLLKHKQDVNKINAMIVEYFSINDNGETNPQIVWDAFKAVARGFFIQLNSSKRKHKDLEWNRLNKEIEAKEKELKINP<br>KNQKIKRNLALLAKMKERWDLEEVAKKIKWVKQYAFENANKPGKWLARLIRKKKQNRQIMKIKFEERMVNSDKEIREAFQKFYVNLYSKDNINPEAITEYLGKQKLDKITDE<br>QRLDLNKEITEEEILKAIKSLDSNKTPGPDGFIAGFYKLGQPEMIKFLKKLMNQALQDKVIPETWKEATIIMIPKEDADLSETKNYRPISLLNTDYKIFTKILANRLKKFLNGWIGE<br>DQTGFLPSRTIKDNVRVILDAMEFYEQHNQREVGFLSVDAEKAFDNLNWNFFKLLFQELDLGTQIQNGINSIYEDQWAKIQINGQETDKIVISKGTRQGCPLSPLIFIMALEIL<br>LKAIKKDINLQGIKIDNQDYKYRAFADDVICIIENPIQNIHKWIAKIDEFGKVAGLKINKKKTMMLTKNMSKRKQKELQDIAGLETPLKLKYLGIWISAKNNQLLDLNYIRIWKEI<br>KNDLEKWKNINVSLLGRIAIIKMNILPKLLYLFQNIPIIRSTKLFKDWQKEIMRFIWKNKRARINYNIMISRKTQGGLGVPDLKLYHDACALCWIRDWAKLEKVRILNLEGHELR<br>KGWHGYMWYKKAKIEKQFGNHFIRSALLRVWDNYKSRFHTKTPLWLSPIEADHRRLLGWRKWPTYRELLTVANDKRIPPKLKELTKIQELNKNVSWYQYFQIKEAYKGDSL<br>VGFEQEPGFWDKFISIDKKCITVMYNNLLSWDTESSRVTKAMTTWAKNIERPIQMQEWESIWNKKMKYTYSVDLKENWLKLIHRWYLTPKKIGLMYKNANNKCWRCKTQ<br>TGSYFHMWWKCKKLGKFWTAILEESNVILKTKFIKKPELLLLGLYNCDDKIDPNTDKLFTFFITAARLVIARLWKSTQIPTKELWLDKLLEIKNMDQLSFLIKKTNGTILKATDWT<br>SFEDYLKNIVHITKD	S12E, S13D
Frog L1-6	*X. tropicalis* (L1-6)	MATYNIMSWNIRGLNSKYKRSLMWIYLKRHSPSILLLQETHLVGQKTLALKKPWVGWTYHASFSTYSRGVSILVRKNLPFELSQIISDHYGRYILIACLLANKPLILANIYMPPPF<br>TTTLLQHIGKKLADLPPAPLCIMGDMNQVMDLTKDRLHSNIQGPTNLAQWANSLGLSDIWRWKHPLEKAYSCHSLPHKSFSRIDIALATADILPLVEQISYLPQHLSDHSPLL<br>LRLQWLPNTIDRLWRLSPLWLKHPDIASSSREAYIEYWEFNTGTAPQGIVWDASKAATRGSLTALISHERKKSKESITKAEEHLAETQRNHFHSPTTDTYEEVRRAEAALARES<br>TIITKKALLYKTQHIFDKGDKNSKILAILAKQQQASIAVSRIQTETGNIVHEPYLIAETFASYYQKLYSSTATYTAPQLRHFLDSIHIPKLSPTERAWLNAPITLEEITTAIQSLPSNKTP<br>GLDGLPPDWYKALNDLVTPQLLTTLQAAWDSQSLPPSFAEALIVVIPKAGRDPTLCSSYRPISLINTDAKILAKVLATRLTRAVQDLIHPDQSGFMPGRATDFNLRRLFTNLQIT<br>HTNSGARAVASLDSEKAFDSIEWEYLWEVLRRFGLGARFIQWLKMLYKHPIARVRVNNTVSPAFSLHRGTRQGCPLSPTLFALAIEPLAITIRNNPNIKGLNFANVTEKVSLFA<br>DDILIYLADSGTSLSTLLETIQTFGKYSGLKVNWDKSQLYHIDPAPVAQAIPNTPLKEVMSFKYLGIQVHTDPNTFRQLNLDPLTDSLSLALKNWEKLPLSLWGRVNIIKMIYLPK<br>LLYILHNTPYAIPRAVFKKLNTIMNPFIWANKPPRISWEKLTSPIGKGGLGLPHFYFYYLTSQIYYLHWCFAPNPYNPNMPLQASILHSLEGLGTYPYRHVSDMTALPDVLKTPH<br>QAWTTALKLLGCPLPYPSPHLPLWKNSLLQQLYDLPDVIYWARLGIKKLSDLLLQDQFPTLQNLQERIPGQRIQLYRYLQLRHAFQAQFHSLSLTQETTPLEEALYSPTPKKLLS<br>NLYKVVMESLPPPFNRARQLWHQAIPNLQEDQWEEAIDTAYDYLISIKDRLIQYKSLHQIYITPLKLLKIGKRQNDLCPRCDTPGANFIHMIWACPPINRFWKDVMGTMAQE<br>LGTPRIIDPVVCLLGVIDDILPTNAARIRFRTLMFYAKKTVIMHWMGNNLPSLNFWRQLVDSALPLIKLTYETRGAMDKFEKVWSDWCNPDPQGQQP	S12E, S13D
Zebrafish L1-1	*D. rerio* (L1-1)	MVKPHNVNASGVCQVNLISWNVKSLNHPVKRGKVLSHLKQLNTDIAFLQETHLKTFDHFRLRGGWVGQLFHSTFHSKSRGTAILISKTVSFEASKIEADPAGRYIMVVGRLN<br>NTPVVMVNVYAPNWDDSAFFTGLFSRIPNIDTHHLILGGDINCVLSPSLDRSSLKPMIPSRTTQVINQLLKTYGMIDVWRFQNPGCRGYSFYSPVHKTYSRIDYFFLDSELLPL<br>VSECKYNAIVISDHAPLLITLDMPITSNNYRPWRFNTLLLSDVEFVKFISSEIREYLVHNQTPGISSSLIWESLKAYLRGQIISYSARLKKKQHERLKKIENDILKLDEILAHSSTPDM<br>FRQRLALQSEFNLLCTKQTENLLIKSRHKMYEHGEKIGKILAHQLRQQNAAHSIMSVNDNTGTKLTNPLEINHRFREYYSQLYTSESCKDESLFDSFFKKISLPTIDQEFALDME<br>NPFSKDEFIRAVSSMQNGKSPGPDGFPSEFFKKFSGDLAPILLSLYEESSVTGSLPETMNQAIISLIYKKDKNPSECSSYRPISLLNVDSKIFAKILAHRLEIVLPTIVSGDQTGFIKN<br>RYSFYNIRRLLNILHHPTPSDVPEVLLSLDAEKAFDRVEWDYLFYTLKKFGFGTKFISWIKILYSSPMAAIRTNCHISPFFSLERGTRQGCPLSPLLFALVIEPLSIAIRNDINIKGIQR<br>DNFEHKISLYADDTLLYISEPLTTLPQIMTLLTAFGKISGYKINMQKSELMPINNAGRKIIFTSLPFKITKDKFKYLGIWITNKYKHLYKVNFPPLIDSIKKDLERWNPLPLSLGGRIN<br>TIKMNILPRCLYLFQCIPVFLTKSFFLLLDKLISSFIWNGKNARIRKNILQRHRDHGGLSLPNIQQYYWAANIRAMLHWSNPSYDSGPNWLSLENTSNFSTSLHALLCSNFPTPE<br>PLSKYSLNPVVKHSLKIWAQFRRSFALKGLSAYAPIARNHMFTPSTIDKTFDIWSMKGLKILKDMFIDGQFASFQQVKVKFQIPNSHFFRYLQLRSFVSSSMSHYPSLPPPSLLD<br>SIMELSPYSKGLIGKIYSIINSHNLEPLVKLKRKWEVELEIELSEDMWQSVLDNIHSSSICLKHRVIQFKVVHRLHWSKVKLAKFKPNIDPNCDRCSIEPATLSHMFWACSKLKKF<br>WQLIFKFLSDALNTYVEPEAIISIFGITPQSLCFNKSKINVIAFATLLARRLILLKWKEKLPPTFKQWLMELLHHLTLEKIRYTLGGCTDMFFLTWQPVLDHVKKMDPSVILEE	S12E, S13D
	ORF2p	MTGSTSHITILTLNINGLNSAIKRHRLASWIKSQDPSVCCIQETHLTCRDTHRLKIKGWRKIYQANGKQKKAGVAILVSDKTDFKPTKIKRDKEGHYIMVKGSIQQEELTILNIYA<br>PNTGAPRFIKQVLSDLQRDLDSHTLIMGDFNTPLSTLDRSTRQKVNKDTQELNSALHQADLIDIYRTLHPKSTEYTFFSAPHHTYSKIDHIVGSKALLSKCKRTEIITNYLSDHSAI<br>KLELRIKNLTQSRSTTWKLNNLLLNDYWVHNEMKAEIKMFFETNENKDTTYQNLWDAFKAVCRGKFIALNAYKRKQERSKIDTLTSQLKELEKQEQTHSKASRRQEITKIRAE<br>LKEIETQKTLQKINESRSWFFERINKIDRPLARLIKKKREKNQIDTIKNDKGDITTDPTEIQTTIREYYKHLYANKLENLEEMDTFLDTYTLPRLNQEEVESLNRPITGSEIVAIINSLP<br>TKKSPGPDGFTAEFYQRYKEELVPFLLKLFQSIEKEGILPNSFYEASIILIPKPGRDTTKKENFRPISLMNIDAKILNKILANRIQQHIKKLIHHDQVGFIPGMQGWFNIRKSINVIQ<br>HINRAKDKNHMIISIDAEKAFDKIQQPFMLKTLNKLGIDGTYFKIIRAIYDKPTANIILNGQKLEAFPLKTGTRQGCPLSPLLFNIVLEVLARAIRQEKEIKGIQLGKEEVKLSLFAD<br>DMIVYLENPIVSAQNLLKLISNFSKVSGYKINVQKSQAFLYTNNRQTESQIMGELPFTIASKRIKYLGIQLTRDVKDLFKENYKPLLKEIKEETNKWKNIPCSWVGRINIVKMAILP<br>KVIYRFNAIPIKLPMTFFTELEKTTLKFIWNQKRARIAKSILSQKNKAGGITLPDFKLYYKATVTKTAWYWYQNRDIDQWNRTEPSEIMPHIYNYLIFDKPEKNKQWGKDSLFN<br>KWCWENWLAICRKLKLDPFLTPYTKINSRWIKDLNVKPKTIKTLEENLGITIQDIGVGKDFMSKTPKAMATKDKIDKWDLIKLKSFCTAKETTIRVNRQPTTWEKIFATYSSDK<br>GLISRIYNELKQIYKKKTNNPIKKWAKDMNRHFSKEDIYAAKKHMKKCSSSLAIREMQIKTTMRYHLTPVRMAIIKKSGNNRCWRGCGEIGTLLHCWWDCKLVQPLWKSV<br>WRFLRDLELEIPFDPAIPLLGIYPNEYKSCCYKDTCTRMFIAALFTIAKTWNQPKCPTMIDWIKKMWHIYTMEYYAAIKNDEFISFVGTWMKLETIILSKLSQEQKTKHRIFSLIG<br>GN	AlphaFold3 4A, 4E, S12, S13
UniProt P12004	PCNA	MFEARLVQGSILKKVLEALKDLINEACWDISSSGVNLQSMDSSHVSLVQLTLRSEGFDTYRCDRNLAMGVNLTSMSKILKCAGNEDIITLRAEDNADTLALVFEAPNQEKVSD<br>YEMKLMDLDVEQLGIPEQEYSCVVKMPSGEFARICRDLSHIGDAVVISCAKDGVKFSASGELGNGNIKLSQTSNVDKEEEAVTIEMNEPVQLTFALRYLNFFTKATPLSSTVTL<br>SMSADVPLVVEYKIADMGHLKYYLAPKIEDEEGS	AlphaFold3 4A, S12
	DNA (1st primer top)	AATGCCTTATTCAAAGTTAGCATCTCTTTGTTAAAAAAA	AlphaFold3 4A, S12
	DNA (1st primer bot.)	AACAAAGAGATGCTAACTTTGAATAAGGCATT	AlphaFold3 4A, S12
UniProt P11940	PABPC1 (1-189)	MNPSAPSYPMASLYVGDLHPDVTEAMLYEKFSPAGPILSIRVCRDMITRRSLGYAYVNFQQPADAERALDTMNFDVIKGKPVRIMWSQRDPSLRKSGVGNIFIKNLDKSID<br>NKALYDTFSAFGNILSCKVVCDENGSKGYGFVHFETQEAAERAIEKMNGMLLNDRKVFVGRFKSRKEREAELGARAKE	AlphaFold3 4E, S13
	RNA (23nt)	AAAAAAAAAAAAAAAAAAAAAAA	AlphaFold3 4E, S13

Movie S1.

Morph of volumes along the first component from 3D variability analysis in CryoSPARC. Volumes are colored according to domains and regions, as in Fig. 1.

Movie S2.

Morph of volumes along the second component from 3D variability analysis in CryoSPARC. Volumes are colored according to domains and regions, as in Fig. 1.

Movie S3.

Morph of volumes along the third component from 3D variability analysis in CryoSPARC. Volumes are colored according to domains and regions, as in Fig. 1.

Data S1.

Results of the AlphaFold3 predictions. Data S1 includes AlphaFold3 prediction outputs, ChimeraX sessions, and PAE plots for ORF2p and host factors reported in Taylor *et al*., eLife, 2018.

References and Notes

1. J. D. Boeke, D. J. Garfinkel, C. A. Styles, G. R. Fink, Ty elements transpose through an RNA intermediate. *Cell* **40**, 491–500 (1985). doi:10.1016/0092-8674(85)90197-7 Medline

2. International Human Genome Sequencing Consortium, Initial sequencing and analysis of the human genome. *Nature* **409**, 860–921 (2001). doi:10.1038/35057062 Medline

3. B. Brouha, J. Schustak, R. M. Badge, S. Lutz-Prigge, A. H. Farley, J. V. Moran, H. H. Kazazian Jr., Hot L1s account for the bulk of retrotransposition in the human population. *Proc. Natl. Acad. Sci. U.S.A.* **100**, 5280–5285 (2003). doi:10.1073/pnas.0831042100 Medline

4. C. R. Beck, P. Collier, C. Macfarlane, M. Malig, J. M. Kidd, E. E. Eichler, R. M. Badge, J. V. Moran, LINE-1 retrotransposition activity in human genomes. *Cell* **141**, 1159–1170 (2010). doi:10.1016/j.cell.2010.05.021 Medline

5. D. M. Sassaman, B. A. Dombroski, J. V. Moran, M. L. Kimberland, T. P. Naas, R. J. DeBerardinis, A. Gabriel, G. D. Swergold, H. H. Kazazian Jr., Many human L1 elements are capable of retrotransposition. *Nat. Genet.* **16**, 37–43 (1997). doi:10.1038/ng0597-37 Medline

6. J. V. Moran, S. E. Holmes, T. P. Naas, R. J. DeBerardinis, J. D. Boeke, H. H. Kazazian Jr., High frequency retrotransposition in cultured mammalian cells. *Cell* **87**, 917–927 (1996). doi:10.1016/S0092-8674(00)81998-4 Medline

7. D. C. Hancks, H. H. Kazazian Jr., Roles for retrotransposon insertions in human disease. *Mob. DNA* **7**, 9 (2016). doi:10.1186/s13100-016-0065-9 Medline

8. K. H. Burns, Transposable elements in cancer. *Nat. Rev. Cancer* **17**, 415–424 (2017). doi:10.1038/nrc.2017.35 Medline

9. V. Gorbunova, A. Seluanov, P. Mita, W. McKerrow, D. Fenyö, J. D. Boeke, S. B. Linker, F. H. Gage, J. A. Kreiling, A. P. Petrashen, T. A. Woodham, J. R. Taylor, S. L. Helfand, J. M. Sedivy, The role of retrotransposable elements in ageing and age-associated diseases. *Nature* **596**, 43–53 (2021). doi:10.1038/s41586-021-03542-y Medline

10. P. J. Enyeart, G. Mohr, A. D. Ellington, A. M. Lambowitz, Biotechnological applications of mobile group II introns and their reverse transcriptases: Gene targeting, RNA-seq, and non-coding RNA analysis. *Mob. DNA* **5**, 2 (2014). doi:10.1186/1759-8753-5-2 Medline

11. J. Strecker, A. Ladha, Z. Gardner, J. L. Schmid-Burgk, K. S. Makarova, E. V. Koonin, F. Zhang, RNA-guided DNA insertion with CRISPR-associated transposases. *Science* **365**, 48–53 (2019). doi:10.1126/science.aax9181 Medline

12. G. D. Lampe, R. T. King, T. S. Halpin-Healy, S. E. Klompe, M. I. Hogan, P. L. H. Vo, S. Tang, A. Chavez, S. H. Sternberg, Targeted DNA integration in human cells without double-strand breaks using CRISPR-associated transposases. *Nat. Biotechnol.* **42**, 87–98 (2024). doi:10.1038/s41587-023-01748-1 Medline

13. S. E. Klompe, P. L. H. Vo, T. S. Halpin-Healy, S. H. Sternberg, Transposon-encoded CRISPR-Cas systems direct RNA-guided DNA integration. *Nature* **571**, 219–225 (2019). doi:10.1038/s41586-019-1323-z Medline

14. S. C. Pimentel, H. E. Upton, K. Collins, Separable structural requirements for cDNA synthesis, nontemplated extension, and template jumping by a non-LTR retroelement reverse transcriptase. *J. Biol. Chem.* **298**, 101624 (2022). doi:10.1016/j.jbc.2022.101624 Medline

15. X. Zhang, B. V. Treeck, C. A. Horton, J. J. R. McIntyre, S. M. Palm, J. L. Shumate, K. Collins, Harnessing eukaryotic retroelement proteins for transgene insertion into human safe-harbor loci. *Nat. Biotechnol.* **43**, 42–51 (2025). doi:10.1038/s41587-024-02137-y Medline

16. D. A. Kulpa, J. V. Moran, Cis-preferential LINE-1 reverse transcriptase activity in ribonucleoprotein particles. *Nat. Struct. Mol. Biol.* **13**, 655–660 (2006). doi:10.1038/nsmb1107 Medline

17. D. A. Kulpa, J. V. Moran, Ribonucleoprotein particle formation is necessary but not sufficient for LINE-1 retrotransposition. *Hum. Mol. Genet.* **14**, 3237–3248 (2005). doi:10.1093/hmg/ddi354 Medline

18. S. L. Martin, M. Cruceanu, D. Branciforte, P. Wai-Lun Li, S. C. Kwok, R. S. Hodges, M. C. Williams, LINE-1 retrotransposition requires the nucleic acid chaperone activity of the ORF1 protein. *J. Mol. Biol.* **348**, 549–561 (2005). doi:10.1016/j.jmb.2005.03.003 Medline

19. Q. Feng, J. V. Moran, H. H. Kazazian Jr., J. D. Boeke, Human L1 retrotransposon encodes a conserved endonuclease required for retrotransposition. *Cell* **87**, 905–916 (1996). doi:10.1016/S0092-8674(00)81997-2 Medline

20. S. L. Mathias, A. F. Scott, H. H. Kazazian Jr., J. D. Boeke, A. Gabriel, Reverse transcriptase encoded by a human transposable element. *Science* **254**, 1808–1810 (1991). doi:10.1126/science.1722352 Medline

21. D. D. Luan, M. H. Korman, J. L. Jakubczak, T. H. Eickbush, Reverse transcription of R2Bm RNA is primed by a nick at the chromosomal target site: A mechanism for non-LTR retrotransposition. *Cell* **72**, 595–605 (1993). doi:10.1016/0092-8674(93)90078-5 Medline

22. J. Jurka, Sequence patterns indicate an enzymatic involvement in integration of mammalian retroposons. *Proc. Natl. Acad. Sci. U.S.A.* **94**, 1872–1877 (1997). doi:10.1073/pnas.94.5.1872 Medline

23. S. T. Szak, O. K. Pickeral, W. Makalowski, M. S. Boguski, D. Landsman, J. D. Boeke, Molecular archeology of L1 insertions in the human genome. *Genome Biol.* **3**, research0052.1 (2002). doi:10.1186/gb-2002-3-10-research0052 Medline

24. K. K. Kojima, Different integration site structures between L1 protein-mediated retrotransposition in *cis* and retrotransposition in *trans*. *Mob. DNA* **1**, 17 (2010). doi:10.1186/1759-8753-1-17 Medline

25. E. T. Baldwin, T. van Eeuwen, D. Hoyos, A. Zalevsky, E. P. Tchesnokov, R. Sánchez, B. D. Miller, L. H. Di Stefano, F. X. Ruiz, M. Hancock, E. Işik, C. Mendez-Dorantes, T. Walpole, C. Nichols, P. Wan, K. Riento, R. Halls-Kass, M. Augustin, A. Lammens, A. Jestel, P. Upla, K. Xibinaku, S. Congreve, M. Hennink, K. B. Rogala, A. M. Schneider, J. E. Fairman, S. M. Christensen, B. Desrosiers, G. S. Bisacchi, O. L. Saunders, N. Hafeez, W. Miao, R. Kapeller, D. M. Zaller, A. Sali, O. Weichenrieder, K. H. Burns, M. Götte, M. P. Rout, E. Arnold, B. D. Greenbaum, D. L. Romero, J. LaCava, M. S. Taylor,

Structures, functions and adaptations of the human LINE-1 ORF2 protein. *Nature* **626**, 194–206 (2024). doi:10.1038/s41586-023-06947-z Medline

26. A. Thawani, A. J. F. Ariza, E. Nogales, K. Collins, Template and target-site recognition by human LINE-1 in retrotransposition. *Nature* **626**, 186–193 (2024). doi:10.1038/s41586-023-06933-5 Medline
27. H. H. Kazazian Jr., C. Wong, H. Youssoufian, A. F. Scott, D. G. Phillips, S. E. Antonarakis, Haemophilia A resulting from *de novo* insertion of L*1* sequences represents a novel mechanism for mutation in man. *Nature* **332**, 164–166 (1988). doi:10.1038/332164a0 Medline
28. G. J. Cost, Q. Feng, A. Jacquier, J. D. Boeke, Human L1 element target-primed reverse transcription *in vitro*. *EMBO J.* **21**, 5899–5910 (2002). doi:10.1093/emboj/cdf592 Medline
29. S. Viollet, C. Monot, G. Cristofari, L1 retrotransposition: The snap-velcro model and its consequences. *Mob. Genet. Elements* **4**, e28907 (2014). doi:10.4161/mge.28907 Medline
30. C. Monot, M. Kuciak, S. Viollet, A. A. Mir, C. Gabus, J.-L. Darlix, G. Cristofari, The specificity and flexibility of l1 reverse transcription priming at imperfect T-tracts. *PLOS Genet.* **9**, e1003499 (2013). doi:10.1371/journal.pgen.1003499 Medline
31. R. Craigie, K. Mizuuchi, Transposition of Mu DNA: Joining of Mu to target DNA can be uncoupled from cleavage at the ends of Mu. *Cell* **51**, 493–501 (1987). doi:10.1016/0092-8674(87)90645-3 Medline
32. H. Savilahti, P. A. Rice, K. Mizuuchi, The phage Mu transpososome core: DNA requirements for assembly and function. *EMBO J.* **14**, 4893–4903 (1995). doi:10.1002/j.1460-2075.1995.tb00170.x Medline
33. T. Fanning, M. Singer, The LINE-1 DNA sequences in four mammalian orders predict proteins that conserve homologies to retrovirus proteins. *Nucleic Acids Res.* **15**, 2251–2260 (1987). doi:10.1093/nar/15.5.2251 Medline
34. A. M. Lentzsch, J. L. Stamos, J. Yao, R. Russell, A. M. Lambowitz, Structural basis for template switching by a group II intron–encoded non-LTR-retroelement reverse transcriptase. *J. Biol. Chem.* **297**, 100971 (2021). doi:10.1016/j.jbc.2021.100971 Medline
35. M. E. Wilkinson, C. J. Frangieh, R. K. Macrae, F. Zhang, Structure of the R2 non-LTR retrotransposon initiating target-primed reverse transcription. *Science* **380**, 301–308 (2023). doi:10.1126/science.adg7883 Medline
36. G. E. Ghanim, A. J. Fountain, A.-M. M. van Roon, R. Rangan, R. Das, K. Collins, T. H. D. Nguyen, Structure of human telomerase holoenzyme with bound telomeric DNA. *Nature* **593**, 449–453 (2021). doi:10.1038/s41586-021-03415-4 Medline
37. T. A. Steitz, DNA polymerases: Structural diversity and common mechanisms. *J. Biol. Chem.* **274**, 17395–17398 (1999). doi:10.1074/jbc.274.25.17395 Medline
38. O. Piskareva, C. Ernst, N. Higgins, V. Schmatchenko, The carboxy-terminal segment of the human LINE-1 ORF2 protein is involved in RNA binding. *FEBS Open Bio* **3**, 433–437 (2013). doi:10.1016/j.fob.2013.09.005 Medline

39. V. Arinkin, G. Smyshlyaev, O. Barabas, Jump ahead with a twist: DNA acrobatics drive transposition forward. *Curr. Opin. Struct. Biol.* **59**, 168–177 (2019). doi:10.1016/j.sbi.2019.08.006 Medline

40. J. Y. Wang, P. Pausch, J. A. Doudna, Structural biology of CRISPR-Cas immunity and genome editing enzymes. *Nat. Rev. Microbiol.* **20**, 641–656 (2022). doi:10.1038/s41579-022-00739-4 Medline

41. I. Miller, M. Totrov, L. Korotchkina, D. N. Kazyulkin, A. V. Gudkov, S. Korolev, Structural dissection of sequence recognition and catalytic mechanism of human LINE-1 endonuclease. *Nucleic Acids Res.* **49**, 11350–11366 (2021). doi:10.1093/nar/gkab826 Medline

42. D. E. Symer, C. Connelly, S. T. Szak, E. M. Caputo, G. J. Cost, G. Parmigiani, J. D. Boeke, Human l1 retrotransposition is associated with genetic instability in vivo. *Cell* **110**, 327–338 (2002). doi:10.1016/S0092-8674(02)00839-5 Medline

43. N. Zingler, U. Willhoeft, H.-P. Brose, V. Schoder, T. Jahns, K.-M. O. Hanschmann, T. A. Morrish, J. Löwer, G. G. Schumann, Analysis of 5′ junctions of human LINE-1 and Alu retrotransposons suggests an alternative model for 5′-end attachment requiring microhomology-mediated end-joining. *Genome Res.* **15**, 780–789 (2005). doi:10.1101/gr.3421505 Medline

44. E. M. Ostertag, H. H. Kazazian Jr., Twin priming: A proposed mechanism for the creation of inversions in L1 retrotransposition. *Genome Res.* **11**, 2059–2065 (2001). doi:10.1101/gr.205701 Medline

45. N. Gilbert, S. Lutz-Prigge, J. V. Moran, Genomic deletions created upon LINE-1 retrotransposition. *Cell* **110**, 315–325 (2002). doi:10.1016/S0092-8674(02)00828-0 Medline

46. J. S. Myers, B. J. Vincent, H. Udall, W. S. Watkins, T. A. Morrish, G. E. Kilroy, G. D. Swergold, J. Henke, L. Henke, J. V. Moran, L. B. Jorde, M. A. Batzer, A comprehensive analysis of recently integrated human Ta L1 elements. *Am. J. Hum. Genet.* **71**, 312–326 (2002). doi:10.1086/341718 Medline

47. N. Gilbert, S. Lutz, T. A. Morrish, J. V. Moran, Multiple fates of L1 retrotransposition intermediates in cultured human cells. *Mol. Cell. Biol.* **25**, 7780–7795 (2005). doi:10.1128/MCB.25.17.7780-7795.2005 Medline

48. D. Kimanius, K. Jamali, M. E. Wilkinson, S. Lövestam, V. Velazhahan, T. Nakane, S. H. W. Scheres, Data-driven regularization lowers the size barrier of cryo-EM structure determination. *Nat. Methods* **21**, 1216–1221 (2024). doi:10.1038/s41592-024-02304-8 Medline

49. O. Weichenrieder, K. Repanas, A. Perrakis, Crystal structure of the targeting endonuclease of the human LINE-1 retrotransposon. *Structure* **12**, 975–986 (2004). doi:10.1016/j.str.2004.04.011 Medline

50. E. M. Adney, M. T. Ochmann, S. Sil, D. M. Truong, P. Mita, X. Wang, D. J. Kahler, D. Fenyö, L. J. Holt, J. D. Boeke, Comprehensive Scanning Mutagenesis of Human Retrotransposon LINE-1 Identifies Motifs Essential for Function. *Genetics* **213**, 1401–1414 (2019). doi:10.1534/genetics.119.302601 Medline

51. T. A. Morrish, N. Gilbert, J. S. Myers, B. J. Vincent, T. D. Stamato, G. E. Taccioli, M. A. Batzer, J. V. Moran, DNA repair mediated by endonuclease-independent LINE-1 retrotransposition. *Nat. Genet.* **31**, 159–165 (2002). doi:10.1038/ng898 Medline

52. A. J. Doucet, J. E. Wilusz, T. Miyoshi, Y. Liu, J. V. Moran, A 3′ Poly(A) Tract Is Required for LINE-1 Retrotransposition. *Mol. Cell* **60**, 728–741 (2015). doi:10.1016/j.molcel.2015.10.012 Medline

53. L. Dai, M. S. Taylor, K. A. O'Donnell, J. D. Boeke, Poly(A) binding protein C1 is essential for efficient L1 retrotransposition and affects L1 RNP formation. *Mol. Cell. Biol.* **32**, 4323–4336 (2012). doi:10.1128/MCB.06785-11 Medline

54. M. S. Taylor, J. LaCava, P. Mita, K. R. Molloy, C. R. L. Huang, D. Li, E. M. Adney, H. Jiang, K. H. Burns, B. T. Chait, M. P. Rout, J. D. Boeke, L. Dai, Affinity proteomics reveals human host factors implicated in discrete stages of LINE-1 retrotransposition. *Cell* **155**, 1034–1048 (2013). doi:10.1016/j.cell.2013.10.021 Medline

55. M. S. Taylor, I. Altukhov, K. R. Molloy, P. Mita, H. Jiang, E. M. Adney, A. Wudzinska, S. Badri, D. Ischenko, G. Eng, K. H. Burns, D. Fenyö, B. T. Chait, D. Alexeev, M. P. Rout, J. D. Boeke, J. LaCava, Dissection of affinity captured LINE-1 macromolecular complexes. *eLife* **7**, e30094 (2018). doi:10.7554/eLife.30094 Medline

56. A. Luqman-Fatah, Y. Watanabe, K. Uno, F. Ishikawa, J. V. Moran, T. Miyoshi, The interferon stimulated gene-encoded protein HELZ2 inhibits human LINE-1 retrotransposition and LINE-1 RNA-mediated type I interferon induction. *Nat. Commun.* **14**, 203 (2023). doi:10.1038/s41467-022-35757-6 Medline

57. J. B. Moldovan, J. V. Moran, The Zinc-Finger Antiviral Protein ZAP Inhibits LINE and Alu Retrotransposition. *PLOS Genet.* **11**, e1005121 (2015). doi:10.1371/journal.pgen.1005121 Medline

58. J. L. Goodier, L. E. Cheung, H. H. Kazazian Jr., Mapping the LINE1 ORF1 protein interactome reveals associated inhibitors of human retrotransposition. *Nucleic Acids Res.* **41**, 7401–7419 (2013). doi:10.1093/nar/gkt512 Medline

59. J. Abramson, J. Adler, J. Dunger, R. Evans, T. Green, A. Pritzel, O. Ronneberger, L. Willmore, A. J. Ballard, J. Bambrick, S. W. Bodenstein, D. A. Evans, C.-C. Hung, M. O'Neill, D. Reiman, K. Tunyasuvunakool, Z. Wu, A. Žemgulytė, E. Arvaniti, C. Beattie, O. Bertolli, A. Bridgland, A. Cherepanov, M. Congreve, A. I. Cowen-Rivers, A. Cowie, M. Figurnov, F. B. Fuchs, H. Gladman, R. Jain, Y. A. Khan, C. M. R. Low, K. Perlin, A. Potapenko, P. Savy, S. Singh, A. Stecula, A. Thillaisundaram, C. Tong, S. Yakneen, E. D. Zhong, M. Zielinski, A. Žídek, V. Bapst, P. Kohli, M. Jaderberg, D. Hassabis, J. M. Jumper, Accurate structure prediction of biomolecular interactions with AlphaFold 3. *Nature* **630**, 493–500 (2024). doi:10.1038/s41586-024-07487-w Medline

60. A. González-Magaña, F. J. Blanco, Human PCNA Structure, Function and Interactions. *Biomolecules* **10**, 570 (2020). doi:10.3390/biom10040570 Medline

61. A. B. Sachs, R. W. Davis, The poly(A) binding protein is required for poly(A) shortening and 60S ribosomal subunit-dependent translation initiation. *Cell* **58**, 857–867 (1989). doi:10.1016/0092-8674(89)90938-0 Medline

62. G. Caponigro, R. Parker, Multiple functions for the poly(A)-binding protein in mRNA decapping and deadenylation in yeast. *Genes Dev.* **9**, 2421–2432 (1995). doi:10.1101/gad.9.19.2421 Medline

63. J. M. Coller, N. K. Gray, M. P. Wickens, mRNA stabilization by poly(A) binding protein is independent of poly(A) and requires translation. *Genes Dev.* **12**, 3226–3235 (1998). doi:10.1101/gad.12.20.3226 Medline

64. A. C. Goldstrohm, M. Wickens, Multifunctional deadenylase complexes diversify mRNA control. *Nat. Rev. Mol. Cell Biol.* **9**, 337–344 (2008). doi:10.1038/nrm2370 Medline

65. L. Weill, E. Belloc, F.-A. Bava, R. Méndez, Translational control by changes in poly(A) tail length: Recycling mRNAs. *Nat. Struct. Mol. Biol.* **19**, 577–585 (2012). doi:10.1038/nsmb.2311 Medline

66. H. Yi, J. Park, M. Ha, J. Lim, H. Chang, V. N. Kim, PABP Cooperates with the CCR4-NOT Complex to Promote mRNA Deadenylation and Block Precocious Decay. *Mol. Cell* **70**, 1081–1088.e5 (2018). doi:10.1016/j.molcel.2018.05.009 Medline

67. M. W. Webster, Y.-H. Chen, J. A. W. Stowell, N. Alhusaini, T. Sweet, B. R. Graveley, J. Coller, L. A. Passmore, mRNA Deadenylation Is Coupled to Translation Rates by the Differential Activities of Ccr4-Not Nucleases. *Mol. Cell* **70**, 1089–1100.e8 (2018). doi:10.1016/j.molcel.2018.05.033 Medline

68. A. L. Nicholson, A. E. Pasquinelli, Tales of Detailed Poly(A) Tails. *Trends Cell Biol.* **29**, 191–200 (2019). doi:10.1016/j.tcb.2018.11.002 Medline

69. A. B. Sachs, R. W. Davis, R. D. Kornberg, A single domain of yeast poly(A)-binding protein is necessary and sufficient for RNA binding and cell viability. *Mol. Cell. Biol.* **7**, 3268–3276 (1987). doi:10.1128/mcb.7.9.3268-3276.1987 Medline

70. U. Kühn, T. Pieler, Xenopus poly(A) binding protein: Functional domains in RNA binding and protein-protein interaction. *J. Mol. Biol.* **256**, 20–30 (1996). doi:10.1006/jmbi.1996.0065 Medline

71. U. Kühn, E. Wahle, Structure and function of poly(A) binding proteins. *Biochim. Biophys. Acta* **1678**, 67–84 (2004). doi:10.1016/j.bbaexp.2004.03.008 Medline

72. I. A. Eliseeva, D. N. Lyabin, L. P. Ovchinnikov, Poly(A)-binding proteins: Structure, domain organization, and activity regulation. *Biochemistry* **78**, 1377–1391 (2013). doi:10.1134/S0006297913130014 Medline

73. R. C. Deo, J. B. Bonanno, N. Sonenberg, S. K. Burley, Recognition of polyadenylate RNA by the poly(A)-binding protein. *Cell* **98**, 835–845 (1999). doi:10.1016/S0092-8674(00)81517-2 Medline

74. W. Wei, N. Gilbert, S. L. Ooi, J. F. Lawler, E. M. Ostertag, H. H. Kazazian, J. D. Boeke, J. V. Moran, Human L1 Retrotransposition: *cis* Preference versus *trans* Complementation. *Mol. Cell. Biol.* **21**, 1429–1439 (2001). doi:10.1128/MCB.21.4.1429-1439.2001 Medline

75. J. D. Boeke, LINEs and *Alus*—The polyA connection. *Nat. Genet.* **16**, 6–7 (1997). doi:10.1038/ng0597-6 Medline

76. D. A. Mangus, M. C. Evans, A. Jacobson, Poly(A)-binding proteins: Multifunctional scaffolds for the post-transcriptional control of gene expression. *Genome Biol.* **4**, 223 (2003). doi:10.1186/gb-2003-4-7-223 Medline

77. R. Sawazaki, S. Imai, M. Yokogawa, N. Hosoda, S. I. Hoshino, M. Mio, K. Mio, I. Shimada, M. Osawa, Characterization of the multimeric structure of poly(A)-binding protein on a poly(A) tail. *Sci. Rep.* **8**, 1455 (2018). doi:10.1038/s41598-018-19659-6 Medline

78. J. Park, M. Kim, H. Yi, K. Baeg, Y. Choi, Y. S. Lee, J. Lim, V. N. Kim, Short poly(A) tails are protected from deadenylation by the LARP1-PABP complex. *Nat. Struct. Mol. Biol.* **30**, 330–338 (2023). doi:10.1038/s41594-023-00930-y Medline

79. M. Dewannieux, T. Heidmann, Role of poly(A) tail length in Alu retrotransposition. *Genomics* **86**, 378–381 (2005). doi:10.1016/j.ygeno.2005.05.009 Medline

80. P. Mita, A. Wudzinska, X. Sun, J. Andrade, S. Nayak, D. J. Kahler, S. Badri, J. LaCava, B. Ueberheide, C. Y. Yun, D. Fenyö, J. D. Boeke, LINE-1 protein localization and functional dynamics during the cell cycle. *eLife* **7**, e30058 (2018). doi:10.7554/eLife.30058 Medline

81. D. A. Flasch, Á. Macia, L. Sánchez, M. Ljungman, S. R. Heras, J. L. García-Pérez, T. E. Wilson, J. V. Moran, Genome-wide *de novo* L1 Retrotransposition Connects Endonuclease Activity with Replication. *Cell* **177**, 837–851.e28 (2019). doi:10.1016/j.cell.2019.02.050 Medline

82. T. Sultana, D. van Essen, O. Siol, M. Bailly-Bechet, C. Philippe, A. Zine El Aabidine, L. Pioger, P. Nigumann, S. Saccani, J.-C. Andrau, N. Gilbert, G. Cristofari, The Landscape of L1 Retrotransposons in the Human Genome Is Shaped by Pre-insertion Sequence Biases and Post-insertion Selection. *Mol. Cell* **74**, 555–570.e7 (2019). doi:10.1016/j.molcel.2019.02.036 Medline

83. S. Trowitzsch, C. Bieniossek, Y. Nie, F. Garzoni, I. Berger, New baculovirus expression tools for recombinant protein complex production. *J. Struct. Biol.* **172**, 45–54 (2010). doi:10.1016/j.jsb.2010.02.010 Medline

84. Y. He, J. Fang, D. J. Taatjes, E. Nogales, Structural visualization of key steps in human transcription initiation. *Nature* **495**, 481–486 (2013). doi:10.1038/nature11991 Medline

85. M. Bokori-Brown, T. G. Martin, C. E. Naylor, A. K. Basak, R. W. Titball, C. G. Savva, Cryo-EM structure of lysenin pore elucidates membrane insertion by an aerolysin family protein. *Nat. Commun.* **7**, 11293 (2016). doi:10.1038/ncomms11293 Medline

86. A. Boland, T. G. Martin, Z. Zhang, J. Yang, X. C. Bai, L. Chang, S. H. W. Scheres, D. Barford, Cryo-EM structure of a metazoan separase-securin complex at near-atomic resolution. *Nat. Struct. Mol. Biol.* **24**, 414–418 (2017). doi:10.1038/nsmb.3386 Medline

87. A. Rohou, N. Grigorieff, CTFFIND4: Fast and accurate defocus estimation from electron micrographs. *J. Struct. Biol.* **192**, 216–221 (2015). doi:10.1016/j.jsb.2015.08.008 Medline

88. T. Bepler, A. Morin, M. Rapp, J. Brasch, L. Shapiro, A. J. Noble, B. Berger, Positive-unlabeled convolutional neural networks for particle picking in cryo-electron micrographs. *Nat. Methods* **16**, 1153–1160 (2019). doi:10.1038/s41592-019-0575-8 Medline

89. J. Zivanov, T. Nakane, S. H. W. Scheres, Estimation of high-order aberrations and anisotropic magnification from cryo-EM data sets in *RELION*-3.1. *IUCrJ* **7**, 253–267 (2020). doi:10.1107/S2052252520000081 Medline

90. J. Zivanov, T. Nakane, S. H. W. Scheres, A Bayesian approach to beam-induced motion correction in cryo-EM single-particle analysis. *IUCrJ* **6**, 5–17 (2019). doi:10.1107/S205225251801463X Medline

91. A. Punjani, J. L. Rubinstein, D. J. Fleet, M. A. Brubaker, cryoSPARC: Algorithms for rapid unsupervised cryo-EM structure determination. *Nat. Methods* **14**, 290–296 (2017). doi:10.1038/nmeth.4169 Medline

92. A. Punjani, H. Zhang, D. J. Fleet, Non-uniform refinement: Adaptive regularization improves single-particle cryo-EM reconstruction. *Nat. Methods* **17**, 1214–1221 (2020). doi:10.1038/s41592-020-00990-8 Medline

93. D. Asarnow, E. Palovcak, Y. Cheng, asarnow/pyem: UCSF pyem v0.5, Zenodo (2019). doi:10.5281/zenodo.3576630

94. S. Chaaban, sami-chaaban/starparser: v1.38, Zenodo (2022). doi:10.5281/zenodo.6792794

95. T. Nakane, D. Kimanius, E. Lindahl, S. H. Scheres, Characterisation of molecular motions in cryo-EM single-particle data by multi-body refinement in RELION. *eLife* **7**, e36861 (2018). doi:10.7554/eLife.36861 Medline

96. T. Nakane, S. H. W. Scheres, Multi-body Refinement of Cryo-EM Images in RELION. *Methods Mol. Biol.* **2215**, 145–160 (2021). doi:10.1007/978-1-0716-0966-8_7 Medline

97. Y. Z. Tan, P. R. Baldwin, J. H. Davis, J. R. Williamson, C. S. Potter, B. Carragher, D. Lyumkis, Addressing preferred specimen orientation in single-particle cryo-EM through tilting. *Nat. Methods* **14**, 793–796 (2017). doi:10.1038/nmeth.4347 Medline

98. J. Jumper, R. Evans, A. Pritzel, T. Green, M. Figurnov, O. Ronneberger, K. Tunyasuvunakool, R. Bates, A. Žídek, A. Potapenko, A. Bridgland, C. Meyer, S. A. A. Kohl, A. J. Ballard, A. Cowie, B. Romera-Paredes, S. Nikolov, R. Jain, J. Adler, T. Back, S. Petersen, D. Reiman, E. Clancy, M. Zielinski, M. Steinegger, M. Pacholska, T. Berghammer, S. Bodenstein, D. Silver, O. Vinyals, A. W. Senior, K. Kavukcuoglu, P. Kohli, D. Hassabis, Highly accurate protein structure prediction with AlphaFold. *Nature* **596**, 583–589 (2021). doi:10.1038/s41586-021-03819-2 Medline

99. T. I. Croll, *ISOLDE*: A physically realistic environment for model building into low-resolution electron-density maps. *Acta Crystallogr. D Struct. Biol.* **74**, 519–530 (2018). doi:10.1107/S2059798318002425 Medline

100. A. Casañal, B. Lohkamp, P. Emsley, Current developments in *Coot* for macromolecular model building of Electron Cryo-microscopy and Crystallographic Data. *Protein Sci.* **29**, 1069–1078 (2020). doi:10.1002/pro.3791 Medline

101. G. N. Murshudov, P. Skubák, A. A. Lebedev, N. S. Pannu, R. A. Steiner, R. A. Nicholls, M. D. Winn, F. Long, A. A. Vagin, *REFMAC5* for the refinement of macromolecular crystal structures. *Acta Crystallogr. D Biol. Crystallogr.* **67**, 355–367 (2011). doi:10.1107/S0907444911001314 Medline

102. D. Liebschner, P. V. Afonine, M. L. Baker, G. Bunkóczi, V. B. Chen, T. I. Croll, B. Hintze, L.-W. Hung, S. Jain, A. J. McCoy, N. W. Moriarty, R. D. Oeffner, B. K. Poon, M. G.

Prisant, R. J. Read, J. S. Richardson, D. C. Richardson, M. D. Sammito, O. V. Sobolev, D. H. Stockwell, T. C. Terwilliger, A. G. Urzhumtsev, L. L. Videau, C. J. Williams, P. D. Adams, Macromolecular structure determination using X-rays, neutrons and electrons: Recent developments in *Phenix*. *Acta Crystallogr. D Biol. Crystallogr.* **75**, 861–877 (2019). doi:10.1107/S2059798319011471 Medline

103. K. Yamashita, C. M. Palmer, T. Burnley, G. N. Murshudov, Cryo-EM single-particle structure refinement and map calculation using *Servalcat*. *Acta Crystallogr. D Struct. Biol.* **77**, 1282–1291 (2021). doi:10.1107/S2059798321009475 Medline

104. R. A. Nicholls, F. Long, G. N. Murshudov, Low-resolution refinement tools in *REFMAC5*. *Acta Crystallogr. D Biol. Crystallogr.* **68**, 404–417 (2012). doi:10.1107/S090744491105606X Medline

105. A. Brown, F. Long, R. A. Nicholls, J. Toots, P. Emsley, G. Murshudov, Tools for macromolecular model building and refinement into electron cryo-microscopy reconstructions. *Acta Crystallogr. D Biol. Crystallogr.* **71**, 136–153 (2015). doi:10.1107/S1399004714021683 Medline

106. E. C. Meng, T. D. Goddard, E. F. Pettersen, G. S. Couch, Z. J. Pearson, J. H. Morris, T. E. Ferrin, UCSF ChimeraX: Tools for structure building and analysis. *Protein Sci.* **32**, e4792 (2023). doi:10.1002/pro.4792 Medline

107. The UniProt Consortium, UniProt: The Universal Protein Knowledgebase in 2023. *Nucleic Acids Res.* **51**, D523–D531 (2023). doi:10.1093/nar/gkac1052 Medline

108. S. Boissinot, A. Sookdeo, The Evolution of LINE-1 in Vertebrates. *Genome Biol. Evol.* **8**, 3485–3507 (2016). doi:10.1093/gbe/evw247 Medline

109. F. Sievers, A. Wilm, D. Dineen, T. J. Gibson, K. Karplus, W. Li, R. Lopez, H. McWilliam, M. Remmert, J. Söding, J. D. Thompson, D. G. Higgins, Fast, scalable generation of high-quality protein multiple sequence alignments using Clustal Omega. *Mol. Syst. Biol.* **7**, 539 (2011). doi:10.1038/msb.2011.75 Medline

110. G. Ghanim, H. Hu, J. Boulanger, T. H. D. Nguyen, Structural mechanism of LINE-1 target-primed reverse transcription, Zenodo (2025). doi:10.5281/zenodo.14840913

111. A. Prestel, N. Wichmann, J. M. Martins, R. Marabini, N. Kassem, S. S. Broendum, M. Otterlei, O. Nielsen, M. Willemoës, M. Ploug, W. Boomsma, B. B. Kragelund, The PCNA interaction motifs revisited: Thinking outside the PIP-box. *Cell. Mol. Life Sci.* **76**, 4923–4943 (2019). doi:10.1007/s00018-019-03150-0 Medline

112. D. Kimanius, L. Dong, G. Sharov, T. Nakane, S. H. W. Scheres, New tools for automated cryo-EM single-particle analysis in RELION-4.0. *Biochem. J.* **478**, 4169–4185 (2021). doi:10.1042/BCJ20210708 Medline

113. A. Punjani, D. J. Fleet, 3D variability analysis: Resolving continuous flexibility and discrete heterogeneity from single particle cryo-EM. *J. Struct. Biol.* **213**, 107702 (2021). doi:10.1016/j.jsb.2021.107702 Medline

114. S. Li, W. K. Olson, X.-J. Lu, Web 3DNA 2.0 for the analysis, visualization, and modeling of 3D nucleic acid structures. *Nucleic Acids Res.* **47**, W26–W34 (2019). doi:10.1093/nar/gkz394 Medline
